# A distributional approach to fractional Sobolev spaces and fractional variation: asymptotics I

**DOI:** 10.1007/s13163-022-00429-y

**Published:** 2022-06-20

**Authors:** Giovanni E. Comi, Giorgio Stefani

**Affiliations:** 1 Dipartimento di Matematica, Università di Pisa, Largo Bruno Pontecorvo 5, 56127 Pisa, Italy; 2 Department Mathematik und Informatik, Universität Basel, Spiegelgasse 1, 4051 Basel, Switzerland

**Keywords:** Function with bounded fractional variation, Fractional perimeter, Fractional derivative, Fractional gradient, Fractional divergence, Gamma-convergence, 26A33, 26B30, 28A33

## Abstract

We continue the study of the space $BV^\alpha ({\mathbb {R}}^n)$ of functions with bounded fractional variation in ${\mathbb {R}}^n$ of order $\alpha \in (0,1)$ introduced in our previous work (Comi and Stefani in J Funct Anal 277(10):3373–3435, 2019). After some technical improvements of certain results of Comi and Stefani (2019) which may be of some separated insterest, we deal with the asymptotic behavior of the fractional operators involved as $\alpha \rightarrow 1^-$. We prove that the $\alpha$-gradient of a $W^{1,p}$-function converges in $L^p$ to the gradient for all $p\in [1,+\infty )$ as $\alpha \rightarrow 1^-$. Moreover, we prove that the fractional $\alpha$-variation converges to the standard De Giorgi’s variation both pointwise and in the $\Gamma$-limit sense as $\alpha \rightarrow 1^-$. Finally, we prove that the fractional $\beta$-variation converges to the fractional $\alpha$-variation both pointwise and in the $\Gamma$-limit sense as $\beta \rightarrow \alpha ^-$ for any given $\alpha \in (0,1)$.

## Introduction

### A distributional approach to fractional variation

In our previous work [27], we introduced the space $BV^\alpha ({\mathbb {R}}^n)$ of functions with bounded fractional variation in ${\mathbb {R}}^n$ of order $\alpha \in (0,1)$. Precisely, a function $f\in L^1({\mathbb {R}}^n)$ belongs to the space $BV^\alpha ({\mathbb {R}}^n)$ if its *fractional* $\alpha$-*variation*

1.1 $$\begin{aligned} |D^\alpha f|({\mathbb {R}}^n):= \sup \Bigg \{\int _{{\mathbb {R}}^n} f\,\mathrm {div}^\alpha \varphi \,dx : \varphi \in C^\infty _c({\mathbb {R}}^n;{\mathbb {R}}^n),\ \Vert \varphi \Vert _{L^\infty ({\mathbb {R}}^n;\,{\mathbb {R}}^n)}\le 1\Bigg \}\qquad \end{aligned}$$

is finite. Here

1.2 $$\begin{aligned} \mathrm {div}^\alpha \varphi (x):=\mu _{n,\alpha }\int _{{\mathbb {R}}^n}\frac{(y-x)\cdot (\varphi (y)-\varphi (x))}{|y-x|^{n+\alpha +1}}\,dy, \qquad x\in {\mathbb {R}}^n, \end{aligned}$$

is the *fractional* $\alpha$-*divergence* of $\varphi \in C^\infty _c({\mathbb {R}}^n;{\mathbb {R}}^n)$, where

1.3 $$\begin{aligned} \mu _{n, \alpha } := 2^{\alpha } \pi ^{- \frac{n}{2}} \frac{\Gamma \left( \frac{n + \alpha + 1}{2} \right) }{\Gamma \left( \frac{1 - \alpha }{2} \right) } \end{aligned}$$

for any given $\alpha \in (0,1)$. The operator $\mathrm {div}^\alpha$ was introduced in [72] as the natural *dual* operator of the much more studied *fractional* $\alpha$-*gradient*

1.4 $$\begin{aligned} \nabla ^\alpha f(x):=\mu _{n,\alpha }\int _{{\mathbb {R}}^n}\frac{(y-x)(f(y)-f(x))}{|y-x|^{n+\alpha +1}}\,dy, \qquad x\in {\mathbb {R}}^n, \end{aligned}$$

defined for all $f\in C^\infty _c({\mathbb {R}}^n)$. For an account on the existing literature on the operator $\nabla ^\alpha$, see [68,  Section 1]. Here we only refer to [66–70, 72–74] for the articles tightly connected to the present work and to [63,  Section 15.2] for an agile presentation of the fractional operators defined in (1.2) and in (1.4) and of some of their elementary properties. According to [70,  Section 1], it is interesting to notice that [42] seems to be the earliest reference for the operator defined in (1.4).

The operators in (1.2) and in (1.4) are *dual* in the sense that

1.5 $$\begin{aligned} \int _{{\mathbb {R}}^n}f\,\mathrm {div}^\alpha \varphi \,dx=-\int _{{\mathbb {R}}^n}\varphi \cdot \nabla ^\alpha f\,dx \end{aligned}$$

for all $f\in C^\infty _c({\mathbb {R}}^n)$ and $\varphi \in C^\infty _c({\mathbb {R}}^n;{\mathbb {R}}^n)$, see [72,  Section 6] and [27,  Lemma 2.5]. Moreover, both operators have good integrability properties when applied to test functions, namely $\nabla ^\alpha f\in L^p({\mathbb {R}}^n)$ and $\mathrm {div}^\alpha \varphi \in L^p({\mathbb {R}}^n;{\mathbb {R}}^n)$ for all $p\in [1,+\infty ]$ for any given $f\in C^\infty _c({\mathbb {R}}^n)$ and $\varphi \in C^\infty _c({\mathbb {R}}^n;{\mathbb {R}}^n)$, see [27,  Corollary 2.3].

The integration-by-part formula (1.5) represents the starting point for the distributional approach to fractional Sobolev spaces and the fractional variation we developed in [27]. In fact, similarly to the classical case, a function $f\in L^1({\mathbb {R}}^n)$ belongs to $BV^\alpha ({\mathbb {R}}^n)$ if and only if there exists a finite vector-valued Radon measure $D^{\alpha } f \in {\mathscr {M}}({\mathbb {R}}^n; {\mathbb {R}}^{n})$ such that

1.6 $$\begin{aligned} \int _{{\mathbb {R}}^{n}} f\, \mathrm {div}^{\alpha } \varphi \, dx = - \int _{{\mathbb {R}}^n} \varphi \cdot d D^{\alpha } f \end{aligned}$$

for all $\varphi \in C^{\infty }_{c}({\mathbb {R}}^n; {\mathbb {R}}^{n})$, see [27,  Theorem 3.2].

Motivated by (1.6) and similarly to the classical case, we can define the *weak fractional* $\alpha$-*gradient* of a function $f\in L^p({\mathbb {R}}^n)$, with $p\in [1,+\infty ]$, as the function $\nabla ^\alpha _w f\in L^1_{{{\,\mathrm{loc}\,}}}({\mathbb {R}}^n;{\mathbb {R}}^n)$ satisfying

$$\begin{aligned} \int _{{\mathbb {R}}^n}f\,\mathrm {div}^\alpha \varphi \, dx =-\int _{{\mathbb {R}}^n}\nabla ^\alpha _w f\cdot \varphi \, dx \end{aligned}$$

for all $\varphi \in C^\infty _c({\mathbb {R}}^n;{\mathbb {R}}^n)$. For $\alpha \in (0,1)$ and $p\in [1,+\infty ]$, we can thus define the *distributional fractional Sobolev space*

1.7 $$\begin{aligned} S^{\alpha ,p}({\mathbb {R}}^n):=\left\{ f\in L^p({\mathbb {R}}^n) : \exists \, \nabla ^\alpha _w f \in L^p({\mathbb {R}}^n;{\mathbb {R}}^n)\right\} \end{aligned}$$

naturally endowed with the norm

1.8 $$\begin{aligned} \Vert f\Vert _{S^{\alpha ,p}({\mathbb {R}}^n)}:=\Vert f\Vert _{L^p({\mathbb {R}}^n)}+\Vert \nabla ^\alpha _w f\Vert _{L^p({\mathbb {R}}^n;\,{\mathbb {R}}^{n})} \qquad \forall f\in S^{\alpha ,p}({\mathbb {R}}^n). \end{aligned}$$

It is interesting to compare the distributional fractional Sobolev spaces $S^{\alpha ,p}({\mathbb {R}}^n)$ with the well-known *fractional Sobolev space* $W^{\alpha ,p}({\mathbb {R}}^n)$, that is, the space

$$\begin{aligned} W^{\alpha ,p}({\mathbb {R}}^n)&:=\Bigg \{f\in L^p({\mathbb {R}}^n) : \\ [f]_{W^{\alpha ,p}({\mathbb {R}}^n)}&:=\left( \int _{{\mathbb {R}}^{n}} \int _{{\mathbb {R}}^{n}} \frac{|f(x)-f(y)|^p}{|x-y|^{n+p\alpha }}\,dx\,dy\right) ^{\frac{1}{p}}<+\infty \Bigg \} \end{aligned}$$

endowed with the norm

$$\begin{aligned} \Vert f\Vert _{W^{\alpha ,p}({\mathbb {R}}^n)}:=\Vert f\Vert _{L^p({\mathbb {R}}^n)}+[f]_{W^{\alpha ,p}({\mathbb {R}}^n)} \qquad \forall f\in W^{\alpha ,p}({\mathbb {R}}^n). \end{aligned}$$

If $p=+\infty$, then $W^{\alpha ,\infty }({\mathbb {R}}^n)$ naturally coincides with the space of bounded $\alpha$-Hölder continuous functions endowed with the usual norm (see [32] for a detailed account on the spaces $W^{\alpha ,p}$).

For the case $p=1$, starting from the very definition of the fractional gradient $\nabla ^\alpha$, it is plain to see that $W^{\alpha ,1}({\mathbb {R}}^n)\subset S^{\alpha ,1}({\mathbb {R}}^n)\subset BV^\alpha ({\mathbb {R}}^n)$ with both (strict) continuous embeddings, see [27,  Theorems 3.18 and 3.25].

For the case $p\in (1,+\infty )$, instead, it is known that $S^{\alpha ,p}({\mathbb {R}}^n)\supset L^{\alpha ,p}({\mathbb {R}}^n)$ with continuous embedding, where $L^{\alpha ,p}({\mathbb {R}}^n)$ is the *Bessel potential space* of parameters $\alpha \in (0,1)$ and $p\in (1,+\infty )$, see [27,  Section 3.9] and the references therein. In the subsequent paper [26], it will be proved that also the inclusion $S^{\alpha ,p}({\mathbb {R}}^n)\subset L^{\alpha ,p}({\mathbb {R}}^n)$ holds continuously, so that the spaces $S^{\alpha ,p}({\mathbb {R}}^n)$ and $L^{\alpha ,p}({\mathbb {R}}^n)$ coincide. In particular, we get the following relations: $S^{\alpha +\varepsilon ,p}({\mathbb {R}}^n)\subset W^{\alpha , p}({\mathbb {R}}^{n})\subset S^{\alpha -\varepsilon ,p}({\mathbb {R}}^n)$ with continuous embeddings for all $\alpha \in (0,1)$, $p\in (1,+\infty )$ and $0<\varepsilon <\min \{\alpha ,1-\alpha \}$, see [69,  Theorem 2.2]; $S^{\alpha , 2}({\mathbb {R}}^{n})=W^{\alpha , 2}({\mathbb {R}}^{n})$ for all $\alpha \in (0, 1)$, see [69,  Theorem 2.2]; $W^{\alpha ,p}({\mathbb {R}}^n)\subset S^{\alpha ,p}({\mathbb {R}}^n)$ with continuous embedding for all $\alpha \in (0,1)$ and $p\in (1,2]$, see [76,  Chapter V, Section 5.3].

In the *geometric regime* $p=1$, our distributional approach to the fractional variation naturally provides a new definition of distributional fractional perimeter. Precisely, for any open set $\Omega \subset {\mathbb {R}}^n$, the *fractional Caccioppoli* $\alpha$-*perimeter in* $\Omega$ of a measurable set $E\subset {\mathbb {R}}^n$ is the *fractional* $\alpha$-*variation* of $\chi _E$ in $\Omega$, i.e.

$$\begin{aligned} |D^\alpha \chi _E|(\Omega )=\sup \Bigg \{\int _E\mathrm {div}^\alpha \varphi \,dx : \varphi \in C^\infty _c(\Omega ;{\mathbb {R}}^n),\ \Vert \varphi \Vert _{L^\infty (\Omega ;{\mathbb {R}}^n)}\le 1\Bigg \}. \end{aligned}$$

Thus, *E* is a set with *finite fractional Caccioppoli* $\alpha$-*perimeter in* $\Omega$ if $|D^\alpha \chi _E|(\Omega )<+\infty$.

Similarly to the aforementioned embedding $W^{\alpha ,1}({\mathbb {R}}^n)\subset BV^\alpha ({\mathbb {R}}^n)$, we have the inequality

1.9 $$\begin{aligned} |D^\alpha \chi _E|(\Omega )\le \mu _{n,\alpha }P_\alpha (E;\Omega ) \end{aligned}$$

for any open set $\Omega \subset {\mathbb {R}}^n$, see [27,  Proposition 4.8], where

1.10 $$\begin{aligned} P_{\alpha }(E; \Omega )&:= \int _{\Omega } \int _{\Omega } \frac{|\chi _{E}(x) - \chi _{E}(y)|}{|x - y|^{n + \alpha }} \, dx \, dy\nonumber \\&\quad + 2 \int _{\Omega } \int _{{\mathbb {R}}^{n} \setminus \Omega } \frac{|\chi _{E}(x) - \chi _{E}(y)|}{|x - y|^{n + \alpha }} \, dx \, dy \end{aligned}$$

is the standard *fractional* $\alpha$-*perimeter* of a measurable set $E\subset {\mathbb {R}}^n$ relative to the open set $\Omega \subset {\mathbb {R}}^n$ (see [28] for an account on the fractional perimeter $P_\alpha$). Note that, by definition, the *fractional* $\alpha$-*perimeter* of *E* in ${\mathbb {R}}^n$ is simply $P_\alpha (E):=P_\alpha (E;{\mathbb {R}}^n)=[\chi _E]_{W^{\alpha ,1}({\mathbb {R}}^n)}$. We remark that inequality (1.9) is strict in most of the cases, as shown in Sect. 2.6 below. This completely answers a question left open in our previous work [27].

### Asymptotics and $\Gamma$-convergence in the standard fractional setting

The fractional Sobolev space $W^{\alpha ,p}({\mathbb {R}}^n)$ can be understood as an ‘intermediate space’ between the space $L^p({\mathbb {R}}^n)$ and the standard Sobolev space $W^{1,p}({\mathbb {R}}^n)$. In fact, $W^{\alpha ,p}({\mathbb {R}}^n)$ can be recovered as a suitable *(real) interpolation space* between the spaces $L^p({\mathbb {R}}^n)$ and $W^{1,p}({\mathbb {R}}^n)$. We refer to [13, 78] for a general introduction on interpolation spaces and to [54] for a more specific treatment of the interpolation space between $L^p({\mathbb {R}}^n)$ and $W^{1,p}({\mathbb {R}}^n)$.

One then naturally expects that, for a sufficiently regular function *f*, the fractional Sobolev seminorm $[f]_{W^{\alpha ,p}({\mathbb {R}}^n)}$, multiplied by a suitable renormalising constant, should tend to $\Vert f\Vert _{L^p({\mathbb {R}}^n)}$ as $\alpha \rightarrow 0^+$ and to $\Vert \nabla f\Vert _{L^p({\mathbb {R}}^n)}$ as $\alpha \rightarrow 1^-$. Indeed, for $p\in [1,+\infty )$, it is known that

1.11 $$\begin{aligned} \lim _{\alpha \rightarrow 0^+}\alpha \,[f]_{W^{\alpha ,p}({\mathbb {R}}^n)}^p =A_{n,p}\,\Vert f\Vert _{L^p({\mathbb {R}}^n)}^p \end{aligned}$$

for all $f\in \bigcup _{\alpha \in (0,1)}W^{\alpha ,p}({\mathbb {R}}^n)$, while

1.12 $$\begin{aligned} \lim _{\alpha \rightarrow 1^-}(1-\alpha )\,[f]_{W^{\alpha ,p}({\mathbb {R}}^n)}^p =B_{n,p}\,\Vert \nabla f\Vert _{L^p({\mathbb {R}}^n; {\mathbb {R}}^{n})}^p \end{aligned}$$

for all $f\in W^{1,p}({\mathbb {R}}^n)$. Here $A_{n,p},B_{n,p}>0$ are two constants depending only on *n*, *p*. The limit (1.11) was proved in [51, 52], while the limit (1.12) was established in [14]. As proved in [30], when $p=1$ the limit (1.12) holds in the more general case of *BV* functions, that is,

1.13 $$\begin{aligned} \lim _{\alpha \rightarrow 1^-}(1-\alpha )\,[f]_{W^{\alpha ,1}({\mathbb {R}}^n)} =B_{n,1}\,|Df|({\mathbb {R}}^n) \end{aligned}$$

for all $f\in BV({\mathbb {R}}^n)$. For a different approach to the limits in (1.11) and in (1.13) based on interpolation techniques, see [54].

The limits (1.12) and (1.13) are special consequences of the celebrated *Bourgain–Brezis–Mironescu* (BBM, for short) *formula*

1.14 $$\begin{aligned}&\lim _{k\rightarrow +\infty } \int _{{\mathbb {R}}^n}\int _{{\mathbb {R}}^n} \frac{|f(x)-f(y)|^p}{|x-y|^p}\,\varrho _k(|x-y|)\,dx\,dy \nonumber \\&\quad = {\left\{ \begin{array}{ll} C_{n,p}\,\Vert \nabla f\Vert _{L^p({\mathbb {R}}^n)}^p &{} \text {for}\ p\in (1,+\infty ),\\ C_{n,1}\,|Df|({\mathbb {R}}^n) &{} \text {for}\ p=1, \end{array}\right. } \end{aligned}$$

where $C_{n,p}>0$ is a constant depending only on *n* and *p*, and $(\varrho _k)_{k\in {\mathbb {N}}}\subset L^1_{{{\,\mathrm{loc}\,}}}([0,+\infty ))$ is a sequence of non-negative radial mollifiers such that

$$\begin{aligned} \int _{{\mathbb {R}}^n}\varrho _k(|x|)\,dx=1\ \text {for all}\, k\in {\mathbb {N}}\,\,\, \text {and}\,\,\, \lim _{k\rightarrow +\infty } \int _\delta ^{+\infty } \varrho _k(r)\,r^{n-1}\,dr=0\ \text {for all}\, \delta >0. \end{aligned}$$

The BBM formula (1.14) has stimulated a profound development in the asymptotic analysis in the fractional framework. On the one hand, the limit (1.14) played a central role in several applications, such as Brezis’ analysis [18] on how to recognize constant functions, innovative characterizations of Sobolev and *BV* functions and $\Gamma$-convergence results [6–8, 11, 16, 48–50, 56–59, 63], approximation of Sobolev norms and image processing [20, 22–24], and last but not least fractional Hardy and Poincaré inequalities [15, 38, 61]. On the other hand, the BBM formula (1.14) has suggested an alternative path to fractional asymptotic analysis by means of interpolation techniques [54, 65]. Recently, the BBM formula in (1.14) has been revisited in terms of a.e. pointwise convergence [21] and in connection with weak $L^p$ quasi-norms [25], where the now-called *Brezis–Van Schaftingen–Yung space*

$$\begin{aligned} BSY^{\alpha ,p}({\mathbb {R}}^n) = \Bigg \{f\in L_{{{\,\mathrm{loc}\,}}}^1({\mathbb {R}}^n) : \left\| \frac{|f(x)-f(y)|}{|x-y|^{\frac{n}{p}+\alpha }}\right\| _{L^p_w({\mathbb {R}}^n\times {\mathbb {R}}^n)}<+\infty \Bigg \}, \end{aligned}$$

defined for $\alpha \in (0,1]$ and $p\in [1,+\infty )$, has opened a very promising perspective in the field [33].

The limits (1.11)–(1.14) have been connected to variational problems [10], generalized to various function spaces, for example Besov spaces [43, 79], Orlicz spaces [2, 36, 37] and magnetic and anisotropic Sobolev spaces [45, 58–60, 75], and extended to various ambient spaces, like compact connected Riemannian manifolds [44], the flat torus [5], Carnot groups [12, 49] and complete doubling metric-measure spaces supporting a local Poincaré inequality [31].

Concerning the fractional perimeter $P_\alpha$ given in (1.10), one has some additional information besides equations (1.11) and (1.13).

On the one hand, thanks to [64,  Theorem 1.2], the fractional $\alpha$-perimeter $P_\alpha$ enjoys the following fractional analogue of Gustin’s *Boxing Inequality* (see [41] and [35,  Corollary 4.5.4]): there exists a dimensional constant $c_n>0$ such that, for any bounded open set $E\subset {\mathbb {R}}^n$, one can find a covering

$$\begin{aligned} E\subset \bigcup _{k\in {\mathbb {N}}} B_{r_k}(x_k) \end{aligned}$$

of open balls such that

1.15 $$\begin{aligned} \sum _{k\in {\mathbb {N}}}r_k^{n-\alpha }\le c_n\alpha (1-\alpha )P_\alpha (E). \end{aligned}$$

Inequality (1.15) bridges the two limiting behaviors given by (1.11) and (1.13) and provides a useful tool for recovering Gagliardo–Nirenberg–Sobolev and Poincaré–Sobolev inequalities that remain stable as the exponent $\alpha \in (0,1)$ approaches the endpoints.

On the other hand, by [3,  Theorem 2], the fractional $\alpha$-perimeter $P_\alpha$ $\Gamma$-converges in $L^1_{{{\,\mathrm{loc}\,}}}({\mathbb {R}}^n)$ to the standard De Giorgi’s perimeter *P* as $\alpha \rightarrow 1^-$, that is, if $\Omega \subset {\mathbb {R}}^n$ is a bounded open set with Lipschitz boundary, then

1.16 $$\begin{aligned} \Gamma (L^1_{{{\,\mathrm{loc}\,}}})\text { -}\lim _{\alpha \rightarrow 1^-}(1-\alpha )\,P_\alpha (E;\Omega ) =2 \omega _{n-1} P(E;\Omega ) \end{aligned}$$

for all measurable sets $E\subset {\mathbb {R}}^n$, where $\omega _{n}$ is the volume of the unit ball in ${\mathbb {R}}^{n}$ (it should be noted that in [3] the authors use a slightly different definition of the fractional $\alpha$-perimeter, since they consider the functional ${\mathcal {J}}_{\alpha }(E, \Omega ) := \frac{1}{2} P_{\alpha }(E, \Omega )$). For a complete account on $\Gamma$-*convergence*, we refer the reader to the monographs [17, 29] (throughout all the paper, with the symbol $\Gamma (X)\text { -}\lim$ we denote the $\Gamma$-convergence in the ambient metric space *X*). The convergence in (1.16), besides giving a $\Gamma$-convergence analogue of the limit in (1.13), is tightly connected with the study of the regularity properties of *non-local minimal surfaces*, that is, (local) minimisers of the fractional $\alpha$-perimeter $P_\alpha$.

### Asymptotics and $\Gamma$-convergence for the fractional $\alpha$-variation as $\alpha \rightarrow 1^-$

The main aim of the present work is to study the asymptotic behavior of the fractional $\alpha$-variation (1.1) as $\alpha \rightarrow 1^-$, both in the pointwise and in the $\Gamma$-convergence sense.

We provide counterparts of the limits (1.12) and (1.13) for the fractional $\alpha$-variation. Indeed, we prove that, if $f\in W^{1,p}({\mathbb {R}}^n)$ for some $p\in [1,+\infty )$, then $f\in S^{\alpha ,p}({\mathbb {R}}^n)$ for all $\alpha \in (0,1)$ and, moreover,

1.17 $$\begin{aligned} \lim _{\alpha \rightarrow 1^-}\Vert \nabla _w^\alpha f-\nabla _w f\Vert _{L^p({\mathbb {R}}^n;\,{\mathbb {R}}^n)}=0. \end{aligned}$$

In the geometric regime $p=1$, we show that if $f\in BV({\mathbb {R}}^n)$ then $f\in BV^\alpha ({\mathbb {R}}^n)$ for all $\alpha \in (0,1)$ and, in addition,

1.18 $$\begin{aligned} D^\alpha f\rightharpoonup Df\ \text {in } {\mathscr {M}}({\mathbb {R}}^n;{\mathbb {R}}^n) \text { and} |D^\alpha f|\rightharpoonup |Df|\ \text {in } {\mathscr {M}}({\mathbb {R}}^n) \text { as}\,\, \alpha \rightarrow 1^- \end{aligned}$$

and

1.19 $$\begin{aligned} \lim _{\alpha \rightarrow 1^-}|D^\alpha f|({\mathbb {R}}^n)=|Df|({\mathbb {R}}^n). \end{aligned}$$

We are also able to treat the case $p=+\infty$. In fact, we prove that if $f\in W^{1,\infty }({\mathbb {R}}^n)$ then $f\in S^{\alpha ,\infty }({\mathbb {R}}^n)$ for all $\alpha \in (0,1)$ and, moreover,

1.20 $$\begin{aligned} \nabla _w^\alpha f\rightharpoonup \nabla _w f \quad \text {in } L^\infty ({\mathbb {R}}^n;{\mathbb {R}}^n) \text { as } \alpha \rightarrow 1^- \end{aligned}$$

and

1.21 $$\begin{aligned} \Vert \nabla _w f\Vert _{L^\infty ({\mathbb {R}}^n;\,{\mathbb {R}}^n)}\le \liminf _{\alpha \rightarrow 1^-}\Vert \nabla ^\alpha _w f\Vert _{L^\infty ({\mathbb {R}}^n;\,{\mathbb {R}}^n)}. \end{aligned}$$

We refer the reader to Theorem 4.9, Theorem 4.11 and Theorem 4.12 below for the precise statements. We warn the reader that the symbol ‘$\rightharpoonup$’ appearing in (1.18) and (1.20) denotes the weak*-convergence, see Sect. 2.1 below for the notation.

Some of the above results were partially announced in [71]. In a similar perspective, we also refer to the work [53], where the authors proved convergence results for non-local gradient operators on *BV* functions defined on bounded open sets with smooth boundary. The approach developed in [53] is however completely different from the asymptotic analysis we presently perform for the fractional operator defined in (1.4), since the boundedness of the domain of definition of the integral operators considered in [53] plays a crucial role.

Notice that the renormalising factor $(1-\alpha )^\frac{1}{p}$ is not needed in the limits (1.17)–(1.21), contrarily to what happened for the limits (1.12) and (1.13). In fact, this difference should not come as a surprise, since the constant $\mu _{n,\alpha }$ in (1.3), encoded in the definition of the operator $\nabla ^\alpha$, satisfies

1.22 $$\begin{aligned} \mu _{n,\alpha }\sim \frac{1-\alpha }{\omega _n} \quad \text {as } \alpha \rightarrow 1^-, \end{aligned}$$

and thus plays a similar role of the factor $(1-\alpha )^\frac{1}{p}$ in the limit as $\alpha \rightarrow 1^-$. Thus, differently from our previous work [27], the constant $\mu _{n,\alpha }$ appearing in the definition of the operators $\nabla ^\alpha$ and $\mathrm {div}^\alpha$ is of crucial importance in the asymptotic analysis developed in the present paper.

Another relevant aspect of our approach is that convergence as $\alpha \rightarrow 1^-$ holds true not only for the total energies, but also at the level of differential operators, in the strong sense when $p\in (1,+\infty )$ and in the weak* sense for $p=1$ and $p=+\infty$. In simpler terms, the *non-local* fractional $\alpha$-gradient $\nabla ^\alpha$ converges to the *local* gradient $\nabla$ as $\alpha \rightarrow 1^-$ in the most natural way every time the limit is well defined.

We also provide a counterpart of (1.16) for the fractional $\alpha$-variation as $\alpha \rightarrow 1^-$. Precisely, we prove that, if $\Omega \subset {\mathbb {R}}^n$ is a bounded open set with Lipschitz boundary, then

1.23 $$\begin{aligned} \Gamma (L^1_{{{\,\mathrm{loc}\,}}})\text { -}\lim _{\alpha \rightarrow 1^-}|D^\alpha \chi _E|(\Omega ) =P(E;\Omega ) \end{aligned}$$

for all measurable set $E\subset {\mathbb {R}}^n$, see Theorem 4.16. In view of (1.9), one may ask whether the $\Gamma \text { -}\limsup$ inequality in (1.23) could be deduced from the $\Gamma \text { -}\limsup$ inequality in (1.16). In fact, by employing (1.9) together with (1.16) and (1.22), one can estimate

$$\begin{aligned} \Gamma (L^1_{{{\,\mathrm{loc}\,}}})\text { -}\limsup _{\alpha \rightarrow 1^-}|D^\alpha \chi _E|(\Omega ) \le \Gamma (L^1_{{{\,\mathrm{loc}\,}}})\text { -}\limsup _{\alpha \rightarrow 1^-}\mu _{n, \alpha } P_{\alpha }(E, \Omega ) = \frac{2 \omega _{n - 1}}{\omega _{n}} P(E, \Omega ). \end{aligned}$$

However, we have $\frac{2\omega _{n - 1}}{\omega _{n}} > 1$ for any $n \ge 2$ and thus the $\Gamma \text { -}\limsup$ inequality in (1.23) follows from the $\Gamma \text { -}\limsup$ inequality in (1.16) only in the case $n=1$. In a similar way, one sees that the $\Gamma \text { -}\liminf$ inequality in (1.23) implies the $\Gamma \text { -}\liminf$ inequality in (1.16) only in the case $n=1$.

Besides the counterpart of (1.16), our approach allows to prove that $\Gamma$-convergence holds true also at the level of functions. Indeed, if $f\in BV({\mathbb {R}}^n)$ and $\Omega \subset {\mathbb {R}}^n$ is an open set such that either $\Omega$ is bounded with Lipschitz boundary or $\Omega ={\mathbb {R}}^n$, then

1.24 $$\begin{aligned} \Gamma (L^1)\text { -}\lim _{\alpha \rightarrow 1^-}|D^\alpha f|(\Omega ) =|D f|(\Omega ). \end{aligned}$$

One can regard the limit (1.24) as an analogue of the $\Gamma$-convergence results known in the usual fractional setting, see [57, 62]. We refer the reader to Theorems 4.13, 4.14 and 4.17 for the (even more general) results in this direction. Again, as before and thanks to the asymptotic behavior (1.22), the renormalising factor $(1-\alpha )$ is not needed in the limits (1.23) and (1.24).

As a byproduct of the techniques developed for the asymptotic study of the fractional $\alpha$-variation as $\alpha \rightarrow 1^-$, we are also able to characterize the behavior of the fractional $\beta$-variation as $\beta \rightarrow \alpha ^-$, for any given $\alpha \in (0,1)$. On the one hand, if $f\in BV^\alpha ({\mathbb {R}}^n)$, then

$$\begin{aligned} D^\beta f\rightharpoonup D^\alpha f\ \text {in } {\mathscr {M}}({\mathbb {R}}^n;{\mathbb {R}}^n) \text { and}\ |D^\beta f|\rightharpoonup |D^\alpha f|\ \text {in } {\mathscr {M}}({\mathbb {R}}^n) \text { as } \beta \rightarrow \alpha ^- \end{aligned}$$

and, moreover,

$$\begin{aligned} \lim _{\beta \rightarrow \alpha ^-}|D^\beta f|({\mathbb {R}}^n)=|D^\alpha f|({\mathbb {R}}^n), \end{aligned}$$

see Theorem 5.4. On the other hand, if $f\in BV^\alpha ({\mathbb {R}}^n)$ and $\Omega \subset {\mathbb {R}}^n$ is an open set such that either $\Omega$ is bounded and $|D^\alpha f|(\partial \Omega )=0$ or $\Omega ={\mathbb {R}}^n$, then

$$\begin{aligned} \Gamma (L^1)\text { -}\lim _{\beta \rightarrow \alpha ^-}|D^\beta f|(\Omega ) =|D^\alpha f|(\Omega ), \end{aligned}$$

see Theorems 5.5 and 5.6.

### Future developments: asymptotics for the fractional $\alpha$-variation as $\alpha \rightarrow 0^+$

Having in mind the limit (1.11), it would be interesting to understand what happens to the fractional $\alpha$-variation (1.1) as $\alpha \rightarrow 0^+$. Note that

1.25 $$\begin{aligned} \lim _{\alpha \rightarrow 0^+}\mu _{n,\alpha } = \pi ^{- \frac{n}{2}} \frac{\Gamma \left( \frac{n + 1}{2} \right) }{\Gamma \left( \frac{1}{2} \right) } =:\mu _{n,0}, \end{aligned}$$

so there is no renormalization factor as $\alpha \rightarrow 0^+$, differently from (1.22).

At least formally, as $\alpha \rightarrow 0^+$ the fractional $\alpha$-gradient in (1.4) is converging to the operator

1.26 $$\begin{aligned} \nabla ^0 f(x):=\mu _{n,0}\int _{{\mathbb {R}}^n}\frac{(y-x)(f(y)-f(x))}{|y-x|^{n+1}}\,dy, \qquad x\in {\mathbb {R}}^n. \end{aligned}$$

The operator in (1.26) is well defined (in the principal value sense) for all $f\in C^\infty _c({\mathbb {R}}^n)$ and, actually, coincides with the well-known vector-valued *Riesz transform* *Rf*, see [39,  Section 5.1.4] and [76,  Chapter 3]. Similarly, the fractional $\alpha$-divergence in (1.2) is formally converging to the operator

1.27 $$\begin{aligned} \mathrm {div}^0\varphi (x):=\mu _{n,0}\int _{{\mathbb {R}}^n}\frac{(y-x)\cdot (\varphi (y)-\varphi (x))}{|y-x|^{n+1}}\,dy, \qquad x\in {\mathbb {R}}^n, \end{aligned}$$

which is well defined (in the principal value sense) for all $\varphi \in C^\infty _c({\mathbb {R}}^n;{\mathbb {R}}^n)$.

In perfect analogy with what we did before, we can introduce the space $BV^0({\mathbb {R}}^n)$ as the space of functions $f\in L^1({\mathbb {R}}^n)$ such that the quantity

$$\begin{aligned} |D^0 f|({\mathbb {R}}^n):=\sup \Bigg \{\int _{{\mathbb {R}}^n}f\,\mathrm {div}^0\varphi \,dx : \varphi \in C^\infty _c({\mathbb {R}}^n;{\mathbb {R}}^n),\ \Vert \varphi \Vert _{L^\infty ({\mathbb {R}}^n;\,{\mathbb {R}}^n)}\le 1\Bigg \} \end{aligned}$$

is finite. Surprisingly (and differently from the fractional $\alpha$-variation, recall [27,  Section 3.10]), it turns out that $|D^0 f|\ll {\mathscr {L}}^{n}$ for all $f\in BV^0({\mathbb {R}}^n)$. More precisely, one can actually prove that $BV^0({\mathbb {R}}^n)=H^1({\mathbb {R}}^n)$, in the sense that $f\in BV^0({\mathbb {R}}^n)$ if and only if $f\in H^1({\mathbb {R}}^n)$, with $D^0f=Rf{\mathscr {L}}^{n}$ in ${\mathscr {M}}({\mathbb {R}}^n;{\mathbb {R}}^n)$. Here

$$\begin{aligned} H^1({\mathbb {R}}^n):=\Bigg \{f\in L^1({\mathbb {R}}^n) : Rf\in L^1({\mathbb {R}}^n;{\mathbb {R}}^n)\Bigg \} \end{aligned}$$

is the (real) *Hardy space*, see [77,  Chapter III] for the precise definition. Thus, it would be interesting to understand for which functions $f\in L^1({\mathbb {R}}^n)$ the fractional $\alpha$-gradient $\nabla ^\alpha f$ tends (in a suitable sense) to the Riesz transform *Rf* as $\alpha \rightarrow 0^+$. Of course, if $Rf\notin L^1({\mathbb {R}}^n;{\mathbb {R}}^n)$, that is, $f\notin H^1({\mathbb {R}}^n)$, then one cannot expect strong convergence in $L^1$ and, instead, may consider the asymptotic behavior of the rescaled fractional gradient $\alpha \,\nabla ^\alpha f$ as $\alpha \rightarrow 0^+$, in analogy with the limit in (1.11). This line of research, as well as the identifications $BV^0=H^1$ and $S^{\alpha ,p}=L^{\alpha ,p}$ mentioned above, it is the subject of the subsequent paper [26].

### Organization of the paper

The paper is organized as follows.

In Sect. 2, after having briefly recalled the definitions and the main properties of the operators $\nabla ^\alpha$ and $\mathrm {div}^\alpha$, we extend certain technical results of [27].

In Sect. 3, we prove several integrability properties of the fractional $\alpha$-gradient and two useful representation formulas for the fractional $\alpha$-variation of functions with bounded De Giorgi’s variation. We are also able to prove similar results for the fractional $\beta$-gradient of functions with bounded fractional $\alpha$-variation, see Sect. 3.4.

In Sect. 4, we study the asymptotic behavior of the fractional $\alpha$-variation as $\alpha \rightarrow 1^-$ and prove pointwise-convergence and $\Gamma$-convergence results, dealing separately with the integrability exponents $p=1$, $p\in (1,+\infty )$ and $p=+\infty$.

In Sect. 5, we show that the fractional $\beta$-variation weakly converges and $\Gamma$-converges to the fractional $\alpha$-variation as $\beta \rightarrow \alpha ^-$ for any $\alpha \in (0,1)$.

In Appendix A, for the reader’s convenience, we state and prove two known results on the truncation and the approximation of *BV* functions and sets with finite perimeter that are used in Sect. 3 and in Sect. 4.

## Preliminaries

### General notation

We start with a brief description of the main notation used in this paper. In order to keep the exposition the most reader-friendly as possible, we retain the same notation adopted in our previous work [27].

Given an open set $\Omega$, we say that a set *E* is compactly contained in $\Omega$, and we write $E\Subset \Omega$, if the ${\overline{E}}$ is compact and contained in $\Omega$. We denote by ${\mathscr {L}}^{n}$ and ${\mathscr {H}}^{\alpha }$ the *n*-dimensional Lebesgue measure and the $\alpha$-dimensional Hausdorff measure on ${\mathbb {R}}^n$ respectively, with $\alpha \ge 0$. Unless otherwise stated, a measurable set is a ${\mathscr {L}}^{n}$-measurable set. We also use the notation $|E|={\mathscr {L}}^{n}(E)$. All functions we consider in this paper are Lebesgue measurable, unless otherwise stated. We denote by $B_r(x)$ the standard open Euclidean ball with center $x\in {\mathbb {R}}^n$ and radius $r>0$. We let $B_r=B_r(0)$. Recall that $\omega _{n} := |B_1|=\pi ^{\frac{n}{2}}/\Gamma \left( \frac{n+2}{2}\right)$ and ${\mathscr {H}}^{n-1}(\partial B_{1}) = n \omega _n$, where $\Gamma$ is Euler’s *Gamma function*, see [9].

We let $\mathrm {GL}(n)\supset \mathrm {O}(n)\supset \mathrm {SO}(n)$ be the *general linear group*, the *orthogonal group* and the *special orthogonal group* respectively. We tacitly identify $\mathrm {GL}(n)\subset {\mathbb {R}}^{n^2}$ with the space of invertible $n\times n$ - matrices and we endow it with the usual Euclidean distance in ${\mathbb {R}}^{n^2}$.

For $k \in {\mathbb {N}}_{0} \cup \{+ \infty \}$ and $m \in {\mathbb {N}}$, we denote by $C^{k}_{c}(\Omega ; {\mathbb {R}}^{m})$ and ${{\,\mathrm{Lip}\,}}_c(\Omega ; {\mathbb {R}}^{m})$ the spaces of $C^{k}$-regular and, respectively, Lipschitz-regular, *m*-vector-valued functions defined on ${\mathbb {R}}^n$ with compact support in $\Omega$.

For any exponent $p\in [1,+\infty ]$, we denote by $L^p(\Omega ;{\mathbb {R}}^m)$ the space of *m*-vector-valued Lebesgue *p*-integrable functions on $\Omega$. For $p\in [1,+\infty ]$, we say that $(f_k)_{k\in {\mathbb {N}}}\subset L^p(\Omega ;{\mathbb {R}}^m)$ *weakly converges* to $f\in L^p(\Omega ;{\mathbb {R}}^m)$, and we write $f_k\rightharpoonup f$ in $L^p(\Omega ;{\mathbb {R}}^m)$ as $k\rightarrow +\infty$, if

2.1 $$\begin{aligned} \lim _{k\rightarrow +\infty }\int _\Omega f_k\cdot \varphi \,dx =\int _\Omega f\cdot \varphi \,dx \end{aligned}$$

for all $\varphi \in L^q(\Omega ;{\mathbb {R}}^m)$, with $q\in [1,+\infty ]$ the *conjugate exponent* of *p*, that is, $\frac{1}{p}+\frac{1}{q}=1$ (with the usual convention $\frac{1}{+\infty }=0$). Note that in the case $p=+\infty$ we make a little abuse of terminology, since the limit in (2.1) actually defines the weak*-convergence in $L^\infty (\Omega ;{\mathbb {R}}^m)$.

We let

$$\begin{aligned} W^{1,p}(\Omega ;{\mathbb {R}}^m):=\Bigg \{u\in L^p(\Omega ;{\mathbb {R}}^m) : [u]_{W^{1,p}(\Omega ;\,{\mathbb {R}}^m)}:=\Vert \nabla u\Vert _{L^p(\Omega ;\,{\mathbb {R}}^{nm})}<+\infty \Bigg \} \end{aligned}$$

be the space of *m*-vector-valued Sobolev functions on $\Omega$, see for instance [46,  Chapter 10] for its precise definition and main properties. We also let

$$\begin{aligned} w^{1,p}(\Omega ;{\mathbb {R}}^m):=\left\{ u\in L^1_{{{\,\mathrm{loc}\,}}}(\Omega ;{\mathbb {R}}^m) : [u]_{W^{1,p}(\Omega ;\,{\mathbb {R}}^m)}<+\infty \right\} . \end{aligned}$$

We let

$$\begin{aligned} BV(\Omega ;{\mathbb {R}}^m):=\left\{ u\in L^1(\Omega ;{\mathbb {R}}^m) : [u]_{BV(\Omega ;\,{\mathbb {R}}^m)}:=|Du|(\Omega )<+\infty \right\} \end{aligned}$$

be the space of *m*-vector-valued functions of bounded variation on $\Omega$, see for instance [4,  Chapter 3] or [34,  Chapter 5] for its precise definition and main properties. We also let

$$\begin{aligned} bv(\Omega ;{\mathbb {R}}^m):=\left\{ u\in L^1_{{{\,\mathrm{loc}\,}}}(\Omega ;{\mathbb {R}}^m) : [u]_{BV(\Omega ;\,{\mathbb {R}}^m)}<+\infty \right\} . \end{aligned}$$

For $\alpha \in (0,1)$ and $p\in [1,+\infty )$, we let

$$\begin{aligned} W^{\alpha ,p}(\Omega ;{\mathbb {R}}^m)&:=\left\{ u\in L^p(\Omega ;{\mathbb {R}}^m) : \!\right. \\&\left. [u]_{W^{\alpha ,p}(\Omega ;\,{\mathbb {R}}^m)} :=\left( \int _\Omega \int _\Omega \frac{|u(x)-u(y)|^p}{|x-y|^{n+p\alpha }}\,dx\,dy\right) ^{\frac{1}{p}}\!<+\infty \right\} \end{aligned}$$

be the space of *m*-vector-valued fractional Sobolev functions on $\Omega$, see [32] for its precise definition and main properties. We also let

$$\begin{aligned} w^{\alpha ,p}(\Omega ; {\mathbb {R}}^m):=\left\{ u\in L^1_{{{\,\mathrm{loc}\,}}}(\Omega ;{\mathbb {R}}^m) : [u]_{W^{\alpha ,p}(\Omega ;\,{\mathbb {R}}^m)}<+\infty \right\} . \end{aligned}$$

For $\alpha \in (0,1)$ and $p=+\infty$, we simply let

$$\begin{aligned} W^{\alpha ,\infty }(\Omega ;{\mathbb {R}}^m):=\left\{ u\in L^\infty (\Omega ;{\mathbb {R}}^m) : \sup _{x,y\in \Omega ,\, x\ne y}\frac{|u(x)-u(y)|}{|x-y|^\alpha }<+\infty \right\} , \end{aligned}$$

so that $W^{\alpha ,\infty }(\Omega ;{\mathbb {R}}^m)=C^{0,\alpha }_b(\Omega ;{\mathbb {R}}^m)$, the space of *m*-vector-valued bounded $\alpha$-Hölder continuous functions on $\Omega$.

We let ${\mathscr {M}}(\Omega ;{\mathbb {R}}^m)$ be the space of *m*-vector-valued Radon measures with finite total variation, precisely

$$\begin{aligned} |\mu |(\Omega ):=\sup \Bigg \{\int _\Omega \varphi \cdot d\mu : \varphi \in C_c^0(\Omega ;{\mathbb {R}}^m),\ \Vert \varphi \Vert _{L^\infty (\Omega ;\,{\mathbb {R}}^m)}\le 1\Bigg \} \end{aligned}$$

for $\mu \in {\mathscr {M}}(\Omega ;{\mathbb {R}}^m)$. We say that $(\mu _k)_{k\in {\mathbb {N}}}\subset {\mathscr {M}}(\Omega ;{\mathbb {R}}^m)$ *weakly converges* to $\mu \in {\mathscr {M}}(\Omega ;{\mathbb {R}}^m)$, and we write $\mu _k\rightharpoonup \mu$ in ${\mathscr {M}}(\Omega ;{\mathbb {R}}^m)$ as $k\rightarrow +\infty$, if

2.2 $$\begin{aligned} \lim _{k\rightarrow +\infty }\int _\Omega \varphi \cdot d\mu _k=\int _\Omega \varphi \cdot d\mu \end{aligned}$$

for all $\varphi \in C_c^0(\Omega ;{\mathbb {R}}^m)$. Note that we make a little abuse of terminology, since the limit in (2.2) actually defines the weak*-convergence in ${\mathscr {M}}(\Omega ;{\mathbb {R}}^m)$.

In order to avoid heavy notation, if the elements of a function space $F(\Omega ;{\mathbb {R}}^m)$ are real-valued (i.e. $m=1$), then we will drop the target space and simply write $F(\Omega )$.

### Basic properties of $\nabla ^\alpha$ and $\mathrm {div}^\alpha$

We recall the non-local operators $\nabla ^\alpha$ and ${{\,\mathrm{div}\,}}^\alpha$ introduced by Šilhavý in [72] (see also our previous work [27]).

Let $\alpha \in (0,1)$ and set

$$\begin{aligned} \mu _{n, \alpha } := 2^{\alpha } \pi ^{- \frac{n}{2}} \frac{\Gamma \left( \frac{n + \alpha + 1}{2} \right) }{\Gamma \left( \frac{1 - \alpha }{2} \right) }. \end{aligned}$$

We let

$$\begin{aligned} \nabla ^{\alpha } f(x) := \mu _{n, \alpha } \lim _{\varepsilon \rightarrow 0} \int _{\{ |z| > \varepsilon \}} \frac{z f(x + z)}{|z|^{n + \alpha + 1}} \, dz \end{aligned}$$

be the *fractional* $\alpha$-*gradient* of $f\in {{\,\mathrm{Lip}\,}}_c({\mathbb {R}}^n)$ at $x\in {\mathbb {R}}^n$. We also let

$$\begin{aligned} \mathrm {div}^{\alpha } \varphi (x) := \mu _{n, \alpha } \lim _{\varepsilon \rightarrow 0} \int _{\{ |z| > \varepsilon \}} \frac{z \cdot \varphi (x + z)}{|z|^{n + \alpha + 1}} \, dz \end{aligned}$$

be the *fractional* $\alpha$-*divergence* of $\varphi \in {{\,\mathrm{Lip}\,}}_c({\mathbb {R}}^n;{\mathbb {R}}^n)$ at $x\in {\mathbb {R}}^n$. The non-local operators $\nabla ^\alpha$ and ${{\,\mathrm{div}\,}}^\alpha$ are well defined in the sense that the involved integrals converge and the limits exist, see [72,  Section 7] and [27,  Section 2]. Moreover, since

$$\begin{aligned} \int _{\{|z|> \varepsilon \}} \frac{z}{|z|^{n + \alpha + 1}} \, dz=0, \qquad \forall \varepsilon >0, \end{aligned}$$

it is immediate to check that $\nabla ^{\alpha }c=0$ for all $c\in {\mathbb {R}}$ and

$$\begin{aligned} \nabla ^{\alpha } f(x)&=\mu _{n, \alpha } \lim _{\varepsilon \rightarrow 0} \int _{\{ |y -x|> \varepsilon \}} \frac{(y - x)}{|y - x|^{n + \alpha + 1}} f(y) \, dy\\&= \mu _{n, \alpha } \lim _{\varepsilon \rightarrow 0} \int _{\{ |x - y| > \varepsilon \}} \frac{(y - x) (f(y) - f(x)) }{|y - x|^{n + \alpha + 1}} \, dy\\&=\mu _{n, \alpha } \int _{{\mathbb {R}}^{n}} \frac{(y - x) (f(y) - f(x)) }{|y - x|^{n + \alpha + 1}} \, dy, \qquad \forall x\in {\mathbb {R}}^n, \end{aligned}$$

for all $f\in {{\,\mathrm{Lip}\,}}_c({\mathbb {R}}^n)$. Analogously, we also have

$$\begin{aligned} \mathrm {div}^{\alpha } \varphi (x)&= \mu _{n, \alpha } \lim _{\varepsilon \rightarrow 0} \int _{\{ |x - y|> \varepsilon \}} \frac{(y - x) \cdot \varphi (y) }{|y - x|^{n + \alpha + 1}} \, dy,\\&= \mu _{n, \alpha } \lim _{\varepsilon \rightarrow 0} \int _{\{ |x - y| > \varepsilon \}} \frac{(y - x) \cdot (\varphi (y) - \varphi (x)) }{|y - x|^{n + \alpha + 1}} \, dy,\\&= \mu _{n, \alpha } \int _{{\mathbb {R}}^{n}} \frac{(y - x) \cdot (\varphi (y) - \varphi (x)) }{|y - x|^{n + \alpha + 1}} \, dy, \qquad \forall x\in {\mathbb {R}}^n, \end{aligned}$$

for all $$\varphi \in {{\,\mathrm{Lip}\,}}_c({\mathbb {R}}^n)$$.

Given $\alpha \in (0,n)$, we let

2.3 $$\begin{aligned} I_{\alpha } u(x) := \frac{\mu _{n, 1-\alpha }}{n - \alpha } \int _{{\mathbb {R}}^{n}} \frac{u(y)}{|x - y|^{n - \alpha }} \, dy, \qquad x\in {\mathbb {R}}^n, \end{aligned}$$

be the *Riesz potential* of order $\alpha \in (0,n)$ of a function $u\in C^\infty _c({\mathbb {R}}^n;{\mathbb {R}}^m)$. We recall that, if $\alpha ,\beta \in (0,n)$ satisfy $\alpha +\beta <n$, then we have the following *semigroup property*

2.4 $$\begin{aligned} I_{\alpha }(I_\beta u)=I_{\alpha +\beta }u \end{aligned}$$

for all $u\in C^\infty _c({\mathbb {R}}^n;{\mathbb {R}}^m)$. In addition, if $1<p<q<+\infty$ satisfy

$$\begin{aligned} \frac{1}{q}=\frac{1}{p}-\frac{\alpha }{n}, \end{aligned}$$

then there exists a constant $C_{n,\alpha ,p}>0$ such that the operator in (2.3) satisfies

2.5 $$\begin{aligned} \Vert I_\alpha u\Vert _{L^q({\mathbb {R}}^n;\,{\mathbb {R}}^m)}\le C_{n,\alpha ,p}\Vert u\Vert _{L^p({\mathbb {R}}^n;\,{\mathbb {R}}^m)} \end{aligned}$$

for all $u\in C^\infty _c({\mathbb {R}}^n;\,{\mathbb {R}}^m)$. As a consequence, the operator in (2.3) extends to a linear continuous operator from $L^p({\mathbb {R}}^n;{\mathbb {R}}^m)$ to $L^q({\mathbb {R}}^n;{\mathbb {R}}^m)$, for which we retain the same notation. For a proof of (2.4) and (2.5), we refer the reader to [76,  Chapter V, Section 1] and to [40,  Section 1.2.1].

We can now recall the following result, see [27,  Proposition 2.2 and Corollary 2.3].

#### Proposition 2.1

Let $\alpha \in (0,1)$. If $f\in {{\,\mathrm{Lip}\,}}_c({\mathbb {R}}^{n})$, then

2.6 $$\begin{aligned} \nabla ^{\alpha } f = I_{1-\alpha }\nabla f = \nabla I_{1 - \alpha } f \end{aligned}$$

and $$\nabla ^{\alpha }f \in L^1({\mathbb {R}}^n; {\mathbb {R}}^{n})\cap L^{\infty }({\mathbb {R}}^{n}; {\mathbb {R}}^{n})$$, with

2.7 $$\begin{aligned} \Vert \nabla ^{\alpha } f \Vert _{L^1({\mathbb {R}}^{n};\,{\mathbb {R}}^n)} \le \mu _{n, \alpha } [f]_{W^{\alpha ,1}({\mathbb {R}}^{n})} \end{aligned}$$

and

2.8 $$\begin{aligned} \Vert \nabla ^{\alpha } f \Vert _{L^{\infty }({\mathbb {R}}^{n};\,{\mathbb {R}}^n)} \le C_{n, \alpha , U} \Vert \nabla f\Vert _{L^{\infty }({\mathbb {R}}^{n};\,{\mathbb {R}}^n)} \end{aligned}$$

for any bounded open set $U\subset {\mathbb {R}}^n$ such that ${{\,\mathrm{supp}\,}}(f) \subset U$, where

2.9 $$\begin{aligned} C_{n, \alpha , U} := \frac{n \mu _{n, \alpha }}{(1 - \alpha )(n + \alpha - 1)} \left( \omega _n{{\,\mathrm{diam}\,}}(U)^{1 - \alpha } +\left( \frac{n \omega _{n}}{n+\alpha -1} \right) ^\frac{n + \alpha - 1}{n}|U|^\frac{1 - \alpha }{n} \right) .\nonumber \\ \end{aligned}$$

Analogously, if $\varphi \in {{\,\mathrm{Lip}\,}}_c({\mathbb {R}}^{n}; {\mathbb {R}}^{n})$ then

2.10 $$\begin{aligned} \mathrm {div}^{\alpha } \varphi = I_{1-\alpha }\mathrm {div}\varphi = \mathrm {div}I_{1 - \alpha } \varphi \end{aligned}$$

and $$\mathrm {div}^{\alpha } \varphi \in L^1({\mathbb {R}}^n)\cap L^{\infty }({\mathbb {R}}^{n})$$, with

2.11 $$\begin{aligned} \Vert \mathrm {div}^{\alpha } \varphi \Vert _{L^1({\mathbb {R}}^{n})} \le \mu _{n, \alpha } [\varphi ]_{W^{\alpha ,1}({\mathbb {R}}^{n};\,{\mathbb {R}}^n)} \end{aligned}$$

and

2.12 $$\begin{aligned} \Vert \mathrm {div}^{\alpha } \varphi \Vert _{L^{\infty }({\mathbb {R}}^{n})} \le C_{n, \alpha , U} \Vert \mathrm {div}\varphi \Vert _{L^{\infty }({\mathbb {R}}^{n})} \end{aligned}$$

for any bounded open set $U\subset {\mathbb {R}}^n$ such that ${{\,\mathrm{supp}\,}}(\varphi ) \subset U$, where $C_{n, \alpha , U}$ is as in (2.9).

### Extension of $\nabla ^\alpha$ and $\mathrm {div}^\alpha$ to ${{\,\mathrm{Lip}\,}}_b$-regular tests

In the following result, we extend the fractional $\alpha$-divergence to ${{\,\mathrm{Lip}\,}}_b$-regular vector fields.

#### Lemma 2.2

(Extension of $\mathrm {div}^\alpha$ to ${{\,\mathrm{Lip}\,}}_b$). Let $\alpha \in (0,1)$. The operator

$$\begin{aligned} \mathrm {div}^\alpha :{{\,\mathrm{Lip}\,}}_b({\mathbb {R}}^n;{\mathbb {R}}^n)\rightarrow L^\infty ({\mathbb {R}}^n) \end{aligned}$$

given by

2.13 $$\begin{aligned} \mathrm {div}^\alpha \varphi (x):=\mu _{n,\alpha }\int _{{\mathbb {R}}^n}\frac{(y-x)\cdot (\varphi (y)-\varphi (x))}{|y-x|^{n+\alpha +1}}\,dy, \quad x\in {\mathbb {R}}^n, \end{aligned}$$

for all $\varphi \in {{\,\mathrm{Lip}\,}}_b({\mathbb {R}}^n;{\mathbb {R}}^n)$, is well defined, with

2.14 $$\begin{aligned} \Vert \mathrm {div}^\alpha \varphi \Vert _{L^\infty ({\mathbb {R}}^n)} \le \frac{2^{1-\alpha }n\omega _n\mu _{n,\alpha }}{\alpha (1-\alpha )}{{\,\mathrm{Lip}\,}}(\varphi )^\alpha \Vert \varphi \Vert _{L^\infty ({\mathbb {R}}^n;\,{\mathbb {R}}^n)}^{1-\alpha }, \end{aligned}$$

and satisfies

2.15 $$\begin{aligned} \begin{aligned} \mathrm {div}^\alpha \varphi (x)&=\mu _{n,\alpha }\lim _{\varepsilon \rightarrow 0^+}\int _{\{|y-x|>\varepsilon \}}\frac{(y-x)\cdot (\varphi (y)-\varphi (x))}{|y-x|^{n+\alpha +1}}\,dy\\&=\mu _{n,\alpha }\lim _{\varepsilon \rightarrow 0^+}\int _{\{|y-x|>\varepsilon \}}\frac{(y-x)\cdot \varphi (y)}{|y-x|^{n+\alpha +1}}\,dy \end{aligned} \end{aligned}$$

for all $x\in {\mathbb {R}}^n$. Moreover, if in addition $I_{1-\alpha }|\mathrm {div}\varphi |\in L^1_{{{\,\mathrm{loc}\,}}}({\mathbb {R}}^n)$, then

2.16 $$\begin{aligned} \mathrm {div}^\alpha \varphi (x)=I_{1-\alpha }\mathrm {div}\varphi (x) \end{aligned}$$

for a.e. $$x\in {\mathbb {R}}^n$$.

#### Proof

We split the proof in two steps.

*Step 1: proof of* (2.13), (2.14) *and* (2.15). Given $x\in {\mathbb {R}}^n$ and $r>0$, we can estimate

$$\begin{aligned} \int _{\{|y-x|\le r\}}\Bigg |\frac{(y-x)\cdot (\varphi (y)-\varphi (x))}{|y-x|^{n+\alpha +1}}\Bigg |\,dy \le n\omega _n\!{{\,\mathrm{Lip}\,}}(\varphi )\int _0^r\varrho ^{-\alpha }\,d\varrho \end{aligned}$$

and

$$\begin{aligned} \int _{\{|y-x|>r\}}\Bigg |\frac{(y-x)\cdot (\varphi (y)-\varphi (x))}{|y-x|^{n+\alpha +1}}\Bigg |\,dy \le 2n\omega _n\Vert \varphi \Vert _{L^\infty ({\mathbb {R}}^n;\,{\mathbb {R}}^n)}\int _r^{+\infty }\varrho ^{-(1+\alpha )}\,d\varrho . \end{aligned}$$

Hence the function in (2.13) is well defined for all $x\in {\mathbb {R}}^n$ and

$$\begin{aligned} \Vert \mathrm {div}^\alpha \varphi \Vert _{L^\infty ({\mathbb {R}}^n)} \le n\omega _n\left( \frac{{{\,\mathrm{Lip}\,}}(\varphi )}{1-\alpha }\,r^{1-\alpha }+\frac{2\Vert \varphi \Vert _{L^\infty ({\mathbb {R}}^n;\,{\mathbb {R}}^n)}}{\alpha }\,r^{-\alpha }\right) , \end{aligned}$$

so that (2.14) follows by optimising the right-hand side in $r>0$. Moreover, since

$$\begin{aligned}&\Bigg |\frac{(y-x)\cdot (\varphi (y)-\varphi (x))}{|y-x|^{n+\alpha +1}}\,\chi _{(\varepsilon ,+\infty )}(|y-x|)\Bigg |\\&\quad \le {{\,\mathrm{Lip}\,}}(\varphi )\,\frac{\chi _{(0,1)}(|y-x|)}{|y-x|^{n+\alpha -1}} +2\Vert \varphi \Vert _{L^\infty ({\mathbb {R}}^n;\,{\mathbb {R}}^n)}\,\frac{\chi _{[1,+\infty )}(|y-x|)}{|y-x|^{n+\alpha }} \in L^1_{x,y}({\mathbb {R}}^n) \end{aligned}$$

and

$$\begin{aligned} \int _{\{|z|>\varepsilon \}}\frac{z}{|z|^{n+\alpha +1}}\,dy=0 \end{aligned}$$

for all $\varepsilon >0$, by Lebesgue’s Dominated Convergence Theorem we immediately get the two equalities in (2.15) for all $x\in {\mathbb {R}}^n$.

*Step 2: proof of* (2.16). Assume that $I_{1-\alpha }|\mathrm {div}\varphi |\in L^1_{{{\,\mathrm{loc}\,}}}({\mathbb {R}}^n)$. Then

2.17 $$\begin{aligned} \frac{|\mathrm {div}\varphi (y)|}{|y-x|^{n+\alpha -1}}\in L^1_y({\mathbb {R}}^n) \end{aligned}$$

for a.e. $x\in {\mathbb {R}}^n$. Hence, by Lebesgue’s Dominated Convergence Theorem, we can write

$$\begin{aligned} I_{1-\alpha }\mathrm {div}\varphi (x) =\mu _{n,\alpha }\lim _{\varepsilon \rightarrow 0^+}\int _{\{|y-x|>\varepsilon \}}\frac{\mathrm {div}\varphi (y)}{|y-x|^{n+\alpha -1}}\,dy \end{aligned}$$

for a.e. $x\in {\mathbb {R}}^n$. Now let $\varepsilon >0$ be fixed and let $R>0$. Again by (2.17) and Lebesgue’s Dominated Convergence Theorem, we have

$$\begin{aligned} \lim _{R\rightarrow +\infty }\int _{\{R> |y-x|> \varepsilon \}}\frac{\mathrm {div}\varphi (y)}{|y-x|^{n+\alpha -1}}\,dy=\int _{\{|y-x|>\varepsilon \}}\frac{\mathrm {div}\varphi (y)}{|y-x|^{n+\alpha -1}}\,dy \end{aligned}$$

for a.e. $x\in {\mathbb {R}}^n$. Moreover, integrating by parts, we get

$$\begin{aligned} \int _{\{R>|y-x|>\varepsilon \}}\frac{\mathrm {div}\varphi (y)}{|y-x|^{n+\alpha -1}}\,dy&=\int _{\{R>|y|>\varepsilon \}}\frac{\mathrm {div}_y\varphi (y+x)}{|y|^{n+\alpha -1}}\,dy\\&=\int _{\{|y|=R\}}\frac{y}{|y|}\frac{\varphi (y+x)}{|y|^{n+\alpha -1}}\,d{\mathscr {H}}^{n-1}(y)\\&\quad -\int _{\{|y|=\varepsilon \}}\frac{y}{|y|}\frac{\varphi (y+x)}{|y|^{n+\alpha -1}}\,d{\mathscr {H}}^{n-1}(y)\\&\quad +\int _{\{R>|y|>\varepsilon \}}\frac{y\cdot \varphi (y+x)}{|y|^{n+\alpha +1}}\,dy \end{aligned}$$

for all $R>0$ and for a.e. $x\in {\mathbb {R}}^n$. Since $\varphi \in L^\infty ({\mathbb {R}}^n;{\mathbb {R}}^n)$, by Lebesgue’s Dominated Convergence Theorem we have

$$\begin{aligned} \lim _{R\rightarrow +\infty }\int _{\{R>|y|>\varepsilon \}}\frac{y\cdot \varphi (y+x)}{|y|^{n+\alpha +1}}\,dy =\int _{\{|y|>\varepsilon \}}\frac{y\cdot \varphi (y+x)}{|y|^{n+\alpha +1}}\,dy \end{aligned}$$

for all $\varepsilon >0$ and all $x\in {\mathbb {R}}^n$. We can also estimate

$$\begin{aligned} \Bigg |\int _{\{|y|=R\}}\frac{y}{|y|}\frac{\varphi (y+x)}{|y|^{n+\alpha -1}}\,d{\mathscr {H}}^{n-1}(y)\Bigg | \le n\omega _n\Vert \varphi \Vert _{L^\infty ({\mathbb {R}}^n;\,{\mathbb {R}}^n)}R^{-\alpha } \end{aligned}$$

for all $R>0$ and all $x\in {\mathbb {R}}^n$. We thus have that

$$\begin{aligned}&\int _{\{|y-x|>\varepsilon \}}\frac{\mathrm {div}\varphi (y)}{|y-x|^{n+\alpha -1}}\,dy\\&\quad =\int _{\{|y|>\varepsilon \}}\frac{y\cdot \varphi (y+x)}{|y|^{n+\alpha +1}}\,dy -\int _{\{|y|=\varepsilon \}}\frac{y}{|y|}\frac{\varphi (y+x)}{|y|^{n+\alpha -1}}\,d{\mathscr {H}}^{n-1}(y) \end{aligned}$$

for all $\varepsilon >0$ and a.e. $x\in {\mathbb {R}}^n$. Since also

$$\begin{aligned} \Bigg |\int _{\{|y|=\varepsilon \}}\frac{y}{|y|}\frac{\varphi (y+x)}{|y|^{n+\alpha -1}}\,d{\mathscr {H}}^{n-1}(y)\Bigg |&=\Bigg |\int _{\{|y|=\varepsilon \}}\frac{y}{|y|}\frac{\varphi (y+x)-\varphi (x)}{|y|^{n+\alpha -1}}\,d{\mathscr {H}}^{n-1}(y)\Bigg |\\&\le n\omega _n{{\,\mathrm{Lip}\,}}(\varphi )\,\varepsilon ^{1-\alpha } \end{aligned}$$

for all $\varepsilon >0$ and $x\in {\mathbb {R}}^n$, we conclude that

$$\begin{aligned} \lim _{\varepsilon \rightarrow 0^+}\int _{\{|y-x|>\varepsilon \}}\frac{\mathrm {div}\varphi (y)}{|y-x|^{n+\alpha -1}}\,dy =\lim _{\varepsilon \rightarrow 0^+}\int _{\{|y-x|>\varepsilon \}}\frac{(y-x)\cdot \varphi (y)}{|y-x|^{n+\alpha +1}}\,dy \end{aligned}$$

for a.e. $$x\in {\mathbb {R}}^n$$, proving (2.16). $$\square $$

We can also extend the fractional $\alpha$-gradient to ${{\,\mathrm{Lip}\,}}_b$-regular functions. The proof is very similar to the one of Lemma 2.2 and is left to the reader.

#### Lemma 2.3

(Extension of $\nabla ^\alpha$ to ${{\,\mathrm{Lip}\,}}_b$). Let $\alpha \in (0,1)$. The operator

$$\begin{aligned} \nabla ^\alpha :{{\,\mathrm{Lip}\,}}_b({\mathbb {R}}^n)\rightarrow L^\infty ({\mathbb {R}}^n;{\mathbb {R}}^n) \end{aligned}$$

given by

$$\begin{aligned} \nabla ^\alpha f(x):=\mu _{n,\alpha }\int _{{\mathbb {R}}^n}\frac{(y-x)\cdot (f(y)-f(x))}{|y-x|^{n+\alpha +1}}\,dy, \quad x\in {\mathbb {R}}^n, \end{aligned}$$

for all $f\in {{\,\mathrm{Lip}\,}}_b({\mathbb {R}}^n)$, is well defined, with

$$\begin{aligned} \Vert \nabla ^\alpha f\Vert _{L^\infty ({\mathbb {R}}^n;\,{\mathbb {R}}^n)} \le \frac{2^{1-\alpha }n\omega _n\mu _{n,\alpha }}{\alpha (1-\alpha )}{{\,\mathrm{Lip}\,}}(f)^\alpha \Vert f\Vert _{L^\infty ({\mathbb {R}}^n)}^{1-\alpha }, \end{aligned}$$

and satisfies

$$\begin{aligned} \begin{aligned} \nabla ^\alpha f(x)&=\mu _{n,\alpha }\lim _{\varepsilon \rightarrow 0^+}\int _{\{|y-x|>\varepsilon \}}\frac{(y-x)\cdot (f(y)-f(x))}{|y-x|^{n+\alpha +1}}\,dy\\&=\mu _{n,\alpha }\lim _{\varepsilon \rightarrow 0^+}\int _{\{|y-x|>\varepsilon \}}\frac{(y-x)\cdot f(y)}{|y-x|^{n+\alpha +1}}\,dy \end{aligned} \end{aligned}$$

for all $x\in {\mathbb {R}}^n$. Moreover, if in addition $I_{1-\alpha }|\nabla f|\in L^1_{{{\,\mathrm{loc}\,}}}({\mathbb {R}}^n)$, then

$$\begin{aligned} \nabla ^\alpha f(x)=I_{1-\alpha }\nabla f(x) \end{aligned}$$

for a.e. $$x\in {\mathbb {R}}^n$$.

### Extended Leibniz’s rules for $\nabla ^\alpha$ and $\mathrm {div}^\alpha$

The following two results extend the validity of Leibniz’s rules proved in [27,  Lemmas 2.6 and 2.7] to ${{\,\mathrm{Lip}\,}}_b$-regular functions and ${{\,\mathrm{Lip}\,}}_b$-regular vector fields. The proofs are very similar to the ones given in [27] and to that of Lemma 2.2, and thus are left to the reader.

#### Lemma 2.4

(Extended Leibniz’s rule for $\nabla ^\alpha$). Let $\alpha \in (0,1)$. If $f\in {{\,\mathrm{Lip}\,}}_b({\mathbb {R}}^n)$ and $\eta \in {{\,\mathrm{Lip}\,}}_c({\mathbb {R}}^n)$, then

$$\begin{aligned} \nabla ^\alpha (\eta f) =\eta \,\nabla ^\alpha f+ f\,\nabla ^\alpha \eta +\nabla ^\alpha _\mathrm{NL}(\eta ,f), \end{aligned}$$

where

$$\begin{aligned} \nabla ^\alpha _\mathrm{NL}(\eta ,f)(x) =\mu _{n,\alpha }\int _{{\mathbb {R}}^n}\frac{(y-x)\cdot (f(y)-f(x))(\eta (y)-\eta (x))}{|y-x|^{n+\alpha +1}}\,dy \end{aligned}$$

for all $x\in {\mathbb {R}}^n$, with

$$\begin{aligned} \Vert \nabla ^\alpha _\mathrm{NL}(\eta ,f)\Vert _{L^\infty ({\mathbb {R}}^n;\,{\mathbb {R}}^n)} \le \frac{2^{2-\alpha }n\omega _n\mu _{n,\alpha }\Vert f\Vert _{L^\infty ({\mathbb {R}}^n)}}{\alpha (1-\alpha )}{{\,\mathrm{Lip}\,}}(\eta )^\alpha \Vert \eta \Vert _{L^\infty ({\mathbb {R}}^n)}^{1-\alpha } \end{aligned}$$

and

$$\begin{aligned} \Vert \nabla ^\alpha _\mathrm{NL}(\eta ,f)\Vert _{L^1({\mathbb {R}}^n;\,{\mathbb {R}}^n)} \le 2\mu _{n,\alpha }\Vert f\Vert _{L^\infty ({\mathbb {R}}^n)}[\eta ]_{W^{\alpha ,1}({\mathbb {R}}^n)}. \end{aligned}$$

#### Lemma 2.5

(Extended Leibniz’s rule for $\mathrm {div}^\alpha$). Let $\alpha \in (0,1)$. If $\varphi \in {{\,\mathrm{Lip}\,}}_b({\mathbb {R}}^n;{\mathbb {R}}^n)$ and $\eta \in {{\,\mathrm{Lip}\,}}_c({\mathbb {R}}^n)$, then

$$\begin{aligned} \mathrm {div}^\alpha (\eta \varphi ) =\eta \,\mathrm {div}^\alpha \varphi +\varphi \cdot \nabla ^\alpha \eta +\mathrm {div}^\alpha _\mathrm{NL}(\eta ,\varphi ), \end{aligned}$$

where

$$\begin{aligned} \mathrm {div}^\alpha _\mathrm{NL}(\eta ,\varphi )(x) =\mu _{n,\alpha }\int _{{\mathbb {R}}^n}\frac{(y-x)\cdot (\varphi (y)-\varphi (x))(\eta (y)-\eta (x))}{|y-x|^{n+\alpha +1}}\,dy \end{aligned}$$

for all $x\in {\mathbb {R}}^n$, with

$$\begin{aligned} \Vert \mathrm {div}^\alpha _\mathrm{NL}(\eta ,\varphi )\Vert _{L^\infty ({\mathbb {R}}^n)} \le \frac{2^{2-\alpha }n\omega _n\mu _{n,\alpha }\Vert \varphi \Vert _{L^\infty ({\mathbb {R}}^n;\,{\mathbb {R}}^n)}}{\alpha (1-\alpha )}{{\,\mathrm{Lip}\,}}(\eta )^\alpha \Vert \eta \Vert _{L^\infty ({\mathbb {R}}^n)}^{1-\alpha } \end{aligned}$$

and

$$\begin{aligned} \Vert \mathrm {div}^\alpha _\mathrm{NL}(\eta ,\varphi )\Vert _{L^1({\mathbb {R}}^n)} \le 2\mu _{n,\alpha }\Vert \varphi \Vert _{L^\infty ({\mathbb {R}}^n;\,{\mathbb {R}}^n)}[\eta ]_{W^{\alpha ,1}({\mathbb {R}}^n)}. \end{aligned}$$

### Extended integration-by-part formulas

We now recall the definition of the space of functions with bounded fractional $\alpha$-variation. Given $\alpha \in (0, 1)$, we let

$$\begin{aligned} BV^\alpha ({\mathbb {R}}^n):=\Bigg \{f\in L^1({\mathbb {R}}^n) : |D^\alpha f|({\mathbb {R}}^n)<+\infty \Bigg \}, \end{aligned}$$

where

$$\begin{aligned} |D^\alpha f|({\mathbb {R}}^n)=\sup \Bigg \{\int _{{\mathbb {R}}^n}f\,\mathrm {div}^\alpha \varphi \,dx : \varphi \in C^\infty _c({\mathbb {R}}^n;{\mathbb {R}}^n),\ \Vert \varphi \Vert _{L^\infty ({\mathbb {R}}^n;{\mathbb {R}}^n)}\le 1\Bigg \} \end{aligned}$$

is the *fractional* $\alpha$-*variation* of $f\in L^1({\mathbb {R}}^n)$. We refer the reader to [27,  Section 3] for the basic properties of this function space. Here we just recall the following result, see [27,  Theorem 3.2 and Proposition 3.6] for the proof.

#### Theorem 2.6

(Structure theorem for $BV^\alpha$ functions). Let $\alpha \in (0,1)$. If $f \in L^{1}({\mathbb {R}}^{n})$, then $f \in BV^{\alpha }({\mathbb {R}}^{n})$ if and only if there exists a finite vector-valued Radon measure $D^{\alpha } f \in {\mathscr {M}}({\mathbb {R}}^{n}; {\mathbb {R}}^{n})$ such that

2.18 $$\begin{aligned} \int _{{\mathbb {R}}^{n}} f\, \mathrm {div}^{\alpha } \varphi \, dx = - \int _{{\mathbb {R}}^n} \varphi \cdot d D^{\alpha } f \end{aligned}$$

for all $$\varphi \in {{\,\mathrm{Lip}\,}}_{c}({\mathbb {R}}^{n}; {\mathbb {R}}^{n})$$.

Thanks to Lemma 2.5, we can actually prove that a function in $BV^\alpha ({\mathbb {R}}^n)$ can be tested against any ${{\,\mathrm{Lip}\,}}_b$-regular vector field.

#### Proposition 2.7

(${{\,\mathrm{Lip}\,}}_b$-regular test for $BV^\alpha$ functions). Let $\alpha \in (0,1)$. If $f\in BV^\alpha ({\mathbb {R}}^n)$, then (2.18) holds for all $\varphi \in {{\,\mathrm{Lip}\,}}_b({\mathbb {R}}^n;{\mathbb {R}}^n)$.

#### Proof

We argue as in the proof of [27,  Theorem 3.8]. Fix $\varphi \in {{\,\mathrm{Lip}\,}}_b({\mathbb {R}}^n;{\mathbb {R}}^n)$ and let $(\eta _R)_{R>0}\subset C^\infty _c({\mathbb {R}}^n)$ be a family of cut-off functions as in [27,  Section 3.3]. On the one hand, since

$$\begin{aligned} \Bigg |\int _{{\mathbb {R}}^n}f\eta _R\,\mathrm {div}^\alpha \varphi \,dx-\int _{{\mathbb {R}}^n}f\,\mathrm {div}^\alpha \varphi \,dx\Bigg | \le \Vert \mathrm {div}^\alpha \varphi \Vert _{L^\infty ({\mathbb {R}}^n)} \int _{{\mathbb {R}}^n}|f|\,(1-\eta _R)\,dx \end{aligned}$$

for all $R>0$, by Lebesgue’s Dominated Convergence Theorem we have

$$\begin{aligned} \lim _{R\rightarrow +\infty }\int _{{\mathbb {R}}^n}f\eta _R\,\mathrm {div}^\alpha \varphi \,dx =\int _{{\mathbb {R}}^n}f\,\mathrm {div}^\alpha \varphi \,dx. \end{aligned}$$

On the other hand, by Lemma 2.5 we can write

$$\begin{aligned} \int _{{\mathbb {R}}^n}f\eta _R\,\mathrm {div}^\alpha \varphi \,dx&=\int _{{\mathbb {R}}^n}f\,\mathrm {div}^\alpha (\eta _R\varphi )\,dx -\int _{{\mathbb {R}}^n}f\,\varphi \cdot \nabla ^\alpha \eta _R\,dx\\&\quad -\int _{{\mathbb {R}}^n}f\,\mathrm {div}^\alpha _\mathrm{NL}(\eta _R,\varphi )\,dx \end{aligned}$$

for all $R>0$. By [27,  Proposition 3.6], we have

$$\begin{aligned} \int _{{\mathbb {R}}^n}f\,\mathrm {div}^\alpha (\eta _R\varphi )\,dx =-\int _{{\mathbb {R}}^n}\eta _R\varphi \cdot dD^\alpha f \end{aligned}$$

for all $R>0$. Since

$$\begin{aligned} \Bigg |\int _{{\mathbb {R}}^n}\eta _R\varphi \cdot dD^\alpha f-\int _{{\mathbb {R}}^n}\varphi \cdot dD^\alpha f\Bigg | \le \Vert \varphi \Vert _{L^\infty ({\mathbb {R}}^n;{\mathbb {R}}^n)}\int _{{\mathbb {R}}^n}(1-\eta _R)\,d|D^\alpha f| \end{aligned}$$

for all $R>0$, by Lebesgue’s Dominated Convergence Theorem (with respect to the finite measure $|D^\alpha f|$) we have

$$\begin{aligned} \lim _{R\rightarrow +\infty }\int _{{\mathbb {R}}^n}\eta _R\varphi \cdot dD^\alpha f =\int _{{\mathbb {R}}^n}\varphi \cdot dD^\alpha f. \end{aligned}$$

Finally, we can estimate

$$\begin{aligned} \Bigg |\int _{{\mathbb {R}}^n}f\,\varphi \cdot \nabla ^\alpha \eta _R\,dx\Bigg | \le \mu _{n,\alpha }\Vert \varphi \Vert _{L^\infty ({\mathbb {R}}^n;{\mathbb {R}}^n)} \int _{{\mathbb {R}}^n}|f(x)|\int _{{\mathbb {R}}^n}\frac{|\eta _R(y)-\eta _R(x)|}{|y-x|^{n+\alpha }}\,dy\,dx \end{aligned}$$

and, similarly,

$$\begin{aligned}&\Bigg |\int _{{\mathbb {R}}^n}f\,\mathrm {div}^\alpha _\mathrm{NL}(\eta _R,\varphi )\,dx\Bigg | \le 2\mu _{n,\alpha }\Vert \varphi \Vert _{L^\infty ({\mathbb {R}}^n;{\mathbb {R}}^n)} \int _{{\mathbb {R}}^n}|f(x)|\int _{{\mathbb {R}}^n}\frac{|\eta _R(y)-\eta _R(x)|}{|y-x|^{n+\alpha }}\,dy\,dx. \end{aligned}$$

By Lebesgue’s Dominated Convergence Theorem, we thus get that

$$\begin{aligned} \lim _{R\rightarrow +\infty }\left( \int _{{\mathbb {R}}^n}f\,\varphi \cdot \nabla ^\alpha \eta _R\,dx +\int _{{\mathbb {R}}^n}f\,\mathrm {div}^\alpha _\mathrm{NL}(\eta _R,\varphi )\,dx\right) =0 \end{aligned}$$

and the conclusion follows. $\square$

Thanks to Lemma 2.4, we can prove that a function in ${{\,\mathrm{Lip}\,}}_b({\mathbb {R}}^n)$ can be tested against any ${{\,\mathrm{Lip}\,}}_c$-regular vector field. The proof is very similar to the one of Proposition 2.7 and is thus left to the reader.

#### Proposition 2.8

(Integration by parts for ${{\,\mathrm{Lip}\,}}_b$-regular functions). Let $\alpha \in (0,1)$. If $f\in {{\,\mathrm{Lip}\,}}_b({\mathbb {R}}^n)$, then

$$\begin{aligned} \int _{{\mathbb {R}}^n}f\,\mathrm {div}^\alpha \varphi \,dx=-\int _{{\mathbb {R}}^n}\varphi \cdot \nabla ^\alpha f\,dx \end{aligned}$$

for all $$\varphi \in {{\,\mathrm{Lip}\,}}_c({\mathbb {R}}^n;{\mathbb {R}}^n)$$.

### Comparison between $W^{\alpha ,1}$ and $BV^{\alpha }$ seminorms

In this section, we completely answer a question left open in [27,  Section 1.4]. Given $\alpha \in (0, 1)$ and an open set $\Omega \subset {\mathbb {R}}^n$, we want to study the equality cases in the inequalities

$$\begin{aligned} \Vert \nabla ^{\alpha } f\Vert _{L^{1}({\mathbb {R}}^{n};\, {\mathbb {R}}^{n})} \le \mu _{n, \alpha } [f]_{W^{\alpha , 1}({\mathbb {R}}^{n})}, \qquad |D^\alpha \chi _E|(\Omega ) \le \mu _{n,\alpha } P_\alpha (E;\Omega ), \end{aligned}$$

as long as $f\in W^{\alpha ,1}({\mathbb {R}}^n)$ and $P_\alpha (E;\Omega )<+\infty$. The key idea to the solution of this problem lies in the following simple result.

#### Lemma 2.9

Let $A\subset {\mathbb {R}}^n$ be a measurable set with ${\mathscr {L}}^{n}(A)>0$. If $F \in L^{1}(A; {\mathbb {R}}^m)$, then

$$\begin{aligned} \bigg | \int _A F(x) \, dx \, \bigg | \le \int _A | F(x) | \, dx, \end{aligned}$$

with equality if and only if $F = f \nu$ a.e. in *A* for some constant direction $\nu \in {\mathbb {S}}^{m - 1}$ and some scalar function $f \in L^{1}(A)$ with $f \ge 0$ a.e. in *A*.

#### Proof

The inequality is well known and it is obvious that it is an equality if $F = f \nu$ a.e. in *A* for some constant direction $\nu \in {\mathbb {S}}^{m-1}$ and some scalar function $f \in L^{1}(A)$ with $f \ge 0$ a.e. in *A*. So let us assume that

$$\begin{aligned} \bigg | \int _A F(x) \, dx \, \bigg | = \int _A | F(x) | \, dx. \end{aligned}$$

If $\int _A F(x) \, dx=0$, then also $\int _A | F(x) | \, dx=0$. Thus $F=0$ a.e. in *A* and there is nothing to prove. If $\int _A F(x) \, dx\ne 0$ instead, then we can write

$$\begin{aligned} \int _A |F(x)| - F(x) \cdot \nu \, dx = 0, \end{aligned}$$

with

$$\begin{aligned} \nu = \frac{\int _A F(x) \, dx}{| \int _A F(x) \, dx \,|}\in {\mathbb {S}}^{m-1}. \end{aligned}$$

Therefore, we obtain $|F(x)| = F(x) \cdot \nu$ for a.e. $x \in A$, so that $\frac{F(x)}{|F(x)|} \cdot \nu = 1$ for a.e. $x \in A$ such that $|F(x)|\ne 0$. This implies that $F = f \nu$ a.e. in *A* with $f = |F|\in L^{1}(A)$ and the conclusion follows. $\square$

As an immediate consequence of Lemma 2.9, we have the following result.

#### Corollary 2.10

Let $\alpha \in (0, 1)$. If $f \in W^{\alpha , 1}({\mathbb {R}}^{n})$, then

2.19 $$\begin{aligned} \Vert \nabla ^{\alpha } f\Vert _{L^{1}({\mathbb {R}}^{n};\, {\mathbb {R}}^{n})} \le \mu _{n, \alpha } [f]_{W^{\alpha , 1}({\mathbb {R}}^{n})}, \end{aligned}$$

with equality if and only if $f=0$ a.e. in ${\mathbb {R}}^n$.

#### Proof

Inequality (2.19) was proved in [27,  Theorem 3.18]. Note that, given $f \in L^{1}({\mathbb {R}}^{n})$, $[f]_{W^{\alpha ,1}({\mathbb {R}}^n)}=0$ if and only if $f=0$ a.e. and thus, in this case, (2.19) is trivially an equality. If (2.19) holds as an equality and *f* is not equivalent to the zero function, then

$$\begin{aligned} \int _{{\mathbb {R}}^n}\bigg (|\nabla ^\alpha f (x)|-\mu _{n,\alpha }\int _{{\mathbb {R}}^n}\frac{|f(y)-f(x)|}{|y-x|^{n+\alpha }}\,dy\bigg )\,dx=0 \end{aligned}$$

and thus

2.20 $$\begin{aligned} \bigg |\int _{{\mathbb {R}}^n}\frac{(f(y)-f(x))\cdot (y-x)}{|y-x|^{n+\alpha +1}}\,dy\,\bigg | =\int _{{\mathbb {R}}^n}\frac{|f(y)-f(x)|}{|y-x|^{n+\alpha }}\,dy \end{aligned}$$

for all $x\in U$, for some measurable set $U\subset {\mathbb {R}}^n$ such that ${\mathscr {L}}^{n}({\mathbb {R}}^n\setminus U)=0$. Now let $x\in U$ be fixed. By Lemma 2.9 (applied with $A={\mathbb {R}}^n$), (2.20) implies that the (non-identically zero) vector field

$$\begin{aligned} y\mapsto (f(y) - f(x))\,(y - x), \quad y\in {\mathbb {R}}^n, \end{aligned}$$

has constant direction for all $y\in V_x$, for some measurable set $V_x\subset {\mathbb {R}}^n$ such that ${\mathscr {L}}^{n}({\mathbb {R}}^n\setminus V_x)=0$. Thus, given $y,y'\in V_x$, the two vectors $y-x$ and $y'-x$ are linearly dependent, so that the three points *x*, *y* and $y'$ are collinear. If $n\ge 2$, then this immediately gives ${\mathscr {L}}^{n}(V_x)=0$, a contradiction, so that (2.19) must be strict. If instead $n=1$, then we know that

2.21 $$\begin{aligned} x\in U \implies y\mapsto (f(y) - f(x))\,(y - x)\ \text {has constant sign for all } y\in V_x.\qquad \end{aligned}$$

We claim that (2.21) implies that the function *f* is (equivalent to) a (non-constant) monotone function. If so, then $f\notin L^1({\mathbb {R}})$, in contrast with the fact that $f\in W^{\alpha ,1}({\mathbb {R}})$, so that (2.19) must be strict and the proof is concluded. To prove the claim, we argue as follows. Fix $x\in U$ and assume that

2.22 $$\begin{aligned} (f(y) - f(x))\,(y - x)>0 \end{aligned}$$

for all $y\in V_x$ without loss of generality. Now pick $x'\in U\cap V_x$ such that $x'>x$. Then, choosing $y=x'$ in (2.22), we get $(f(x') - f(x))\,(x' - x)>0$ and thus $f(x')>f(x)$. Similarly, if $x'\in U\cap V_x$ is such that $x'<x$, then $f(x')<f(x)$. Hence

$$\begin{aligned} {{\,\mathrm{ess\,sup}\,}}_{z<x} f(z) \le f(x) \le {{\,\mathrm{ess\,inf}\,}}_{z>x} f(z) \end{aligned}$$

for all $x\in U$ (where ${{\,\mathrm{ess\,sup}\,}}$ and ${{\,\mathrm{ess\,inf}\,}}$ refer to the *essential supremum* and the *essential infimum* respectively) and thus *f* must be equivalent to a (non-constant) non-decreasing function. $\square$

Given an open set $\Omega \subset {\mathbb {R}}^n$ and a measurable set $E\subset {\mathbb {R}}^n$, we define

$$\begin{aligned} {\tilde{P}}_{\alpha }(E; \Omega ) := \int _{\Omega } \int _{\Omega } \frac{|\chi _{E}(y) - \chi _{E}(x)|}{|y - x|^{n + \alpha }} \, dx \, dy + \int _{{\mathbb {R}}^{n} \setminus \Omega } \int _{\Omega } \frac{|\chi _{E}(y) - \chi _{E}(x)|}{|y - x|^{n + \alpha }} \, dx \, dy. \end{aligned}$$

It is obvious to see that

$$\begin{aligned} {\tilde{P}}_\alpha (E;\Omega ) \le P_\alpha (E;\Omega ) \le 2\tilde{P}_\alpha (E;\Omega ), \end{aligned}$$

where $P_\alpha$ is the fractional perimeter introduced in (1.10). Arguing as in the proof of [27,  Proposition 4.8]it is immediate to see that

2.23 $$\begin{aligned} \Vert \nabla ^{\alpha } \chi _{E}\Vert _{L^{1}(\Omega ;\, {\mathbb {R}}^{n})} \le \mu _{n, \alpha }{\tilde{P}}_{\alpha }(E;\Omega ), \end{aligned}$$

an inequality stronger than that in (1.9). In analogy with Corollary 2.10, we have the following result.

#### Corollary 2.11

Let $\alpha \in (0,1)$, $\Omega \subset {\mathbb {R}}^n$ be an open set and $E\subset {\mathbb {R}}^n$ be a measurable set such that $\tilde{P}_{\alpha }(E;\Omega )<+\infty$.

(i) If $n\ge 2$, ${\mathscr {L}}^{n}(E)>0$ and ${\mathscr {L}}^{n}({\mathbb {R}}^n\setminus E)>0$, then inequality (2.23) is strict.
(ii) If $n=1$, then (2.23) is an equality if and only if the following hold:
  - for a.e. $$x\in \Omega \cap E$$, $${\mathscr {L}}^{1}((-\infty ,x)\setminus E)=0$$ vel $${\mathscr {L}}^{1}((x,+\infty )\setminus E)=0$$;
  - for a.e. $$x\in \Omega \setminus E$$, $${\mathscr {L}}^{1}((-\infty ,x) \cap E)=0$$ vel $${\mathscr {L}}^{1}((x,+\infty ) \cap E)=0$$.

#### Proof

We prove the two statements separately.

*Proof of (i)*. Assume $n\ge 2$. Since ${\mathscr {L}}^{n}(E)>0$, for a given $x\in \Omega \setminus E$ the map

$$\begin{aligned} y\mapsto (y-x), \quad \text {for } y\in E, \end{aligned}$$

does not have constant orientation. Similarly, since ${\mathscr {L}}^{n}({\mathbb {R}}^n\setminus E)>0$, for a given $x\in \Omega \cap E$ also the map

$$\begin{aligned} y\mapsto (y-x), \quad \text {for } y\in {\mathbb {R}}^n\setminus E, \end{aligned}$$

does not have constant orientation. Hence, by Lemma 2.9, we must have

$$\begin{aligned} \bigg |\int _E\frac{y-x}{|y-x|^{n+\alpha +1}}\,dy\,\bigg | <\int _E\frac{dy}{|y-x|^{n+\alpha }}, \quad \text {for } x\in \Omega \setminus E, \end{aligned}$$

and, similarly,

$$\begin{aligned} \bigg |\int _{{\mathbb {R}}^n\setminus E}\frac{y-x}{|y-x|^{n+\alpha +1}}\,dy\,\bigg | <\int _{{\mathbb {R}}^n\setminus E}\frac{dy}{|y-x|^{n+\alpha }}, \quad \text {for } x\in \Omega \cap E. \end{aligned}$$

We thus get

$$\begin{aligned} \Vert \nabla ^{\alpha } \chi _{E}\Vert _{L^{1}(\Omega ;\, {\mathbb {R}}^{n})}&=\mu _{n,\alpha }\int _{\Omega }\bigg |\int _{{\mathbb {R}}^n}\frac{(\chi _E(y)-\chi _E(x))\cdot (y-x)}{|y-x|^{n+\alpha +1}}\,dy\,\bigg |\,dx\\&=\mu _{n,\alpha }\int _{\Omega \setminus E}\bigg |\int _E\frac{y-x}{|y-x|^{n+\alpha }}\,dy\,\bigg |\,dx\\&\quad + \mu _{n,\alpha }\int _{\Omega \cap E}\bigg |\int _{{\mathbb {R}}^n\setminus E}\frac{y-x}{|y-x|^{n+\alpha }}\,dy\,\bigg |\,dx\\&<\mu _{n,\alpha }\int _{\Omega \setminus E}\int _E\frac{dy\,dx}{|y-x|^{n+\alpha }}\\&\quad + \mu _{n,\alpha }\int _{\Omega \cap E}\int _{{\mathbb {R}}^n\setminus E}\frac{dy\,dx}{|y-x|^{n+\alpha }} =\mu _{n,\alpha }\tilde{P}_{\alpha }(E;\Omega ), \end{aligned}$$

proving (i).

*Proof of (ii)*. Assume $n=1$. We argue as in the proof of [27,  Proposition 4.12]. Let

$$\begin{aligned} f_E(y,x):=\frac{\chi _E(y)-\chi _E(x)}{|y-x|^{1+\alpha }}, \qquad \text {for } x,y\in {\mathbb {R}},\ y\ne x. \end{aligned}$$

Then we can write

$$\begin{aligned} {\tilde{P}}_\alpha (E;\Omega )&=\int _\Omega \int _{\mathbb {R}}|f_E(y,x)|\,dy\,dx\\&=\int _\Omega \bigg (\int _{-\infty }^x|f_E(y,x)|\,dy+\int _x^{+\infty }|f_E(y,x)|\,dy\bigg )\,dx \end{aligned}$$

and

$$\begin{aligned} \Vert \nabla ^\alpha \chi _E\Vert _{L^1(\Omega ;\,{\mathbb {R}})}&=\mu _{1,\alpha }\int _\Omega \bigg |\int _{\mathbb {R}}f_E(y,x)\,{{\,\mathrm{sgn}\,}}(y-x)\,dy\,\bigg |\,dx\\&=\mu _{1,\alpha }\int _\Omega \bigg |\int _{-\infty }^x f_E(y,x)\,dy-\int _x^{+\infty } f_E(y,x)\,dy\,\bigg |\,dx. \end{aligned}$$

Hence (2.23) is an equality if and only if

2.24 $$\begin{aligned}&\bigg |\int _{-\infty }^x f_E(y,x)\,dy-\int _x^{+\infty } f_E(y,x)\,dy\,\bigg |\nonumber \\&\quad = \int _{-\infty }^x|f_E(y,x)|\,dy+\int _x^{+\infty }|f_E(y,x)|\,dy \end{aligned}$$

for a.e. $x\in \Omega$. Observing that

$$\begin{aligned}&\bigg |\int _{-\infty }^x f_E(y,x)\,dy-\int _x^{+\infty } f_E(y,x)\,dy\,\bigg |\\&\quad \le \bigg |\int _{-\infty }^x f_E(y,x)\,dy\,\bigg | +\bigg |\int _x^{+\infty } f_E(y,x)\,dy\,\bigg |\\&\le \int _{-\infty }^x|f_E(y,x)|\,dy+\int _x^{+\infty }|f_E(y,x)|\,dy \end{aligned}$$

for a.e. $x\in \Omega$, we deduce that (2.23) is an equality if and only if

2.25 $$\begin{aligned}&\bigg |\int _{-\infty }^x f_E(y,x)\,dy-\int _x^{+\infty } f_E(y,x)\,dy\,\bigg |\nonumber \\&\quad = \bigg |\int _{-\infty }^x f_E(y,x)\,dy\,\bigg | +\bigg |\int _x^{+\infty } f_E(y,x)\,dy\,\bigg | \end{aligned}$$

2.26 $$\begin{aligned}&\quad = \int _{-\infty }^x|f_E(y,x)|\,dy+\int _x^{+\infty }|f_E(y,x)|\,dy \end{aligned}$$

for a.e. $x\in \Omega$. Now, on the one hand, squaring both sides of (2.25) and simplifying, we get that (2.23) is an equality if and only if

2.27 $$\begin{aligned} \bigg (\int _{-\infty }^x f_E(y,x) \,dy\bigg )\bigg (\int _x^{+\infty }f_E(y,x)\,dy\bigg )=0 \end{aligned}$$

for a.e. $x\in \Omega$. On the other hand, we can rewrite (2.26) as

$$\begin{aligned} 0&\le \int _{-\infty }^x|f_E(y,x)|\,dy -\bigg |\int _{-\infty }^x f_E(y,x)\,dy\,\bigg | \\&= \bigg |\int _x^{+\infty } f_E(y,x)\,dy\,\bigg | -\int _x^{+\infty }|f_E(y,x)|\,dy\\&\le 0 \end{aligned}$$

for a.e. $x\in \Omega$, so that we must have

$$\begin{aligned} \bigg |\int _{-\infty }^x f_E(y,x)\,dy\,\bigg | = \int _{-\infty }^x|f_E(y,x)|\,dy \end{aligned}$$

and

$$\begin{aligned} \bigg |\int _x^{+\infty } f_E(y,x)\,dy\,\bigg | =\int _x^{+\infty }|f_E(y,x)|\,dy \end{aligned}$$

for a.e. $x\in \Omega$. Hence (2.27) can be equivalently rewritten as

2.28 $$\begin{aligned} \bigg (\int _{-\infty }^x |f_E(y,x)| \,dy\bigg )\bigg (\int _x^{+\infty }|f_E(y,x)|\,dy\bigg )=0 \end{aligned}$$

for a.e. $x\in \Omega$. Thus (2.23) is an equality if and only if at least one of the two integrals in the left-hand side of (2.28) is zero, and the reader can check that (ii) readily follows. $\square$

#### Remark 2.12

(Half-lines in Corollary 2.11(ii)) In the case $n=1$, it is worth to stress that (2.23) is always an equality when the set $E\subset {\mathbb {R}}$ is (equivalent to) an half-line, i.e.,

$$\begin{aligned} \Vert \nabla ^{\alpha } \chi _{(a,+\infty )}\Vert _{L^{1}(\Omega ;\,{\mathbb {R}})} = \mu _{1, \alpha }{\tilde{P}}_{\alpha }((a,+\infty );\Omega ) \end{aligned}$$

for any $\alpha \in (0,1)$, any $a\in {\mathbb {R}}$ and any open set $\Omega \subset {\mathbb {R}}$ such that $\tilde{P}_{\alpha }((a,+\infty );\Omega )<+\infty$. However, the equality cases in (2.23) are considerably richer. Indeed, on the one side,

$$\begin{aligned} \Vert \nabla ^{\alpha } \chi _{(-5,-4)\cup (-1,+\infty )}\Vert _{L^{1}((0,1);\,{\mathbb {R}})} = \mu _{1, \alpha }{\tilde{P}}_{\alpha }((-5,-4)\cup (-1,+\infty );(0,1)) \end{aligned}$$

and, on the other side,

$$\begin{aligned} \Vert \nabla ^{\alpha } \chi _{(-5,-4)\cup (0,+\infty )}\Vert _{L^{1}((-1,1);\,{\mathbb {R}})} < \mu _{1, \alpha }{\tilde{P}}_{\alpha }((-5,-4)\cup (0,+\infty );(-1,1)) \end{aligned}$$

for any $\alpha \in (0,1)$. We leave the simple computations to the interested reader.

## Estimates and representation formulas for the fractional $\alpha$-gradient

### Integrability properties of the fractional $\alpha$-gradient

We begin with the following technical local estimate on the $W^{\alpha ,1}$-seminorm of a function in $BV_{{{\,\mathrm{loc}\,}}}$.

#### Lemma 3.1

Let $\alpha \in (0,1)$ and let $f\in BV_{{{\,\mathrm{loc}\,}}}({\mathbb {R}}^n)$. Then $f\in W^{\alpha ,1}_{{{\,\mathrm{loc}\,}}}({\mathbb {R}}^n)$ with

3.1 $$\begin{aligned}{}[f]_{W^{\alpha ,1}(B_R)} \le \frac{n\omega _n(2R)^{1-\alpha }}{1-\alpha }\,|Df|(B_{3R}) \end{aligned}$$

for all $$R>0$$.

#### Proof

Fix $R>0$ and let $f\in BV_{{{\,\mathrm{loc}\,}}}({\mathbb {R}}^n)$ be such that $f\in C^1(B_{3R})$. We can estimate

$$\begin{aligned} {[}f]_{W^{\alpha ,1}(B_R)}&=\int _{B_R}\int _{B_R}\frac{|f(y)-f(x)|}{|y-x|^{n+\alpha }}\,dy\,dx\\&=\int _{B_R}\int _{B_R\cap \Bigg \{|y-x|<2R\Bigg \}}\frac{|f(y)-f(x)|}{|y-x|^{n+\alpha }}\,dy\,dx\\&\le \int _{\{|h|<2R\}}\frac{1}{|h|^{n+\alpha }}\int _{B_R}|f(x+h)-f(x)|\,dx\,dh. \end{aligned}$$

Since

$$\begin{aligned} \int _{B_R}|f(x+h)-f(x)|\,dx&\le \int _{B_R}\int _0^1|\nabla f(x+th)\cdot h|\,dt\,dx\\&\le |h|\int _0^1\int _{B_R}|\nabla f(x+th)|\,dx\,dt\\&\le |h|\int _{B_{R+|h|}}|\nabla f(z)|\,dz \end{aligned}$$

for all $h\in {\mathbb {R}}^n$, we have

$$\begin{aligned} {[}f]_{W^{\alpha ,1}(B_R)}&\le \int _{\{ |h|<2R \} }\frac{1}{|h|^{n+\alpha -1}}\int _{B_{R+|h|}}|\nabla f(z)|\,dz\,dh\\&\le \int _{\{ |h|<2R \} }\frac{|Df|(B_{3R})}{|h|^{n+\alpha -1}}\,dh\\&=\frac{n\omega _n(2R)^{1-\alpha }}{1-\alpha }\,|Df|(B_{3R}) \end{aligned}$$

proving (3.1) for all $f\in BV_{{{\,\mathrm{loc}\,}}}({\mathbb {R}}^n)\cap C^1(B_{3R})$. Now fix $R>0$ and let $f\in BV_{{{\,\mathrm{loc}\,}}}({\mathbb {R}}^n)$. By [34,  Theorem 5.3], there exists $(f_k)_{k\in {\mathbb {N}}}\subset BV(B_{3R})\cap C^\infty (B_{3R})$ such that $|Df_k|(B_{3R})\rightarrow |Df|(B_{3R})$ and $f_k\rightarrow f$ a.e. in $B_{3R}$ as $k\rightarrow +\infty$. The conclusion thus follows by a simple application of Fatou’s Lemma. $\square$

In the following result, we collect several local integrability estimates involving the fractional $\alpha$-gradient of a function satisfying various regularity assumptions.

#### Proposition 3.2

The following statements hold.

(i) If $f\in BV({\mathbb {R}}^n)$, then $f\in BV^\alpha ({\mathbb {R}}^n)$ for all $\alpha \in (0,1)$ with $D^\alpha f=\nabla ^\alpha f{\mathscr {L}}^{n}$ and
  3.2 $$\begin{aligned} \nabla ^{\alpha } f = I_{1-\alpha }D f \quad \text {a.e. in } {\mathbb {R}}^{n}. \end{aligned}$$
  In addition, for any bounded open set $U\subset {\mathbb {R}}^n$, we have
  3.3 $$\begin{aligned} \Vert \nabla ^{\alpha } f\Vert _{L^{1}(U;\, {\mathbb {R}}^{n})} \le C_{n, \alpha , U} \, |D f|({\mathbb {R}}^{n}) \end{aligned}$$
  for all $\alpha \in (0,1)$, where $C_{n, \alpha , U}$ is as in (2.9). Finally, given an open set $A\subset {\mathbb {R}}^n$, we have
  3.4 $$\begin{aligned}&\Vert \nabla ^\alpha f\Vert _{L^1(A;\,{\mathbb {R}}^n)}\nonumber \\&\quad \le \frac{n\omega _n\,\mu _{n,\alpha }}{n+\alpha -1}\left( \frac{|Df|( {}\overline{A_r})}{1-\alpha }\,r^{1-\alpha }+\frac{n+2\alpha -1}{\alpha }\,\Vert f\Vert _{L^1({\mathbb {R}}^n)}\,r^{-\alpha } \right) \end{aligned}$$
  for all $r>0$ and $\alpha \in (0,1)$, where $A_r:=\Bigg \{x\in {\mathbb {R}}^n : {{\,\mathrm{dist}\,}}(x,A)<r\Bigg \}$. In particular, we have
  3.5 $$\begin{aligned} \Vert \nabla ^\alpha f\Vert _{L^1({\mathbb {R}}^n;\,{\mathbb {R}}^n)} \le \frac{n\omega _n\,\mu _{n,\alpha } (n + 2 \alpha - 1)^{1 - \alpha }}{\alpha (1 - \alpha )(n+\alpha -1)}\, \Vert f\Vert _{L^{1}({\mathbb {R}}^{n})}^{1 - \alpha }\, [f]_{BV({\mathbb {R}}^n)}^{\alpha }. \end{aligned}$$
(ii) If $f \in L^{\infty }({\mathbb {R}}^{n})\cap W^{\alpha , 1}_{{{\,\mathrm{loc}\,}}}({\mathbb {R}}^{n})$, then the weak fractional $\alpha$-gradient $D^{\alpha } f \in {\mathscr {M}}_{{{\,\mathrm{loc}\,}}}({\mathbb {R}}^n; {\mathbb {R}}^n)$ exists and satisfies $D^\alpha f=\nabla ^\alpha f{\mathscr {L}}^{n}$ with $\nabla ^{\alpha } f \in L^{1}_{{{\,\mathrm{loc}\,}}}({\mathbb {R}}^{n}; {\mathbb {R}}^{n})$ and
  3.6 $$\begin{aligned} \begin{aligned} \Vert \nabla ^{\alpha } f \Vert _{L^{1}(B_{R};\, {\mathbb {R}}^{n})}&\le \mu _{n, \alpha } \int _{B_{R}} \int _{{\mathbb {R}}^{n}} \frac{|f(x) - f(y)|}{|x - y|^{n + \alpha }} \, dx \, dy\\&\le \mu _{n, \alpha } \left( [f]_{W^{\alpha , 1}(B_{R})} + P_{\alpha }(B_{R})\, \Vert f\Vert _{L^{\infty }({\mathbb {R}}^{n})} \right) \end{aligned} \end{aligned}$$
  for all $R>0$ and $\alpha \in (0,1)$.
(iii) If $f \in L^{\infty }({\mathbb {R}}^{n})\cap BV_{{{\,\mathrm{loc}\,}}}({\mathbb {R}}^{n})$, then the weak fractional $\alpha$-gradient $D^{\alpha } f \in {\mathscr {M}}_\mathrm{loc}({\mathbb {R}}^n; {\mathbb {R}}^n)$ exists and satisfies $D^\alpha f=\nabla ^\alpha f{\mathscr {L}}^{n}$ with $\nabla ^{\alpha } f \in L^{1}_{{{\,\mathrm{loc}\,}}}({\mathbb {R}}^{n}; {\mathbb {R}}^{n})$ and
  3.7 $$\begin{aligned}&\Vert \nabla ^{\alpha } f \Vert _{L^{1}(B_{R};\, {\mathbb {R}}^{n})}\nonumber \\&\quad \le \mu _{n, \alpha }\left( \frac{n\omega _n(2R)^{1-\alpha }}{1-\alpha } \, |Df|(B_{3R}) + \frac{2^{\alpha + 1} (n \omega _n)^2 R^{n-\alpha }}{\alpha \,\Gamma (1-\alpha )^{-1}}\, \Vert f\Vert _{L^{\infty }({\mathbb {R}}^{n})}\right) .\qquad \qquad \end{aligned}$$
  for all $R>0$ and $\alpha \in (0,1)$.

#### Proof

We prove the three statements separately.

*Proof of (i)*. Thanks to [27,  Theorem 3.18], we just need to prove (3.3) and (3.4).

We prove (3.3). By (3.2), by Tonelli’s Theorem and by [27,  Lemma 2.4], we get

$$\begin{aligned} \int _{U} |\nabla ^{\alpha } f|\,dx \le \int _{U} I_{1-\alpha }|Df|\,dx \le C_{n,\alpha ,U}\,|D f|({\mathbb {R}}^{n}), \end{aligned}$$

where $C_{n, \alpha , U}$ is defined as in (2.9).

We now prove (3.4) in two steps.

*Proof of* (3.4), *Step 1*. Assume $f\in C^\infty _c({\mathbb {R}}^n)$ and fix $r>0$. We have

$$\begin{aligned} \int _A|\nabla ^\alpha f|\,dx&=\int _A|I_{1-\alpha }\nabla f|\,dx\\&\le \frac{\mu _{n,\alpha }}{n+\alpha -1}\left( \int _A\int _{\{|h|\le r\}}\frac{|\nabla f(x+h)|}{|h|^{n+\alpha -1}}\,dh\,dx\right. \\&\left. \quad +\int _A\Bigg |\int _{\{|h|>r\}}\frac{\nabla f(x+h)}{|h|^{n+\alpha -1}}\,dh\,\Bigg |\,dx\right) . \end{aligned}$$

We estimate the two double integrals appearing in the right-hand side separately. By Tonelli’s Theorem, we have

$$\begin{aligned} \int _A\int _{\{|h|\le r\}}\frac{|\nabla f(x+h)|}{|h|^{n+\alpha -1}}\,dh\,dx&=\int _{\{|h|\le r\}}\int _A|\nabla f(x+h)|\,dx\,\frac{dh}{|h|^{n+\alpha -1}}\\&\le \Vert \nabla f\Vert _{L^1( {}\overline{A_r};\,{\mathbb {R}}^n)}\int _{\{|h|\le r\}}\frac{dh}{|h|^{n+\alpha -1}}\\&=n\omega _n\,\frac{r^{1-\alpha }}{1-\alpha }\,\Vert \nabla f\Vert _{L^1( {}\overline{A_r};\,{\mathbb {R}}^n)}. \end{aligned}$$

Concerning the second double integral, integrating by parts we get

$$\begin{aligned} \int _{\{|h|>r\}}\frac{\nabla f(x+h)}{|h|^{n+\alpha -1}}\,dh&=(n+\alpha -1)\int _{\{|h|>r\}}\frac{h f(x+h)}{|h|^{n+\alpha +1}}\,dh\\&\quad -\int _{\{|h|=r\}}\frac{h}{|h|}\frac{f(x+h)}{|h|^{n+\alpha -1}}\,d{\mathscr {H}}^{n-1}(h) \end{aligned}$$

for all $x\in A$. Hence, we can estimate

$$\begin{aligned} \int _A\Bigg |\int _{\{|h|>r\}}\frac{\nabla f(x+h)}{|h|^{n+\alpha -1}}\,dh\,\Bigg |\,dx&\le (n+\alpha -1)\int _A\int _{\{|h|>r\}}\frac{|f(x+h)|}{|h|^{n+\alpha }}\,dh\,dx\\&\quad +\int _A\int _{\{|h|=r\}}\frac{|f(x+h)|}{|h|^{n+\alpha -1}}\,d{\mathscr {H}}^{n-1}(h)\,dx\\&\le n\omega _n\Vert f\Vert _{L^1({\mathbb {R}}^n)}\,r^{-\alpha }\left( \frac{n+\alpha -1}{\alpha } +1\right) \\&=n\omega _n\left( \frac{n+2\alpha -1}{\alpha }\right) \Vert f\Vert _{L^1({\mathbb {R}}^n)}\,r^{-\alpha }. \end{aligned}$$

Thus (3.4) follows for all $f\in C^\infty _c({\mathbb {R}}^n)$ and $r>0$.

*Proof of* (3.4), *Step 2*. Let $f\in BV({\mathbb {R}}^n)$ and fix $r>0$. Combining [34,  Theorem 5.3] with a standard cut-off approximation argument, we find $(f_k)_{k\in {\mathbb {N}}}\subset C^\infty _c({\mathbb {R}}^n)$ such that $f_k\rightarrow f$ in $L^1({\mathbb {R}}^n)$ and $|Df_k|({\mathbb {R}}^n)\rightarrow |Df|({\mathbb {R}}^n)$ as $k\rightarrow +\infty$. By Step 1, we have that

3.8 $$\begin{aligned} \Vert \nabla ^\alpha f_k\Vert _{L^1(A;\,{\mathbb {R}}^n)} \le \frac{n\omega _n\,\mu _{n,\alpha }}{n+\alpha -1}\left( \frac{|Df_k|( {}\overline{A_r})}{1-\alpha }\,r^{1-\alpha }+\frac{n+2\alpha -1}{\alpha }\,\Vert f_k\Vert _{L^1({\mathbb {R}}^n)}\,r^{-\alpha }\right) \nonumber \\ \end{aligned}$$

for all $k\in {\mathbb {N}}$. We claim that

3.9 $$\begin{aligned} (\nabla ^\alpha f_k)\,{\mathscr {L}}^{n}\rightharpoonup (\nabla ^\alpha f)\,{\mathscr {L}}^{n} \quad \text {as } k\rightarrow +\infty . \end{aligned}$$

Indeed, if $\varphi \in {{\,\mathrm{Lip}\,}}_c({\mathbb {R}}^n;{\mathbb {R}}^n)$, then $\mathrm {div}^\alpha \varphi \in L^\infty ({\mathbb {R}}^n)$ by (2.12) and thus

$$\begin{aligned} \Bigg |\int _{{\mathbb {R}}^n}\varphi \cdot \nabla ^\alpha f_k\,dx-\int _{{\mathbb {R}}^n}\varphi \cdot \nabla ^\alpha f\,dx\Bigg |&=\Bigg |\int _{{\mathbb {R}}^n}f_k\,\mathrm {div}^\alpha \varphi \,dx-\int _{{\mathbb {R}}^n}f\,\mathrm {div}^\alpha \varphi \,dx\Bigg |\\&\le \Vert \mathrm {div}^\alpha \varphi \Vert _{L^\infty ({\mathbb {R}}^n;\,{\mathbb {R}}^n)}\,\Vert f_k-f\Vert _{L^1({\mathbb {R}}^n)} \end{aligned}$$

for all $k\in {\mathbb {N}}$, so that

$$\begin{aligned} \lim _{k\rightarrow +\infty }\int _{{\mathbb {R}}^n}\varphi \cdot \nabla ^\alpha f_k\,dx =\int _{{\mathbb {R}}^n}\varphi \cdot \nabla ^\alpha f\,dx. \end{aligned}$$

Now fix $\varphi \in C_c^0({\mathbb {R}}^n;{\mathbb {R}}^n)$. Let $U\subset {\mathbb {R}}^n$ be a bounded open set such that ${{\,\mathrm{supp}\,}}\varphi \subset U$. For each $\varepsilon >0$ sufficiently small, pick $\psi _\varepsilon \in {{\,\mathrm{Lip}\,}}_c({\mathbb {R}}^n;{\mathbb {R}}^n)$ such that $\Vert \varphi -\psi _\varepsilon \Vert _{L^\infty ({\mathbb {R}}^n;\,{\mathbb {R}}^n)}<\varepsilon$ and ${{\,\mathrm{supp}\,}}\psi _\varepsilon \subset U$. Then

$$\begin{aligned}&\bigg |\int _{{\mathbb {R}}^n}\varphi \cdot \nabla ^\alpha f_k\,dx-\int _{{\mathbb {R}}^n}\varphi \cdot \nabla ^\alpha f\,dx\bigg |\\&\quad \le \Bigg |\int _{{\mathbb {R}}^n}\psi _\varepsilon \cdot \nabla ^\alpha f_k\,dx-\int _{{\mathbb {R}}^n}\psi _\varepsilon \cdot \nabla ^\alpha f\,dx\Bigg |\\&\qquad +\Vert \psi _\varepsilon -\varphi \Vert _{L^\infty ({\mathbb {R}}^n;\,{\mathbb {R}}^n)} \left( \Vert \nabla ^\alpha f_k\Vert _{L^1(U;\,{\mathbb {R}}^n)} +\Vert \nabla ^\alpha f\Vert _{L^1(U;\,{\mathbb {R}}^n)} \right) \\&\quad \le \Bigg |\int _{{\mathbb {R}}^n}\psi _\varepsilon \cdot \nabla ^\alpha f_k\,dx-\int _{{\mathbb {R}}^n}\psi _\varepsilon \cdot \nabla ^\alpha f\,dx\Bigg |\\&\qquad +\varepsilon \,C_{n,\alpha ,U} \big (|Df_k|({\mathbb {R}}^n)+|Df|({\mathbb {R}}^n)\big ), \end{aligned}$$

so that

$$\begin{aligned} \lim _{k\rightarrow +\infty }\Bigg |\int _{{\mathbb {R}}^n}\varphi \cdot \nabla ^\alpha f_k\,dx-\int _{{\mathbb {R}}^n}\varphi \cdot \nabla ^\alpha f\,dx\Bigg | \le 2\varepsilon \, C_{n,\alpha ,U}|Df|({\mathbb {R}}^n). \end{aligned}$$

Thus, (3.9) follows passing to the limit as $\varepsilon \rightarrow 0^+$. Thanks to (3.9), by [50,  Proposition 4.29]we get that

$$\begin{aligned} \Vert \nabla ^\alpha f\Vert _{L^1(A;\,{\mathbb {R}}^n)} \le \liminf _{k\rightarrow +\infty }\Vert \nabla ^\alpha f_k\Vert _{L^1(A;\,{\mathbb {R}}^n)}. \end{aligned}$$

Since

$$\begin{aligned} |Df|(U)\le \liminf _{k\rightarrow +\infty }|Df_k|(U) \end{aligned}$$

for any open set $U\subset {\mathbb {R}}^n$ by [34,  Theorem 5.2], we can estimate

$$\begin{aligned} \limsup _{k\rightarrow +\infty }|Df_k|( {}\overline{A_r})&\le \lim _{k\rightarrow +\infty }|Df_k|({\mathbb {R}}^n) -\liminf _{k\rightarrow +\infty }|Df_k|({\mathbb {R}}^n\setminus A_r)\\&\le |Df|({\mathbb {R}}^n)-|Df|({\mathbb {R}}^n\setminus A_r)\\&=|Df|( {}\overline{A_r}). \end{aligned}$$

Thus, (3.4) follows taking limits as $k\rightarrow +\infty$ in (3.8). Finally, (3.5) is easily deduced by optimising the right-hand side of (3.4) in the case $A={\mathbb {R}}^n$ with respect to $r > 0$.

*Proof of (ii)*. Assume $f \in L^{\infty }({\mathbb {R}}^{n})\cap W^{\alpha , 1}_{{{\,\mathrm{loc}\,}}}({\mathbb {R}}^{n})$. Given $R>0$, we can estimate

$$\begin{aligned} \int _{B_{R}} |\nabla ^{\alpha } f(x)| \, dx&\le \mu _{n, \alpha } \int _{B_{R}} \int _{{\mathbb {R}}^{n}} \frac{|f(x) - f(y)|}{|x - y|^{n + \alpha }} \, dx \, dy \\&= \mu _{n, \alpha } \int _{B_{R}} \int _{B_{R}} \frac{|f(x) - f(y)|}{|x - y|^{n + \alpha }} \, dx \, dy \\&\quad + \mu _{n, \alpha } \int _{B_{R}} \int _{{\mathbb {R}}^{n} \setminus B_{R}}\frac{|f(x) - f(y)|}{|x - y|^{n + \alpha }} \, dx \, dy \\&\le \mu _{n, \alpha } [f]_{W^{\alpha , 1}(B_{R})} + 2 \mu _{n, \alpha } \Vert f\Vert _{L^{\infty }({\mathbb {R}}^{n})}\\&\quad \times \int _{B_{R}} \int _{{\mathbb {R}}^{n} \setminus B_{R}} \frac{1}{|x - y|^{n + \alpha }} \, dx \, dy\\&=\mu _{n, \alpha } [f]_{W^{\alpha , 1}(B_{R})} + \mu _{n, \alpha } \Vert f\Vert _{L^{\infty }({\mathbb {R}}^{n})}P_\alpha (B_R) \end{aligned}$$

and (3.6) follows. To prove that $D^\alpha f=\nabla ^\alpha f{\mathscr {L}}^{n}$, we argue as in the proof of [27,  Proposition 4.8]. Let $\varphi \in {{\,\mathrm{Lip}\,}}_c({\mathbb {R}}^n;{\mathbb {R}}^n)$. Since $f\in L^\infty ({\mathbb {R}}^n)$, we have

$$\begin{aligned} x\mapsto |f(x)|\int _{{\mathbb {R}}^n}\frac{|\varphi (y)-\varphi (x)|}{|y-x|^{n+\alpha }}\,dy\in L^1({\mathbb {R}}^n). \end{aligned}$$

Hence, by the definition of $\mathrm {div}^\alpha$ on ${{\,\mathrm{Lip}\,}}_c$-regular vector fields (see [27,  Section 2.2]) and by Lebesgue’s Dominated Convergence Theorem, we have

$$\begin{aligned} \int _{{\mathbb {R}}^n}f\,\mathrm {div}^\alpha \varphi \,dx =\lim _{\varepsilon \rightarrow 0^+}\int _{{\mathbb {R}}^n}f(x)\int _{\{|y-x|>\varepsilon \}}\frac{(y-x)\cdot \varphi (y)}{|y-x|^{n+\alpha +1}}\,dy\,dx. \end{aligned}$$

Since

$$\begin{aligned} \begin{aligned}&\int _{{\mathbb {R}}^n}\int _{\{|y-x|>\varepsilon \}}\frac{|f(x)|\,|\varphi (y)|}{|y-x|^{n+\alpha }}\,dy\,dx\\&\quad \le \Vert f\Vert _{L^\infty ({\mathbb {R}}^n)}\int _{{\mathbb {R}}^n}|\varphi (y)|\int _{\{|y-x|>\varepsilon \}}|y-x|^{-n-\alpha }\,dx\,dy\\&\quad \le \frac{n\omega _n}{\alpha \varepsilon ^\alpha }\Vert f\Vert _{L^\infty ({\mathbb {R}}^n)}\Vert \varphi \Vert _{L^1({\mathbb {R}}^n;\,{\mathbb {R}}^n)} \end{aligned} \end{aligned}$$

for all $\varepsilon >0$, by Fubini’s Theorem we can compute

$$\begin{aligned} \begin{aligned}&\int _{{\mathbb {R}}^n}f(x)\int _{\{|y-x|>\varepsilon \}}\frac{(y-x)\cdot \varphi (y)}{|y-x|^{n+\alpha +1}}\,dy\,dx\\&\quad =-\int _{{\mathbb {R}}^n}\varphi (y)\int _{\{|x-y|>\varepsilon \}}\frac{(x-y)\,f(x)}{|x-y|^{n+\alpha +1}}\,dx\,dy\\&\quad =-\int _{{\mathbb {R}}^n}\varphi (y)\int _{\{|x-y|>\varepsilon \}}\frac{(x-y)\,(f(x)-f(y))}{|x-y|^{n+\alpha +1}}\,dx\,dy. \end{aligned} \end{aligned}$$

Since

$$\begin{aligned} |\varphi (y)|\,\Bigg |\int _{\{|x-y|>\varepsilon \}}\frac{(x-y)\,(f(x)-f(y))}{|x-y|^{n+\alpha +1}}\,dx\Bigg | \le |\varphi (y)|\int _{{\mathbb {R}}^n}\frac{|f(x)-f(y)|}{|x-y|^{n+\alpha }}\,dx \end{aligned}$$

for all $y\in {\mathbb {R}}^n$ and $\varepsilon >0$, and

$$\begin{aligned} y\mapsto \int _{{\mathbb {R}}^n}\frac{|f(x)-f(y)|}{|x-y|^{n+\alpha }}\,dx\in L^1_{{{\,\mathrm{loc}\,}}}({\mathbb {R}}^n) \end{aligned}$$

by (3.6), again by Lebesgue’s Dominated Convergence Theorem we conclude that

$$\begin{aligned} \begin{aligned} \int _{{\mathbb {R}}^n}f(x)\,\mathrm {div}^\alpha \varphi (x) \,dx&=-\lim _{\varepsilon \rightarrow 0}\int _{{\mathbb {R}}^n}\varphi (y)\int _{\{|x-y|>\varepsilon \}}\frac{(x-y)\,(f(x)-f(y))}{|x-y|^{n+\alpha +1}}\,dx\,dy\\&=-\int _{{\mathbb {R}}^n}\varphi (y)\lim _{\varepsilon \rightarrow 0}\int _{\{|x-y|>\varepsilon \}}\frac{(x-y)\,(f(x)-f(y))}{|x-y|^{n+\alpha +1}}\,dx\,dy\\&=-\int _{{\mathbb {R}}^n}\varphi (y) \cdot \nabla ^\alpha f(y)\,dy \end{aligned} \end{aligned}$$

for all $\varphi \in {{\,\mathrm{Lip}\,}}_c({\mathbb {R}}^n;{\mathbb {R}}^n)$. Thus $D^\alpha f\in {\mathscr {M}}_{{{\,\mathrm{loc}\,}}}({\mathbb {R}}^n;{\mathbb {R}}^n)$ is well defined and $D^\alpha f=\nabla ^\alpha f{\mathscr {L}}^{n}$.

*Proof of (iii)*. Assume $f\in L^{\infty }({\mathbb {R}}^{n})\cap BV_{{{\,\mathrm{loc}\,}}}({\mathbb {R}}^{n})$. By Lemma 3.1, we know that $f \in L^{\infty }({\mathbb {R}}^{n})\cap W^{\alpha , 1}_{{{\,\mathrm{loc}\,}}}({\mathbb {R}}^{n})$ for all $\alpha \in (0,1)$, so that $D^{\alpha } f \in {\mathscr {M}}_\mathrm{loc}({\mathbb {R}}^{n}; {\mathbb {R}}^{n})$ exists by (ii). Hence, inserting (3.1) in (3.6), we find

$$\begin{aligned} \Vert \nabla ^{\alpha } f \Vert _{L^{1}(B_{R};\, {\mathbb {R}}^{n})} \le \mu _{n, \alpha }\left( \frac{n\omega _n(2R)^{1-\alpha }}{1-\alpha } \, |Df|(B_{3R}) + P_{\alpha }(B_1)\,R^{n-\alpha }\, \Vert f\Vert _{L^{\infty }({\mathbb {R}}^{n})}\right) . \end{aligned}$$

Since for all $x\in B_1$ we have

$$\begin{aligned} \int _{{\mathbb {R}}^n\setminus B_1}\frac{dy}{|y-x|^{n+\alpha }} =\int _{{\mathbb {R}}^n\setminus B_1(-x)}\frac{dz}{|z|^{n+\alpha }} \le \int _{{\mathbb {R}}^n\setminus B_{1-|x|}}\frac{dz}{|z|^{n+\alpha }} =\frac{n\omega _n}{\alpha (1-|x|)^\alpha }, \end{aligned}$$

being $\Gamma$ log-convex on $(0,+\infty )$ (see [9]), we can estimate

$$\begin{aligned} \begin{aligned} P_\alpha (B_1)&=2\int _{B_1}\int _{{\mathbb {R}}^n\setminus B_1}\frac{dy\,dx}{|y-x|^{n+\alpha }} \le \frac{2n\omega _n}{\alpha }\int _{B_1}\frac{dx}{(1-|x|)^\alpha }\\&=\frac{2(n\omega _n)^2}{\alpha }\int _0^1\frac{t^{n-1}}{(1-t)^\alpha }\,dt =\frac{2(n\omega _n)^2}{\alpha }\, \frac{\Gamma (n)\,\Gamma (1-\alpha )}{\Gamma (n+1-\alpha )}\\&\le \frac{2^{\alpha +1}(n\omega _n)^2}{\alpha }\,\Gamma (1-\alpha ), \end{aligned} \end{aligned}$$

so that

$$\begin{aligned}&\Vert \nabla ^{\alpha } f \Vert _{L^{1}(B_{R};\, {\mathbb {R}}^{n})} \le \mu _{n, \alpha }\left( \frac{n\omega _n(2R)^{1-\alpha }}{1-\alpha } |Df|_{BV(B_{3R})} + \frac{2^{\alpha +1}(n\omega _n)^2R^{n-\alpha }}{\alpha \,\Gamma (1-\alpha )^{-1}}\, \Vert f\Vert _{L^{\infty }({\mathbb {R}}^{n})}\right) , \end{aligned}$$

proving (3.7). $$\square $$

Note that Proposition 3.2(i), in particular, applies to any $f\in W^{1,1}({\mathbb {R}}^n)$. In the following result, we prove that a similar result holds also for any $f\in W^{1,p}({\mathbb {R}}^n)$ with $p\in (1,+\infty )$.

#### Proposition 3.3

($W^{1,p}({\mathbb {R}}^n)\subset S^{\alpha ,p}({\mathbb {R}}^n)$ for $p\in (1,+\infty )$) Let $\alpha \in (0,1)$ and $p\in (1,+\infty )$. If $f\in W^{1,p}({\mathbb {R}}^n)$, then $f\in S^{\alpha ,p}({\mathbb {R}}^n)$ with

3.10 $$\begin{aligned} \Vert \nabla ^\alpha _w f\Vert _{L^p(A;\,{\mathbb {R}}^n)}\le & {} \frac{n\omega _n\mu _{n,\alpha }}{n+\alpha -1}\left( \frac{\Vert \nabla _w f\Vert _{L^p( {}\overline{A_r};\,{\mathbb {R}}^n)}}{1-\alpha }\,r^{1-\alpha }\right. \nonumber \\&\left. +\frac{n+2\alpha -1}{\alpha }\,\Vert f\Vert _{L^p({\mathbb {R}}^n)}\,r^{-\alpha }\right) \end{aligned}$$

for any $r>0$ and any open set $A\subset {\mathbb {R}}^n$, where $A_r:=\Bigg \{x\in {\mathbb {R}}^n : {{\,\mathrm{dist}\,}}(x,A)<r\Bigg \}$. In particular, we have

3.11 $$\begin{aligned} \Vert \nabla ^\alpha _w f\Vert _{L^p({\mathbb {R}}^n;\,{\mathbb {R}}^n)} \le \frac{(n+2\alpha -1)^{1-\alpha }}{n+\alpha -1}\frac{n\omega _n\mu _{n,\alpha }}{\alpha (1-\alpha )}\,\Vert \nabla _w f\Vert _{L^p({\mathbb {R}}^n;\,{\mathbb {R}}^n)}^\alpha \Vert f\Vert _{L^p({\mathbb {R}}^n)}^{1-\alpha }.\nonumber \\ \end{aligned}$$

In addition, if $p\in \big (1,\frac{n}{1-\alpha }\big )$ and $q=\frac{np}{n-(1-\alpha )p}$, then

3.12 $$\begin{aligned} \nabla ^\alpha _w f = I_{1-\alpha }\nabla _w f \quad \text {a.e. in } {\mathbb {R}}^n \end{aligned}$$

and $$\nabla ^\alpha _w f\in L^q({\mathbb {R}}^n;{\mathbb {R}}^n)$$.

#### Proof

We argue as in the proof of Proposition 3.2(i).

*Proof of* (3.10). The proof of (3.10) for all $f\in C^\infty _c({\mathbb {R}}^n)$ is very similar to that of (3.4) and is thus left to the reader. Now let $f\in W^{1,p}({\mathbb {R}}^n)$ and fix an open set $A\subset {\mathbb {R}}^n$ and $r>0$. Combining [34,  Theorem 4.2] with a standard cut-off approximation argument, we find $(f_k)_{k\in {\mathbb {N}}}\subset C^\infty _c({\mathbb {R}}^n)$ such that $f_k\rightarrow f$ in $W^{1,p}({\mathbb {R}}^n)$ as $k\rightarrow +\infty$. We thus have that

3.13 $$\begin{aligned} \Vert \nabla ^\alpha f_k\Vert _{L^p(A;\,{\mathbb {R}}^n)}\le & {} \frac{n\omega _n\,\mu _{n,\alpha }}{n+\alpha -1}\left( \frac{\Vert \nabla f_k\Vert _{L^p( {}\overline{A_r};\,{\mathbb {R}}^n)}}{1-\alpha }\,r^{1-\alpha }\right. \nonumber \\&\left. +\frac{n+2\alpha -1}{\alpha }\,\Vert f_k\Vert _{L^p({\mathbb {R}}^n)}\,r^{-\alpha }\right) \end{aligned}$$

for all $k\in {\mathbb {N}}$. Hence, choosing $A={\mathbb {R}}^n$, we get that the sequence $(\nabla ^\alpha f_k)_{k\in {\mathbb {N}}}$ is uniformly bounded in $L^p({\mathbb {R}}^n;{\mathbb {R}}^n)$. Up to pass to a subsequence (which we do not relabel for simplicity), there exists $g\in L^p({\mathbb {R}}^n;{\mathbb {R}}^n)$ such that $\nabla ^\alpha f_k\rightharpoonup g$ in $L^p({\mathbb {R}}^n;{\mathbb {R}}^n)$ as $k\rightarrow +\infty$. Given $\varphi \in C^\infty _c({\mathbb {R}}^n;{\mathbb {R}}^n)$, we have

$$\begin{aligned} \int _{{\mathbb {R}}^n} f_k\,\mathrm {div}^\alpha \varphi \,dx =-\int _{{\mathbb {R}}^n}\varphi \cdot \nabla ^\alpha f_k\,dx \end{aligned}$$

for all $k\in {\mathbb {N}}$. Passing to the limit as $k\rightarrow +\infty$, by Proposition 2.1 we get that

$$\begin{aligned} \int _{{\mathbb {R}}^n} f\,\mathrm {div}^\alpha \varphi \,dx =-\int _{{\mathbb {R}}^n}\varphi \cdot g\,dx \end{aligned}$$

for any $\varphi \in C^\infty _c({\mathbb {R}}^n;{\mathbb {R}}^n)$, so that $g=\nabla ^\alpha _w f$ and hence $f\in S^{\alpha ,p}({\mathbb {R}}^n)$ according to [27,  Definition 3.19]. We thus have that

$$\begin{aligned} \Vert \nabla ^\alpha _w f\Vert _{L^p(A;\,{\mathbb {R}}^n)}\le \liminf _{k\rightarrow +\infty }\Vert \nabla ^\alpha f_k\Vert _{L^p(A;\,{\mathbb {R}}^n)} \end{aligned}$$

for any open set $A\subset {\mathbb {R}}^n$, since

$$\begin{aligned} \int _{{\mathbb {R}}^n}\varphi \cdot \nabla ^\alpha _w f\,dx= & {} \lim _{k\rightarrow +\infty }\int _{{\mathbb {R}}^n}\varphi \cdot \nabla ^\alpha f_k\,dx\\\le & {} \Vert \varphi \Vert _{L^{\frac{p}{p-1}}(A;\,{\mathbb {R}}^n)}\liminf _{k\rightarrow +\infty }\Vert \nabla ^\alpha f_k\Vert _{L^p(A;\,{\mathbb {R}}^n)} \end{aligned}$$

for all $\varphi \in C^\infty _c(A;{\mathbb {R}}^n)$. Therefore, (3.10) follows by taking limits as $k\rightarrow +\infty$ in (3.13).

*Proof of* (3.11). Inequality (3.11) follows by applying (3.10) with $A={\mathbb {R}}^n$ and minimising the right-hand side with respect to $r>0$.

*Proof of* (3.12). Now assume $p\in \big (1,\frac{n}{1-\alpha }\big )$ and let $q=\frac{np}{n-(1-\alpha )p}$. Let $\varphi \in C^\infty _c({\mathbb {R}}^n;{\mathbb {R}}^n)$ be fixed. Recalling inequality (2.5), since $\varphi \in L^{\frac{q}{q-1}}({\mathbb {R}}^n;{\mathbb {R}}^n)$ we have that

$$\begin{aligned} |\varphi |\,I_{1-\alpha }|f|\in L^1({\mathbb {R}}^n), \quad |\varphi |\,I_{1-\alpha }|\nabla _w f|\in L^1({\mathbb {R}}^n). \end{aligned}$$

In particular, Fubini’s Theorem implies that

$$\begin{aligned} f\,I_{1-\alpha }\varphi \in L^1({\mathbb {R}}^n; {\mathbb {R}}^n), \quad I_{1-\alpha }\varphi \cdot \nabla _w f\in L^1({\mathbb {R}}^n). \end{aligned}$$

Since $\mathrm {div}^{\alpha } \varphi \in L^{\frac{p}{p-1}}({\mathbb {R}}^n)$ by Proposition 2.1, we also get that

$$\begin{aligned} f\,\mathrm {div}I_{1-\alpha }\varphi =f\,\mathrm {div}^\alpha \varphi \in L^1({\mathbb {R}}^n). \end{aligned}$$

Therefore, observing that $I_{1-\alpha }\varphi \in {{\,\mathrm{Lip}\,}}_b({\mathbb {R}}^n;{\mathbb {R}}^n)$ because $\nabla I_{1 - \alpha } \varphi = \nabla ^{\alpha } \varphi \in L^{\infty }({\mathbb {R}}^{n}; {\mathbb {R}}^{n^2})$ again by Proposition 2.1 and performing a standard cut-off approximation argument, we can integrate by parts and obtain

$$\begin{aligned} \int _{{\mathbb {R}}^n}\varphi \cdot I_{1-\alpha }\nabla _w f\,dx= & {} \int _{{\mathbb {R}}^n}I_{1-\alpha }\varphi \cdot \nabla _w f\,dx \\= & {} -\int _{{\mathbb {R}}^n}f\,\mathrm {div}I_{1-\alpha }\varphi \,dx =-\int _{{\mathbb {R}}^n}f\,\mathrm {div}^\alpha \varphi \,dx. \end{aligned}$$

Therefore

$$\begin{aligned} \int _{{\mathbb {R}}^n}\varphi \cdot I_{1-\alpha }\nabla _w f\,dx =-\int _{{\mathbb {R}}^n}f\,\mathrm {div}^\alpha \varphi \,dx \end{aligned}$$

for all $\varphi \in C^\infty _c({\mathbb {R}}^n;{\mathbb {R}}^n)$, proving (3.12). In particular, notice that $\nabla ^\alpha _w f\in L^q({\mathbb {R}}^n;{\mathbb {R}}^n)$ by inequality (2.5). The proof is complete. $\square$

For the case $p=+\infty$, we have the following immediate consequence of Lemma 2.4 and Proposition 2.8.

#### Corollary 3.4

($W^{1,\infty }({\mathbb {R}}^n)\subset S^{\alpha ,\infty }({\mathbb {R}}^n)$) Let $\alpha \in (0,1)$. If $f\in W^{1,\infty }({\mathbb {R}}^n)$, then $f\in S^{\alpha ,\infty }({\mathbb {R}}^n)$ with

3.14 $$\begin{aligned} \Vert \nabla ^\alpha f\Vert _{L^\infty ({\mathbb {R}}^n;\,{\mathbb {R}}^n)} \le 2^{1-\alpha }\,\frac{n\omega _n\mu _{n,\alpha }}{\alpha (1-\alpha )}\,\Vert \nabla _w f\Vert _{L^\infty ({\mathbb {R}}^n;\,{\mathbb {R}}^n)}^\alpha \Vert f\Vert _{L^\infty ({\mathbb {R}}^n)}^{1-\alpha }. \end{aligned}$$

### Two representation formulas for the $\alpha$-variation

In this section, we prove two useful representation formulas for the $\alpha$-variation.

We begin with the following weak representation formula for the fractional $\alpha$-variation of functions in $BV_{{{\,\mathrm{loc}\,}}}({\mathbb {R}}^n) \cap L^\infty ({\mathbb {R}}^n)$. Here and in the following, we denote by $f^\star$ the *precise representative* of $f\in L^1_{{{\,\mathrm{loc}\,}}}({\mathbb {R}}^n)$, see (A.1) for the definition.

#### Proposition 3.5

Let $\alpha \in (0, 1)$ and $f \in BV_{{{\,\mathrm{loc}\,}}}({\mathbb {R}}^n) \cap L^\infty ({\mathbb {R}}^n)$. Then $\nabla ^\alpha f \in L^1_{{{\,\mathrm{loc}\,}}}({\mathbb {R}}^n;{\mathbb {R}}^n)$ and

3.15 $$\begin{aligned} \int _{{\mathbb {R}}^{n}} \varphi \cdot \nabla ^{\alpha } f \, dx = \lim _{R \rightarrow + \infty } \int _{{\mathbb {R}}^{n}} \varphi \cdot I_{1 - \alpha } ( \chi _{B_{R}}^\star D f) \, dx \end{aligned}$$

for all $$\varphi \in {{\,\mathrm{Lip}\,}}_{c}({\mathbb {R}}^{n}; {\mathbb {R}}^{n})$$.

#### Proof

By Proposition 3.2(iii), we know that $\nabla ^{\alpha } f \in L^{1}_\mathrm{loc}({\mathbb {R}}^{n}; {\mathbb {R}}^{n})$ for all $\alpha \in (0,1)$. By Theorem A.1, we also know that $f\chi _{B_R}\in BV({\mathbb {R}}^n)\cap L^\infty ({\mathbb {R}}^n)$ with $D(\chi _{B_{R}} f) = \chi _{B_{R}}^\star D f + f^\star D \chi _{B_{R}}$ for all $R>0$. Now fix $\varphi \in {{\,\mathrm{Lip}\,}}_c({\mathbb {R}}^{n}; {\mathbb {R}}^{n})$ and take $R > 0$ such that ${{\,\mathrm{supp}\,}}\varphi \subset B_{R/2}$. By [27,  Theorem 3.18], we have that

$$\begin{aligned} \int _{{\mathbb {R}}^{n}} \chi _{B_{R}} f \, \mathrm {div}^{\alpha } \varphi \, dx = - \int _{{\mathbb {R}}^{n}} \varphi \cdot \nabla ^{\alpha } (\chi _{B_{R}} f) \, dx =-\int _{{\mathbb {R}}^n}\varphi \cdot I_{1-\alpha }D(\chi _{B_R}f)\,dx. \end{aligned}$$

Moreover, we can split the last integral as

3.16 $$\begin{aligned}&\int _{{\mathbb {R}}^n}\varphi \cdot I_{1-\alpha }D(\chi _{B_R}f)\,dx\nonumber \\&\quad =\int _{{\mathbb {R}}^n}\varphi \cdot I_{1-\alpha }(\chi _{B_R}^\star Df)\,dx +\int _{{\mathbb {R}}^n}\varphi \cdot I_{1-\alpha }(f^\star D\chi _{B_R})\,dx. \end{aligned}$$

For all $x\in B_{R/2}$, we can estimate

$$\begin{aligned} \Bigg |I_{1-\alpha }(f^\star D\chi _{B_R})(x)\Bigg |&=\left| \int _{\partial B_{R}} \frac{f^\star (y)}{|x - y|^{n + \alpha - 1}} \frac{y}{|y|} \, d {\mathscr {H}}^{n - 1}(y) \right| \\&= \frac{1}{R^\alpha } \left| \int _{\partial B_{1}} \frac{f^\star (R y)}{\left| y - \frac{x}{R} \right| ^{n + \alpha - 1}} \frac{y}{|y|} \, d {\mathscr {H}}^{n - 1}(y) \right| \\&\le \frac{n \omega _{n}}{R^{\alpha } \left( 1 - \frac{|x|}{R} \right) ^{n + \alpha - 1}} \, \Vert f\Vert _{L^{\infty }({\mathbb {R}}^{n})}\\&\le \frac{2^{n+\alpha -1}n\omega _n}{R^\alpha }\, \Vert f\Vert _{L^{\infty }({\mathbb {R}}^{n})} \end{aligned}$$

and so, since ${{\,\mathrm{supp}\,}}\varphi \subset B_{R/2}$, we get that

3.17 $$\begin{aligned} \Bigg |\int _{{\mathbb {R}}^n}\varphi \cdot I_{1-\alpha }(f^\star D\chi _{B_R})\,dx\,\Bigg | \le \frac{2^{n+\alpha -1}n\omega _n}{R^\alpha }\, \Vert \varphi \Vert _{L^1({\mathbb {R}}^n;{\mathbb {R}}^n)}\,\Vert f\Vert _{L^{\infty }({\mathbb {R}}^{n})}. \end{aligned}$$

Therefore, by (2.11), Lebesgue’s Dominated Convergence Theorem, (3.16) and (3.17), we get that

$$\begin{aligned} \int _{{\mathbb {R}}^{n}} f \, \mathrm {div}^{\alpha } \varphi \, dx =\lim _{R \rightarrow + \infty } \int _{{\mathbb {R}}^{n}} \chi _{B_{R}} f \, \mathrm {div}^{\alpha } \varphi \, dx =\lim _{R \rightarrow + \infty }\int _{{\mathbb {R}}^n}\varphi \cdot I_{1-\alpha }(\chi _{B_R}^\star Df)\,dx \end{aligned}$$

and the conclusion follows. $\square$

In the following result, we show that for all functions in $bv({\mathbb {R}}^n)\cap L^\infty ({\mathbb {R}}^n)$ one can actually pass to the limit as $R\rightarrow +\infty$ inside the integral in the right-hand side of (3.15).

#### Corollary 3.6

If either $f\in BV({\mathbb {R}}^n)$ or $f\in bv({\mathbb {R}}^n)\cap L^\infty ({\mathbb {R}}^n)$, then

3.18 $$\begin{aligned} \nabla ^\alpha f=I_{1-\alpha }Df \quad \text {a.e. in } {\mathbb {R}}^n. \end{aligned}$$

#### Proof

If $f\in BV({\mathbb {R}}^n)$, then (3.18) coincides with (3.2) and there is nothing to prove. So let us assume that $f\in bv({\mathbb {R}}^n)\cap L^\infty ({\mathbb {R}}^n)$. Writing $Df = \nu _{f} |D f|$ with $\nu _f\in {\mathbb {S}}^{n-1}$ |*Df*|-a.e. in ${\mathbb {R}}^n$, for all $x\in {\mathbb {R}}^n$ we have

$$\begin{aligned} \lim _{R\rightarrow +\infty }\chi _{B_R}^\star (y)\,\frac{\nu _f(y)}{|y-x|^{n+\alpha -1}}=\frac{\nu _f(y)}{|y-x|^{n+\alpha -1}} \quad \text {for } |Df|\text { -a.e. }y \ne x. \end{aligned}$$

Moreover, for all $x\in {\mathbb {R}}^n$, we have

$$\begin{aligned} \Bigg |\chi _{B_R}^\star (y)\,\frac{\nu _f(y)}{|y-x|^{n+\alpha -1}}\Bigg | \le \frac{1}{|y-x|^{n+\alpha -1}}\in L^1_y({\mathbb {R}}^n,|Df|) \quad \forall R>0, \end{aligned}$$

because $I_{1-\alpha }|Df|\in L^1_{{{\,\mathrm{loc}\,}}}({\mathbb {R}}^n)$ by [27,  Lemma 2.4]. Therefore, by Lebesgue’s Dominated Convergence Theorem (applied with respect to the finite measure |*Df*|), we get that

$$\begin{aligned} \lim _{R\rightarrow +\infty }I_{1 - \alpha } ( \chi _{B_{R}}^\star D f)(x)=(I_{1 - \alpha } D f)(x) \quad \text {for a.e. } x\in {\mathbb {R}}^n. \end{aligned}$$

Now let $\varphi \in {{\,\mathrm{Lip}\,}}_{c}({\mathbb {R}}^{n}; {\mathbb {R}}^{n})$. Since

$$\begin{aligned} |\varphi \cdot I_{1-\alpha }(\chi _{B_R}^\star Df)|\le |\varphi |\,I_{1-\alpha }|Df|\in L^1({\mathbb {R}}^n) \quad \forall R>0, \end{aligned}$$

again by Lebesgue’s Dominated Convergence Theorem we get that

3.19 $$\begin{aligned} \lim _{R\rightarrow +\infty } \int _{{\mathbb {R}}^{n}} \varphi \cdot I_{1 - \alpha } ( \chi _{B_{R}}^\star D f) \, dx =\int _{{\mathbb {R}}^{n}} \varphi \cdot I_{1 - \alpha } D f \, dx. \end{aligned}$$

The conclusion thus follows by combining (3.15) with (3.19). $\square$

### Relation between $BV^\beta$ and $BV^{\alpha , p}$ for $\beta <\alpha$ and $p > 1$

Let us recall the following result, see [27,  Lemma 3.28].

#### Lemma 3.7

Let $\alpha \in (0,1)$. The following properties hold.

(i) If $f\in BV^\alpha ({\mathbb {R}}^n)$, then $u:=I_{1-\alpha }f\in bv({\mathbb {R}}^n)$ with $Du=D^\alpha f$ in ${\mathscr {M}}({\mathbb {R}}^{n}; {\mathbb {R}}^{n})$.
(ii) If $u\in BV({\mathbb {R}}^n)$, then $f:= (-\Delta )^{\frac{1-\alpha }{2}}u\in BV^\alpha ({\mathbb {R}}^n)$ with
  $$\begin{aligned} \Vert f\Vert _{L^1({\mathbb {R}}^n)}\le c_{n,\alpha }\Vert u\Vert _{BV({\mathbb {R}}^n)} \quad \text {and}\quad D^\alpha f=D u \quad \text {in } {\mathscr {M}}({\mathbb {R}}^{n}; {\mathbb {R}}^{n}). \end{aligned}$$
  As a consequence, the operator $(-\Delta )^{\frac{1-\alpha }{2}}:BV({\mathbb {R}}^n)\rightarrow BV^\alpha ({\mathbb {R}}^n)$ is continuous.

We can thus relate functions with bounded $\alpha$-variation and functions with bounded variation via Riesz potential and the fractional Laplacian. We would like to prove a similar result between functions with bounded $\alpha$-variation and functions with bounded $\beta$-variation, for any couple of exponents $0<\beta<\alpha < 1$.

However, although the standard variation of a function $f\in L^1_{{{\,\mathrm{loc}\,}}}({\mathbb {R}}^n)$ is well defined, it is not clear whether the functional

3.20 $$\begin{aligned} \varphi \mapsto \int _{{\mathbb {R}}^{n}} f \, \mathrm {div}^{\alpha } \varphi \, dx \end{aligned}$$

is well posed for all $\varphi \in C^{\infty }_{c}({\mathbb {R}}^{n}; {\mathbb {R}}^{n})$, since $\mathrm {div}^{\alpha } \varphi$ does not have compact support. Nevertheless, thanks to Proposition 2.1, the functional in (3.20) is well defined as soon as $f\in L^p({\mathbb {R}}^n)$ for some $p\in [1,+\infty ]$. Hence, it seems natural to define the space

3.21 $$\begin{aligned} BV^{\alpha , p}({\mathbb {R}}^{n}) := \left\{ f \in L^{p}({\mathbb {R}}^{n}) : |D^{\alpha } f|({\mathbb {R}}^{n}) < \infty \right\} \end{aligned}$$

for any $\alpha \in (0, 1)$ and $p \in [1, + \infty ]$. In particular, $BV^{\alpha , 1}({\mathbb {R}}^{n}) = BV^{\alpha }({\mathbb {R}}^{n})$. Similarly, we let

$$\begin{aligned} BV^{1,p}({\mathbb {R}}^n):= \left\{ f\in L^p({\mathbb {R}}^n) : |Df|({\mathbb {R}}^n)<+\infty \right\} \end{aligned}$$

for all $p \in [1, + \infty ]$. In particular, $BV^{1, 1}({\mathbb {R}}^{n}) = BV({\mathbb {R}}^{n})$.

A further justification for the definition of these new spaces comes from the following fractional version of the Gagliardo–Nirenberg–Sobolev embedding: if $n \ge 2$ and $\alpha \in (0, 1)$, then $BV^{\alpha }({\mathbb {R}}^{n})$ is continuously embedded in $L^{p}({\mathbb {R}}^{n})$ for all $p \in \left[ 1, \frac{n}{n - \alpha } \right]$, see [27,  Theorem 3.9]. Hence, thanks to (3.21), we can equivalently write

$$\begin{aligned} BV^{\alpha }({\mathbb {R}}^{n}) \subset BV^{\alpha , p}({\mathbb {R}}^{n}) \end{aligned}$$

with continuous embedding for all $n \ge 2$, $\alpha \in (0, 1)$ and $p \in \left[ 1, \frac{n}{n - \alpha } \right]$.

Incidentally, we remark that the continuous embedding $BV^\alpha ({\mathbb {R}}^n)\subset L^{\frac{n}{n-\alpha }}({\mathbb {R}}^n)$ for $n\ge 2$ and $\alpha \in (0,1)$ can be improved using the main result of the recent work [73] (see also [74]). Indeed, if $n\ge 2$, $\alpha \in (0,1)$ and $f\in C^\infty _c({\mathbb {R}}^n)$, then, by taking $F=\nabla ^\alpha f$ in [73,  Theorem 1.1], we have that

$$\begin{aligned} \Vert f\Vert _{L^{\frac{n}{n-\alpha },1}({\mathbb {R}}^n)} \le c_{n,\alpha }\Vert I_\alpha \nabla ^\alpha f\Vert _{L^{\frac{n}{n-\alpha },1}({\mathbb {R}}^n;\,{\mathbb {R}}^n)} \le c_{n,\alpha }'\Vert \nabla ^\alpha f\Vert _{L^1({\mathbb {R}}^n;\,{\mathbb {R}}^n)} \end{aligned}$$

thanks to the boundedness of the Riesz transform $R:L^{\frac{n}{n-\alpha },1}({\mathbb {R}}^n)\rightarrow L^{\frac{n}{n-\alpha },1}({\mathbb {R}}^n;{\mathbb {R}}^n)$, where $c_{n,\alpha },c_{n,\alpha }'>0$ are two constants depending only on *n* and $\alpha$, and $L^{\frac{n}{n-\alpha },1}({\mathbb {R}}^n)$ is the Lorentz space of exponents $\frac{n}{n-\alpha },1$ (we refer to [39, 40] for an account on Lorentz spaces and on the properties of Riesz transform). Thus, recalling [27,  Theorem 3.8], we readily deduce the continuous embedding $BV^\alpha ({\mathbb {R}}^n)\subset L^{\frac{n}{n-\alpha },1}({\mathbb {R}}^n)$ for $n\ge 2$ and $\alpha \in (0,1)$ by [39,  Exercise 1.1.1(b)] and Fatou’s Lemma. This suggests that the spaces defined in (3.21) may be further enlarged by considering functions belonging to some Lorentz space, but we do not need this level of generality here.

In the case $n=1$, the space $BV^\alpha ({\mathbb {R}})$ does not embed in $L^{\frac{1}{1-\alpha }}({\mathbb {R}})$ with continuity, see [27,  Remark 3.10]. However, somehow completing the picture provided by [73], we can prove that the space $BV^\alpha ({\mathbb {R}})$ continuously embeds in the Lorentz space $L^{\frac{1}{1 - \alpha }, \infty }({\mathbb {R}})$. Although this result is truly interesting only for $n=1$, we prove it below in all dimensions for the sake of completeness.

#### Theorem 3.8

(Weak Gagliardo–Nirenberg–Sobolev inequality). Let $\alpha \in (0,1)$. There exists a constant $c_{n,\alpha }>0$ such that

3.22 $$\begin{aligned} \Vert f\Vert _{L^{\frac{n}{n-\alpha },\infty }({\mathbb {R}}^n)} \le c_{n,\alpha }|D^\alpha f|({\mathbb {R}}^n) \end{aligned}$$

for all $f\in BV^\alpha ({\mathbb {R}}^n)$. As a consequence, $BV^{\alpha }({\mathbb {R}}^{n})$ is continuously embedded in $L^q({\mathbb {R}}^n)$ for any $q\in [1,\frac{n}{n-\alpha })$.

#### Proof

Let $f\in C^\infty _c({\mathbb {R}}^n)$. By [72,  Theorem 3.5] (see also [27,  Section 3.6]), we have

$$\begin{aligned} f(x) =-\mathrm {div}^{-\alpha }\nabla ^\alpha f(x) =-\mu _{n,-\alpha }\int _{{\mathbb {R}}^n}\frac{(y-x)\cdot \nabla ^\alpha f(y)}{|y-x|^{n+1-\alpha }}\,dy, \quad x\in {\mathbb {R}}^n, \end{aligned}$$

so that

$$\begin{aligned} |f(x)| \le \mu _{n,-\alpha }\int _{{\mathbb {R}}^n}\frac{|\nabla ^\alpha f(y)|}{|y-x|^{n-\alpha }}\,dy =\frac{\mu _{n,-\alpha }}{\mu _{n,1-\alpha }}\,(n-\alpha )\, I_\alpha |\nabla ^\alpha f|(x), \quad x\in {\mathbb {R}}^n. \end{aligned}$$

Since $I_\alpha :L^1({\mathbb {R}}^n)\rightarrow L^{\frac{n}{n-\alpha },\infty }({\mathbb {R}}^n)$ is a continuous operator by Hardy–Littlewood–Sobolev inequality (see [76,  Theorem 1, Chapter V] or [39,  Theorem 1.2.3]), we can estimate

$$\begin{aligned} \Vert f\Vert _{L^{\frac{n}{n-\alpha },\infty }({\mathbb {R}}^n)}&\le \frac{n\,\mu _{n,-\alpha }}{\mu _{n,1-\alpha }}\Vert I_\alpha |\nabla ^\alpha f|\Vert _{L^{\frac{n}{n-\alpha },\infty }({\mathbb {R}}^n)} \\&\le c_{n,\alpha }\Vert |\nabla ^\alpha f|\Vert _{L^1({\mathbb {R}}^n)} =c_{n,\alpha }\,|D^\alpha f|({\mathbb {R}}^n), \end{aligned}$$

where $c_{n,\alpha }>0$ is a constant depending only on *n* and $\alpha$. Thus, inequality (3.22) follows for all $f\in C^\infty _c({\mathbb {R}}^n)$. Now let $f\in BV^\alpha ({\mathbb {R}}^n)$. By [27,  Theorem 3.8], there exists $(f_k)_{k\in {\mathbb {N}}}\subset C^\infty _c({\mathbb {R}}^n)$ such that $f_k\rightarrow f$ a.e. in ${\mathbb {R}}^n$ and $|D^\alpha f_k|({\mathbb {R}}^n)\rightarrow |D^\alpha f|({\mathbb {R}}^n)$ as $k\rightarrow +\infty$. By [39,  Exercise 1.1.1(b)] and Fatou’s Lemma, we thus get

$$\begin{aligned} \Vert f\Vert _{L^{\frac{n}{n-\alpha },\infty }({\mathbb {R}}^n)}&\le \liminf _{k\rightarrow +\infty }\Vert f_k\Vert _{L^{\frac{n}{n-\alpha },\infty }({\mathbb {R}}^n)}\\&\le c_{n,\alpha }\lim _{k\rightarrow +\infty }|D^\alpha f_k|({\mathbb {R}}^n) =c_{n,\alpha }|D^\alpha f|({\mathbb {R}}^n) \end{aligned}$$

and so (3.22) readily follows. Finally, thanks to [39,  Proposition 1.1.14], we obtain the continuous embedding of $BV^{\alpha }({\mathbb {R}}^{n})$ in $L^{q}({\mathbb {R}}^{n})$ for all $q \in [1, \frac{n}{n - \alpha } )$. $\square$

#### Remark 3.9

*(The embedding* $BV^{\alpha }({\mathbb {R}})\subset L^{\frac{1}{1 - \alpha }, \infty }({\mathbb {R}})$ *is sharp)* Let $\alpha \in (0,1)$. The continuous embedding $BV^{\alpha }({\mathbb {R}})\subset L^{\frac{1}{1 - \alpha }, \infty }({\mathbb {R}})$ is sharp at the level of Lorentz spaces, in the sense that $BV^\alpha ({\mathbb {R}}^n)\setminus L^{\frac{1}{1 - \alpha }, q}({\mathbb {R}})\ne \varnothing$ for any $q \in [1, + \infty )$. Indeed, if we let

$$\begin{aligned} f_{\alpha }(x) = |x - 1|^{\alpha - 1} {{\,\mathrm{sgn}\,}}(x - 1) - |x|^{\alpha - 1} {{\,\mathrm{sgn}\,}}(x), \qquad x\in {\mathbb {R}}\setminus \{0,1\}, \end{aligned}$$

then $f_{\alpha } \in BV^{\alpha }({\mathbb {R}})$ by [27,  Theorem 3.26], and it is not difficult to prove that $f_{\alpha } \in L^{\frac{1}{1 - \alpha }, \infty }({\mathbb {R}})$. However, we can find a constant $c_{\alpha } > 0$ such that

$$\begin{aligned} |f_{\alpha }(x)| \ge c_{\alpha } |x|^{\alpha - 1} \chi _{\left( - \frac{1}{4}, \frac{1}{4} \right) }(x) =: g_{\alpha }(x), \qquad x\in {\mathbb {R}}\setminus \{0,1\}, \end{aligned}$$

so that $d_{f_{\alpha }} \ge d_{g_{\alpha }}$, where $d_{f_{\alpha }}$ and $d_{g_{\alpha }}$ are the *distribution functions* of $f_{\alpha }$ and $g_{\alpha }$. A simple calculation shows that

$$\begin{aligned} d_{g_{\alpha }}(s) = {\left\{ \begin{array}{ll} \dfrac{1}{2} &{} \text {if} \ 0 < s \le c_{\alpha } 4^{1 - \alpha }\\ 2 \left( \dfrac{c_{\alpha }}{t} \right) ^{\frac{1}{1 - \alpha }} &{} \text {if} \ \ s> c_{\alpha } 4^{1 - \alpha }, \end{array}\right. } \end{aligned}$$

so that, by [39,  Proposition 1.4.9], we obtain

$$\begin{aligned} \Vert f_{\alpha }\Vert _{L^{\frac{1}{1 - \alpha }, q}({\mathbb {R}})}^{q}&\ge \Vert g_{\alpha }\Vert _{L^{\frac{1}{1 - \alpha }, q}({\mathbb {R}})}^{q} = \frac{1}{1 - \alpha } \int _{0}^{+\infty } \left[ d_{g_{\alpha }}(s) \right] ^{q(1 - \alpha )} s^{q - 1} \, ds\\&\ge \frac{2^{q(1 - \alpha )}}{1 - \alpha } \int _{c_{\alpha } 4^{1 - \alpha }}^{+\infty } s^{-q} s^{q - 1} \, ds = + \infty \end{aligned}$$

and thus $f_{\alpha } \notin L^{\frac{1}{1-\alpha },q}({\mathbb {R}})$ for any $q\in [1,+\infty )$.

We collect the above continuous embeddings in the following statement.

#### Corollary 3.10

(The embedding $BV^{\alpha }\subset BV^{\alpha , p}$) Let $\alpha \in (0, 1)$ and $p \in \left[ 1, \frac{n}{n - \alpha }\right)$. We have $BV^{\alpha }({\mathbb {R}}^{n}) \subset BV^{\alpha , p}({\mathbb {R}}^{n})$ with continuous embedding. In addition, if $n \ge 2$, then also $BV^{\alpha }({\mathbb {R}}^{n}) \subset BV^{\alpha , \frac{n}{n - \alpha }}({\mathbb {R}}^{n})$ with continuous embedding.

With Corollary 3.10 at hands, we are finally ready to investigate the relation between $\alpha$-variation and $\beta$-variation for $0< \beta< \alpha < 1$.

#### Lemma 3.11

Let $0<\beta<\alpha <1$. The following hold.

(i) If $f\in BV^\beta ({\mathbb {R}}^n)$, then $u:=I_{\alpha -\beta }f\in BV^{\alpha , p}({\mathbb {R}}^n)$ for any $p \in \left( \frac{n}{n - \alpha + \beta }, \frac{n}{n - \alpha } \right)$ (including $p = \frac{n}{n - \alpha }$ if $n \ge 2$), with $D^\alpha u=D^\beta f$ in ${\mathscr {M}}({\mathbb {R}}^n;{\mathbb {R}}^n)$.
(ii) If $u\in BV^\alpha ({\mathbb {R}}^n)$, then $f:=(-\Delta )^{\frac{\alpha -\beta }{2}}u\in BV^\beta ({\mathbb {R}}^n)$ with
  $$\begin{aligned} \Vert f\Vert _{L^1({\mathbb {R}}^n)}\le c_{n,\alpha ,\beta }\,\Vert u\Vert _{BV^\alpha ({\mathbb {R}}^n)} \quad \text {and}\quad D^\beta f=D^\alpha u \quad \text {in}\ {\mathscr {M}}({\mathbb {R}}^n;{\mathbb {R}}^n). \end{aligned}$$

As a consequence, the operator $(-\Delta )^{\frac{\alpha -\beta }{2}}:BV^\alpha ({\mathbb {R}}^n)\rightarrow BV^\beta ({\mathbb {R}}^n)$ is continuous.

#### Proof

We begin with the following observation. Let $\varphi \in C^\infty _c({\mathbb {R}}^n;{\mathbb {R}}^n)$ and let $U\subset {\mathbb {R}}^n$ be a bounded open set such that ${{\,\mathrm{supp}\,}}\varphi \subset U$. By Proposition 2.1 and the *semigroup property* (2.4) of the Riesz potential, we can write

$$\begin{aligned} \mathrm {div}^\beta \varphi =I_{1-\beta }\mathrm {div}\varphi =I_{\alpha -\beta }I_{1-\alpha }\mathrm {div}\varphi =I_{\alpha -\beta }\mathrm {div}^\alpha \varphi . \end{aligned}$$

Similarly, we also have

$$\begin{aligned} I_{\alpha -\beta }|\mathrm {div}^\alpha \varphi | =I_{\alpha -\beta }|I_{1-\alpha }\mathrm {div}\varphi | \le I_{\alpha -\beta }I_{1-\alpha }|\mathrm {div}\varphi | =I_{1-\beta }|\mathrm {div}\varphi |, \end{aligned}$$

so that $I_{\alpha -\beta }|\mathrm {div}^\alpha \varphi |\in L^\infty ({\mathbb {R}}^n)$ with

$$\begin{aligned} \Vert I_{\alpha -\beta }|\mathrm {div}^\alpha \varphi |\Vert _{L^\infty ({\mathbb {R}}^n)} \le \Vert I_{1-\beta }|\mathrm {div}\varphi |\Vert _{L^\infty ({\mathbb {R}}^n)} \le C_{n,\beta ,U}\Vert \mathrm {div}\varphi \Vert _{L^\infty ({\mathbb {R}}^n)} \end{aligned}$$

by [27,  Lemma 2.4] We now prove the two statements separately.

*Proof of (i)*. Let $f\in BV^\beta ({\mathbb {R}}^n)$ and $\varphi \in C^\infty _c({\mathbb {R}}^n;{\mathbb {R}}^n)$. Thanks to Corollary 3.10, if $n \ge 2$, then $f \in BV^{\beta , q}({\mathbb {R}}^{n})$ for any $q \in [1, \frac{n}{n - \beta }]$ and so $I_{\alpha - \beta } f \in L^{p}({\mathbb {R}}^{n})$ for any $p \in \left( \frac{n}{n - \alpha + \beta }, \frac{n}{n - \alpha } \right]$ by (2.5). If instead $n = 1$, then $f \in BV^{\beta , q}({\mathbb {R}})$ for any $q \in [1, \frac{1}{1 - \beta })$ and so $I_{\alpha - \beta } f \in L^{p}({\mathbb {R}})$ for any $p \in \left( \frac{1}{1 - \alpha + \beta }, \frac{1}{1 - \alpha } \right)$. Since $f\in L^1({\mathbb {R}}^n)$ and $I_{\alpha -\beta }|\mathrm {div}^\alpha \varphi |\in L^\infty ({\mathbb {R}}^n)$, by Fubini’s Theorem we have

3.23 $$\begin{aligned} \int _{{\mathbb {R}}^n}f\,\mathrm {div}^\beta \varphi \,dx =\int _{{\mathbb {R}}^n}f\,I_{\alpha -\beta }\mathrm {div}^\alpha \varphi \,dx =\int _{{\mathbb {R}}^n}u\,\mathrm {div}^\alpha \varphi \,dx, \end{aligned}$$

proving that $u:=I_{\alpha -\beta }f\in BV^{\alpha , p}({\mathbb {R}}^n)$ for any $p \in \left( \frac{n}{n - \alpha + \beta }, \frac{n}{n - \alpha } \right)$ (including $p = \frac{n}{n - \alpha }$ if $n \ge 2$), with $D^\alpha u=D^\beta f$ in ${\mathscr {M}}({\mathbb {R}}^n;{\mathbb {R}}^n)$.

*Proof of (ii)*. Let $u\in BV^\alpha ({\mathbb {R}}^n)$. By [27,  Theorem 3.32] we know that $u\in W^{\alpha -\beta ,1}({\mathbb {R}}^n)$, so that $f:=(-\Delta )^{\frac{\alpha -\beta }{2}}u\in L^1({\mathbb {R}}^n)$ with $\Vert f\Vert _{L^1({\mathbb {R}}^n)}\le c_{n,\alpha ,\beta }\,\Vert u\Vert _{BV^\alpha ({\mathbb {R}}^n)}$, see [27,  Section 3.10] Then, arguing as before, for any $\varphi \in C^\infty _c({\mathbb {R}}^n;{\mathbb {R}}^n)$ we get (3.23), since we have $I_{\alpha -\beta }f =u$ in $L^1({\mathbb {R}}^n)$ (see [27,  Section 3.10]). The proof is complete. $\square$

### The inclusion $BV^\alpha \subset W^{\beta ,1}$ for $\beta <\alpha$: a representation formula

In [27,  Theorem 3.32], we proved that the inclusion $BV^\alpha \subset W^{\beta ,1}$ is continuous for $\beta <\alpha$. In the following result we prove a useful representation formula for the fractional $\beta$-gradient of any $f\in BV^\alpha ({\mathbb {R}}^n)$, extending the formula obtained in Corollary 3.6.

#### Proposition 3.12

Let $\alpha \in (0,1)$. If $f\in BV^\alpha ({\mathbb {R}}^n)$, then $f\in W^{\beta ,1}({\mathbb {R}}^n)$ for all $\beta \in (0,\alpha )$ with

3.24 $$\begin{aligned} \nabla ^\beta f=I_{\alpha -\beta }D^\alpha f \quad \text {a.e. in } {\mathbb {R}}^n. \end{aligned}$$

In addition, for any bounded open set $U\subset {\mathbb {R}}^n$, we have

3.25 $$\begin{aligned} \Vert \nabla ^\beta f\Vert _{L^1(U;\,{\mathbb {R}}^n)} \le C_{n,(1 - \alpha +\beta ),U}\,|D^\alpha f|({\mathbb {R}}^n) \end{aligned}$$

for all $\beta \in (0,\alpha )$, where $C_{n,\alpha ,U}$ is as in (2.9). Finally, given an open set $A\subset {\mathbb {R}}^n$, we have

3.26 $$\begin{aligned} \Vert \nabla ^\beta f\Vert _{L^1(A;\,{\mathbb {R}}^n)}\le & {} \frac{\mu _{n,1 + \beta - \alpha }}{n+\beta -\alpha }\left( \frac{\omega _{n, 1}|D^\alpha f|( {}\overline{A_r})}{\alpha -\beta }\,r^{\alpha -\beta }\right. \nonumber \\&\left. +\frac{\omega _{n,\alpha }(n + 2 \beta -\alpha )}{\beta }\,\Vert f\Vert _{L^1({\mathbb {R}}^n)}\,r^{-\beta }\right) \end{aligned}$$

for all $r>0$ and all $\beta \in (0,\alpha )$, where $\omega _{n,\alpha }:=\Vert \nabla ^\alpha \chi _{B_1}\Vert _{L^{1}({\mathbb {R}}^n; {\mathbb {R}}^{n})}$, $\omega _{n, 1} := |D \chi _{B_{1}}|({\mathbb {R}}^{n}) = n \omega _{n}$, and, as above, $A_r:=\Bigg \{x\in {\mathbb {R}}^n : {{\,\mathrm{dist}\,}}(x,A)<r\Bigg \}$. In particular, we have

3.27 $$\begin{aligned}&\Vert \nabla ^\beta f\Vert _{L^1({\mathbb {R}}^{n};\,{\mathbb {R}}^n)}\nonumber \\&\quad \le \frac{\alpha \mu _{n, 1 + \beta - \alpha } \omega _{n, 1}^{\frac{\beta }{\alpha }} \omega _{n, \alpha }^{1 - \frac{\beta }{\alpha }} (n + 2 \beta - \alpha )^{1 - \frac{\beta }{\alpha }}}{\beta (n + \beta - \alpha )(\alpha - \beta )} \, \Vert f\Vert _{L^{1}({\mathbb {R}}^{n})}^{1 - \frac{\beta }{\alpha }}\, |D^{\alpha }f|({\mathbb {R}}^{n})^{\frac{\beta }{\alpha }}. \end{aligned}$$

#### Proof

Fix $\beta \in (0,\alpha )$. By [27,  Theorem 3.32] we already know that $f\in W^{\beta ,1}({\mathbb {R}}^n)$, with $D^\beta f=\nabla ^\beta f{\mathscr {L}}^{n}$ according to [27,  Theorem 3.18]. We thus just need to prove (3.24), (3.25) and (3.26).

We prove (3.24). Let $\varphi \in C^\infty _c({\mathbb {R}}^n;{\mathbb {R}}^n)$. Note that $I_{\alpha -\beta }\varphi \in {{\,\mathrm{Lip}\,}}_b({\mathbb {R}}^n;{\mathbb {R}}^n)$ is such that $\mathrm {div}I_{\alpha -\beta }\varphi =I_{\alpha -\beta }\mathrm {div}\varphi$, so that

$$\begin{aligned} I_{1-\alpha }\mathrm {div}I_{\alpha -\beta }\varphi =I_{1-\alpha }I_{\alpha -\beta }\mathrm {div}\varphi =I_{1-\beta }\mathrm {div}\varphi =\mathrm {div}^\beta \varphi \end{aligned}$$

by the *semigroup property* (2.4) of the Riesz potential. Moreover, in a similar way, we have

$$\begin{aligned} I_{1-\alpha }|\mathrm {div}I_{\alpha -\beta }\varphi | =I_{1-\alpha }|I_{\alpha -\beta }\mathrm {div}\varphi | \le I_{1-\alpha }I_{\alpha -\beta }|\mathrm {div}\varphi | =I_{1-\beta }|\mathrm {div}\varphi | \in L^1_{{{\,\mathrm{loc}\,}}}({\mathbb {R}}^n). \end{aligned}$$

By Lemma 2.2, we thus have that $\mathrm {div}^\alpha I_{\alpha -\beta }\varphi =\mathrm {div}^\beta \varphi$. Consequently, by Proposition 2.7, we get

$$\begin{aligned} \int _{{\mathbb {R}}^n}f\,\mathrm {div}^\beta \varphi \,dx =\int _{{\mathbb {R}}^n}f\,\mathrm {div}^\alpha I_{\alpha -\beta }\varphi \,dx =-\int _{{\mathbb {R}}^n}I_{\alpha -\beta }\varphi \cdot dD^\alpha f. \end{aligned}$$

Since $|D^\alpha f|({\mathbb {R}}^n)<+\infty$, we have $I_{\alpha -\beta }|D^\alpha f|\in L^1_{{{\,\mathrm{loc}\,}}}({\mathbb {R}}^n)$ and thus, by Fubini’s Theorem, we get that

$$\begin{aligned} \int _{{\mathbb {R}}^n}I_{\alpha -\beta }\varphi \cdot dD^\alpha f =\int _{{\mathbb {R}}^n}\varphi \cdot I_{\alpha -\beta }D^\alpha f\,dx. \end{aligned}$$

We conclude that

$$\begin{aligned} \int _{{\mathbb {R}}^n}f\,\mathrm {div}^\beta \varphi \,dx =-\int _{{\mathbb {R}}^n}\varphi \cdot I_{\alpha -\beta }D^\alpha f\,dx \end{aligned}$$

for any $\varphi \in C^\infty _c({\mathbb {R}}^n;{\mathbb {R}}^n)$, proving (3.24).

We prove (3.25). By (3.24), by Tonelli’s Theorem and by [27,  Lemma 2.4], we get

$$\begin{aligned} \int _U |\nabla ^{\beta } f|\,dx \le \int _U I_{\alpha -\beta }|D^\alpha f|\,dx \le C_{n,(1-\alpha +\beta ),U}|D^\alpha f|({\mathbb {R}}^n) \end{aligned}$$

where $C_{n,\alpha , U}$ is as in (2.9).

We now prove (3.26) in two steps. We argue as in the proof of (3.4).

*Proof of* (3.26), *Step 1*. Assume $f\in C^\infty _c({\mathbb {R}}^n)$ and fix $r>0$. We have

$$\begin{aligned} \int _A|\nabla ^\beta f| dx&=\int _A|I_{\alpha -\beta }\nabla ^\alpha f|\,dx\\&\le \frac{\mu _{n,1+\beta -\alpha }}{n+\beta -\alpha }\left( \int _A\int _{\{|h|<r\}}\frac{|\nabla ^\alpha f(x+h)|}{|h|^{n+\beta -\alpha }} dh dx \right. \\&\quad \left. +\int _A\Bigg |\int _{\{|h|\ge r\}}\frac{\nabla ^\alpha f(x+h)}{|h|^{n+\beta -\alpha }}\,dh \Bigg | dx\right) . \end{aligned}$$

We estimate the two double integrals appearing in the right-hand side separately. By Tonelli’s Theorem, we have

$$\begin{aligned} \int _A\int _{\{|h|<r\}}\frac{|\nabla ^\alpha f(x+h)|}{|h|^{n+\beta -\alpha }}\,dh\,dx&=\int _{\{|h|<r\}}\int _A|\nabla ^\alpha f(x+h)|\,dx\,\frac{dh}{|h|^{n+\beta -\alpha }}\\&\le |D^\alpha f|(A_r)\int _{\{|h|<r\}}\frac{dh}{|h|^{n+\beta -\alpha }}\\&=\frac{n\omega _n\,|D^\alpha f|(A_r)}{\alpha -\beta }\,r^{\alpha -\beta }. \end{aligned}$$

Concerning the second double integral, we apply [1,  Lemma 3.1.1(c)] to each component of the measure $D^\alpha f\in {\mathscr {M}}({\mathbb {R}}^n;{\mathbb {R}}^n)$ and get

$$\begin{aligned} \int _{\{|h|\ge r\}}\frac{\nabla ^\alpha f(x+h)}{|h|^{n+\beta -\alpha }}\,dh&=(n+\beta -\alpha )\int _r^{+\infty }\frac{D^\alpha f(B_\varrho (x))}{\varrho ^{n+\beta -\alpha +1}}\,d\varrho -\frac{D^\alpha f(B_r(x))}{r^{n+\beta -\alpha }} \end{aligned}$$

for all $x\in A$. Since

$$\begin{aligned} D^\alpha f(B_\varrho (x))&=\int _{{\mathbb {R}}^n}\chi _{B_\varrho }(y)\,\nabla ^\alpha f(x+y)\,dy\\&=-\int _{{\mathbb {R}}^n} f(x+y)\,\nabla ^\alpha \chi _{B_\varrho }(y)\,dy\\&=-\varrho ^{n-\alpha }\int _{{\mathbb {R}}^n}f(x+\varrho y)\,\nabla ^\alpha \chi _{B_1}(y)\,dy, \end{aligned}$$

we can compute

$$\begin{aligned}&(n+\beta -\alpha )\int _r^{+\infty }\frac{D^\alpha f(B_\varrho (x))}{\varrho ^{n+\beta -\alpha +1}}\,d\varrho -\frac{D^\alpha f(B_r(x))}{r^{n+\beta -\alpha }}\\&\quad = - (n+\beta -\alpha )\int _r^{+\infty }\frac{1}{\varrho ^{\beta +1}} \int _{{\mathbb {R}}^n}f(x+\varrho y)\,\nabla ^\alpha \chi _{B_1}(y)\,dy \,d\varrho \\&\qquad + \frac{1}{r^{\beta }} \int _{{\mathbb {R}}^n}f(x+r y)\,\nabla ^\alpha \chi _{B_1}(y)\,dy \\&\quad =\int _{{\mathbb {R}}^{n}} \left( \frac{f(x + r y)}{r^{\beta }} - (n+\beta -\alpha ) \int _{r}^{+ \infty } \frac{f(x + \varrho y)}{\varrho ^{\beta +1}} \, d \varrho \right) \nabla ^{\alpha } \chi _{B_{1}}(y) \, dy \end{aligned}$$

for all $x\in A$. Hence, we have

$$\begin{aligned}&\int _{A}\bigg |\int _{\{|h|>r\}}\frac{\nabla ^{\alpha } f(x+h)}{|h|^{n+\beta -\alpha }} \, dh\, \bigg | \,dx \le \int _{{\mathbb {R}}^{n}}\bigg |\int _{\{|h|>r\}}\frac{\nabla ^{\alpha } f(x+h)}{|h|^{n+\beta -\alpha }}\,dh\,\bigg |\,dx \\&\quad \le \int _{{\mathbb {R}}^{n}} \int _{{\mathbb {R}}^{n}} \frac{|f(x + r y)|}{r^{\beta }} \, |\nabla ^{\alpha } \chi _{B_{1}}(y)| \, dx \, dy \\&\qquad + (n+\beta -\alpha ) \int _{{\mathbb {R}}^{n}} \int _{r}^{+ \infty } \int _{{\mathbb {R}}^{n}} \frac{|f(x + \varrho y)|}{\varrho ^{\beta + 1}}\, |\nabla ^{\alpha } \chi _{B_{1}}(y)| \, dx \, d \varrho \, dy\\&\quad =\frac{\omega _{n,\alpha }(n + 2 \beta -\alpha )}{\beta }\,\Vert f\Vert _{L^1({\mathbb {R}}^n)}\,r^{-\beta }. \end{aligned}$$

Thus (3.4) follows for all $f\in C^\infty _c({\mathbb {R}}^n)$ and $r>0$.

*Proof of* (3.4), *Step 2*. Let $f\in BV^\alpha ({\mathbb {R}}^n)$ and fix $r>0$. By [27,  Theorem 3.8], we find $(f_k)_{k\in {\mathbb {N}}}\subset C^\infty _c({\mathbb {R}}^n)$ such that $f_k\rightarrow f$ in $L^1({\mathbb {R}}^n)$ and $|D^\alpha f_k|({\mathbb {R}}^n)\rightarrow |D^\alpha f|({\mathbb {R}}^n)$ as $k\rightarrow +\infty$. By Step 1, we have that

3.28 $$\begin{aligned} \Vert \nabla ^\beta f_k\Vert _{L^1(A;\,{\mathbb {R}}^n)}\le & {} \frac{\mu _{n,1+\beta -\alpha }}{n+\beta -\alpha }\left( \frac{n\omega _n|D^\alpha f_k|( {}\overline{A_r})}{\alpha -\beta }\,r^{\alpha -\beta }\right. \nonumber \\&\left. +\frac{\omega _{n,\alpha }(n + 2 \beta -\alpha )}{\beta }\,\Vert f_k\Vert _{L^1({\mathbb {R}}^n)}\,r^{-\beta }\right) \end{aligned}$$

for all $k\in {\mathbb {N}}$. We have that

3.29 $$\begin{aligned} (\nabla ^\beta f_k)\,{\mathscr {L}}^{n}\rightharpoonup (\nabla ^\beta f)\,{\mathscr {L}}^{n} \quad \text {as } k\rightarrow +\infty . \end{aligned}$$

This can be proved arguing as in the proof of (3.9) using (3.25). At this point the proof goes like that of Proposition 3.2(i) and we thus leave the details to the reader. $\square$

## Asymptotic behavior of fractional $\alpha$-variation as $\alpha \rightarrow 1^-$

### Convergence of $\nabla ^\alpha$ and $\mathrm {div}^\alpha$ as $\alpha \rightarrow 1^-$

We begin with the following simple result about the asymptotic behavior of the constant $\mu _{n,\alpha }$ as $\alpha \rightarrow 1^-$.

#### Lemma 4.1

Let $n\in {\mathbb {N}}$. We have

4.1 $$\begin{aligned} \frac{\mu _{n,\alpha }}{1-\alpha } \le \pi ^{- \frac{n}{2}} \sqrt{6} \, \frac{\Gamma \left( \frac{n}{2} + 1\right) }{\Gamma \left( \frac{3}{2} \right) } =:C_n \qquad \forall \alpha \in (0,1) \end{aligned}$$

and

4.2 $$\begin{aligned} \lim _{\alpha \rightarrow 1^-}\frac{\mu _{n,\alpha }}{1-\alpha }=\omega _n^{-1}. \end{aligned}$$

#### Proof

Since $\Gamma (1) = 1$ and $\Gamma (1 + x) = x \, \Gamma (x)$ for $x > 0$ (see [9]), we have $\Gamma (x) \sim x^{-1}$ as $x \rightarrow 0^+$. Thus as $\alpha \rightarrow 1^-$ we find

$$\begin{aligned} \mu _{n, \alpha } = 2^{\alpha } \pi ^{- \frac{n}{2}} \frac{\Gamma \left( \frac{n + \alpha + 1}{2} \right) }{\Gamma \left( \frac{1 - \alpha }{2} \right) } \sim \pi ^{- \frac{n}{2}}\,(1 - \alpha )\,\Gamma \left( \frac{n}{2} + 1 \right) = \omega _{n}^{-1} (1 - \alpha ) \end{aligned}$$

and (4.2) follows. Since $\Gamma$ is log-convex on $(0,+\infty )$ (see [9]), for all $x > 0$ and $a \in (0, 1)$ we have

$$\begin{aligned} \Gamma (x + a) = \Gamma ((1 - a)x + a(x + 1)) \le \Gamma (x)^{1 - a}\, \Gamma (x + 1)^{a} = x^{a}\, \Gamma (x). \end{aligned}$$

For $x = \frac{n}{2}$ and $a = \frac{\alpha + 1}{2}$, we can estimate

$$\begin{aligned} \Gamma \left( \frac{n + \alpha + 1}{2} \right) \le \left( \frac{n}{2} \right) ^{\frac{\alpha + 1}{2}} \Gamma \left( \frac{n}{2} \right) \le \max \left\{ \frac{n}{2}, 1 \right\} \Gamma \left( \frac{n}{2} \right) \le n \Gamma \left( \frac{n}{2} \right) = 2 \Gamma \left( \frac{n}{2} + 1 \right) \end{aligned}$$

for all $n\ge 1$. For $x = 1 + \frac{1 - \alpha }{2}$ and $a = \frac{\alpha }{2}$, we can estimate

$$\begin{aligned} \Gamma \left( \frac{3}{2} \right) \le \left( 1 + \frac{1 - \alpha }{2} \right) ^{\frac{\alpha }{2}} \Gamma \left( 1+ \frac{1 - \alpha }{2} \right) \le \sqrt{ \frac{3}{2}}\, \frac{1 - \alpha }{2}\, \Gamma \left( \frac{1 - \alpha }{2} \right) . \end{aligned}$$

We thus get

$$\begin{aligned} \mu _{n, \alpha } (1 - \alpha )^{-1} = 2^{\alpha - 1} \, \pi ^{- \frac{n}{2}}\, \frac{\Gamma \left( \frac{n + \alpha + 1}{2} \right) }{\Gamma \left( \frac{1 - \alpha }{2} + 1 \right) } \le \pi ^{- \frac{n}{2}} \sqrt{ \frac{3}{2}} \frac{2 \Gamma \left( \frac{n}{2} + 1 \right) }{\Gamma \left( \frac{3}{2} \right) } \end{aligned}$$

and (4.1) follows. $$\square $$

In the following technical result, we show that the constant $C_{n, \alpha , U}$ defined in (2.9) is uniformly bounded as $\alpha \rightarrow 1^{-}$ in terms of the volume and the diameter of the bounded open set $U\subset {\mathbb {R}}^n$.

#### Lemma 4.2

(Uniform upper bound on $C_{n, \alpha , U}$ as $\alpha \rightarrow 1^-$). Let $n \in {\mathbb {N}}$ and $\alpha \in (\frac{1}{2}, 1)$. Let $U\subset {\mathbb {R}}^n$ be bounded open set. If $C_{n, \alpha , U}$ is as in (2.9), then

4.3 $$\begin{aligned} C_{n, \alpha , U} \le \frac{n\omega _n C_{n}}{\left( n - \frac{1}{2} \right) } \left( \frac{n}{\left( n - \frac{1}{2} \right) } \, \max \Bigg \{1,\frac{|U|}{\omega _{n}}\Bigg \}^{\frac{1}{n}} +\max \Bigg \{1,\sqrt{{{\,\mathrm{diam}\,}}(U)}\Bigg \} \right) =: \kappa _{n, U},\nonumber \\ \end{aligned}$$

where $C_{n}$ is as in (4.1).

#### Proof

By (4.1), for all $\alpha \in (\frac{1}{2},1)$ we have

$$\begin{aligned} \frac{ n \, \mu _{n, \alpha }}{(n + \alpha - 1) (1 - \alpha )} \le \frac{n \, C_{n}}{n + \alpha - 1} \le \frac{n \, C_{n}}{n - \frac{1}{2}}. \end{aligned}$$

Since $t^{1 - \alpha } \le \max \{1, \sqrt{t}\}$ for any $t\ge 0$ and $\alpha \in (\frac{1}{2},1)$, we have

$$\begin{aligned} \omega _n({{\,\mathrm{diam}\,}}(U))^{1 - \alpha }\le \omega _n\max \Bigg \{1,\sqrt{{{\,\mathrm{diam}\,}}(U)}\Bigg \} \end{aligned}$$

and

$$\begin{aligned} \left( \frac{n \omega _{n}}{n+\alpha -1}\right) ^\frac{n + \alpha - 1}{n}|U|^\frac{1 - \alpha }{n}&=\frac{n\omega _n}{n+\alpha -1}\left( \frac{|U|(n+\alpha -1)}{n\omega _n}\right) ^{\frac{1-\alpha }{n}}\\&\le \frac{n\omega _n}{\left( n - \frac{1}{2} \right) } \max \Bigg \{1,\frac{|U|}{\omega _{n}}\Bigg \}^{\frac{1}{n}}. \end{aligned}$$

Combining these inequalities, we get the conclusion. $\square$

As consequence of Proposition 2.1 and Lemma 4.2, we prove that $\nabla ^\alpha$ and $\mathrm {div}^\alpha$ converge pointwise to $\nabla$ and $\mathrm {div}$ respectively as $\alpha \rightarrow 1^{-}$.

#### Proposition 4.3

If $f\in C^1_c({\mathbb {R}}^n)$, then for all $x\in {\mathbb {R}}^n$ we have

4.4 $$\begin{aligned} \lim _{\alpha \rightarrow 0^-}I_{\alpha }f(x)=f(x). \end{aligned}$$

As a consequence, if $f \in C^{2}_{c}({\mathbb {R}}^{n})$ and $\varphi \in C^{2}_c({\mathbb {R}}^{n}; {\mathbb {R}}^{n})$, then for all $x\in {\mathbb {R}}^n$ we have

4.5 $$\begin{aligned} \lim _{\alpha \rightarrow 1^{-}}\nabla ^{\alpha } f(x)=\nabla f(x), \qquad \lim _{\alpha \rightarrow 1^{-}}\mathrm {div}^{\alpha } \varphi (x)=\mathrm {div}\varphi (x). \end{aligned}$$

#### Proof

Let $f \in C^{1}_{c}({\mathbb {R}}^{n})$ and fix $x\in {\mathbb {R}}^n$. Writing (2.6) in spherical coordinates, we find

$$\begin{aligned} I_{\alpha }f(x) = \frac{\mu _{n, 1-\alpha }}{n - \alpha } \lim _{\delta \rightarrow 0} \int _{\partial B_1} \int _{\delta }^{+ \infty } \varrho ^{-1+ \alpha } f(x + \varrho v)\,d \varrho \, d {\mathscr {H}}^{n - 1}(v). \end{aligned}$$

Since $f\in C^{1}_{c}({\mathbb {R}}^{n})$, for each fixed $v\in \partial B_1$ we can integrate by parts in the variable $\varrho$ and get

$$\begin{aligned} \int _{\delta }^{+ \infty } \varrho ^{-1+ \alpha }f(x + \varrho v)\, d \varrho&= \left[ \frac{\varrho ^{\alpha }}{\alpha }\, f(x + \varrho v)\right] _{\varrho =\delta }^{\varrho \rightarrow +\infty } - \frac{1}{\alpha } \int _{\delta }^{+\infty } \varrho ^{\alpha }\, \partial _\varrho (f(x + \varrho v))\, d \varrho \\&= -\frac{\delta ^{\alpha }}{\alpha } \, f(x + \delta v) - \frac{1}{\alpha } \int _{\delta }^{+\infty } \varrho ^{\alpha }\, \partial _\varrho (f(x + \varrho v))\, d \varrho . \end{aligned}$$

Clearly, we have

$$\begin{aligned} \lim _{\delta \rightarrow 0^+}\delta ^{\alpha } \int _{\partial B_1} f(x + \delta v) \, d {\mathscr {H}}^{n - 1}(v) =0. \end{aligned}$$

Thus, by Fubini’s Theorem, we conclude that

4.6 $$\begin{aligned} I_{\alpha } f(x) = - \frac{\mu _{n, 1-\alpha }}{\alpha (n - \alpha )} \int _{0}^{\infty } \int _{\partial B_1} \varrho ^{\alpha }\, \partial _\varrho (f(x + \varrho v))\, d {\mathscr {H}}^{n - 1}(v) \, d \varrho . \end{aligned}$$

Since *f* has compact support and recalling (4.2), we can pass to the limit in (4.6) and get

$$\begin{aligned} \lim _{\alpha \rightarrow 0^+}I_{\alpha } f(x) = - \frac{1}{n \omega _{n}} \int _{\partial B_1} \int _{0}^{\infty } \partial _\varrho ( f(x + \varrho v) ) \, d \varrho \, d {\mathscr {H}}^{n - 1}(v) = f(x), \end{aligned}$$

proving (4.4). The pointwise limits in (4.5) immediately follows by Proposition 2.1. $\square$

In the following crucial result, we improve the pointwise convergence obtained in Proposition 4.3 to strong convergence in $L^p({\mathbb {R}}^n)$ for all $p\in [1,+\infty ]$.

#### Proposition 4.4

Let $p\in [1,+\infty ]$. If $f \in C^{2}_{c}({\mathbb {R}}^{n})$ and $\varphi \in C^{2}_c({\mathbb {R}}^{n}; {\mathbb {R}}^{n})$, then

$$\begin{aligned} \lim _{\alpha \rightarrow 1^{-}}\Vert \nabla ^{\alpha } f-\nabla f\Vert _{L^p({\mathbb {R}}^n;{\mathbb {R}}^n)}=0, \qquad \lim _{\alpha \rightarrow 1^{-}}\Vert \mathrm {div}^{\alpha } \varphi -\mathrm {div}\varphi \Vert _{L^p({\mathbb {R}}^n)}=0. \end{aligned}$$

#### Proof

Let $$f \in C^{2}_{c}({\mathbb {R}}^{n})$$. Since

$$\begin{aligned} \int _{B_1}\frac{dy}{|y|^{n+\alpha -1}}=n\omega _n\int _0^1\frac{d\varrho }{\varrho ^\alpha }=\frac{n\omega _n}{1-\alpha }, \end{aligned}$$

for all $x\in {\mathbb {R}}^n$ we can write

$$\begin{aligned} \frac{n\omega _n\mu _{n,\alpha }}{(1-\alpha )(n+\alpha -1)}\,\nabla f(x) =\frac{\mu _{n,\alpha }}{n+\alpha -1}\int _{B_1}\frac{\nabla f(x)}{|y|^{n+\alpha -1}}\,dy. \end{aligned}$$

Therefore, by (2.6), we have

$$\begin{aligned} \nabla ^\alpha f(x)&-\frac{n\omega _n\mu _{n,\alpha }}{(1-\alpha )(n+\alpha -1)}\,\nabla f(x)\\&=\frac{\mu _{n,\alpha }}{n+\alpha -1}\left( \int _{B_1}\frac{\nabla f(x+y)-\nabla f(x)}{|y|^{n+\alpha -1}}\,dy +\int _{{\mathbb {R}}^n\setminus B_1}\frac{\nabla f(x+y)}{|y|^{n+\alpha -1}}\,dy\right) \end{aligned}$$

for all $x\in {\mathbb {R}}^n$. We now distinguish two cases.

*Case 1:* $p\in [1,+\infty )$. Using the elementary inequality $|v+w|^p\le 2^{p-1}(|v|^p+|w|^p)$ valid for all $v,w\in {\mathbb {R}}^n$, we have

$$\begin{aligned}&\int _{{\mathbb {R}}^n}\bigg |\,\nabla ^\alpha f(x)-\frac{n\omega _n\mu _{n,\alpha }}{(1-\alpha )(n+\alpha -1)}\,\nabla f(x)\,\bigg |^p dx\\&\quad \le \frac{2^{p-1}\mu _{n,\alpha }}{n+\alpha -1}\int _{{\mathbb {R}}^n}\bigg |\int _{B_1}\frac{\nabla f(x+y)-\nabla f(x)}{|y|^{n+\alpha -1}}\,dy\,\bigg |^p dx\\&\qquad +\frac{2^{p-1}\mu _{n,\alpha }}{n+\alpha -1}\int _{{\mathbb {R}}^n}\bigg |\int _{{\mathbb {R}}^n\setminus B_1}\frac{\nabla f(x+y)}{|y|^{n+\alpha -1}}\,dy\,\bigg |^p dx. \end{aligned}$$

We now estimate the two double integrals appearing in the right-hand side separately.

For the first double integral, as in the proof of Proposition 4.3, we pass in spherical coordinates to get

4.7 $$\begin{aligned}&\int _{B_1}\frac{\nabla f(x+y)-\nabla f(x)}{|y|^{n+\alpha -1}}\,dy\nonumber \\&\quad =\int _{\partial B_1}\int _0^1\varrho ^{-\alpha }\left( \nabla f(x+\varrho v)-\nabla f(x)\right) \,d\varrho \,d{\mathscr {H}}^{n-1}(v)\nonumber \\&\quad =\frac{1}{1-\alpha }\int _{\partial B_1}\left( \nabla f(x+v)-\nabla f(x)\right) \,d{\mathscr {H}}^{n-1}(v)\nonumber \\&\qquad -\int _{\partial B_1}\int _0^1\frac{\varrho ^{1-\alpha }}{1-\alpha }\,\partial _\varrho (\nabla f(x+\varrho v))\,d\varrho \,d{\mathscr {H}}^{n-1}(v) \end{aligned}$$

for all $x\in {\mathbb {R}}^n$. Hence, by (4.2), we find

$$\begin{aligned}&\lim _{\alpha \rightarrow 1^-}\frac{\mu _{n,\alpha }}{(1-\alpha )(n+\alpha -1)}\int _{\partial B_1}\left( \nabla f(x+v)-\nabla f(x)\right) \,d{\mathscr {H}}^{n-1}(v)\\&\quad =\frac{1}{n\omega _n}\int _{\partial B_1}\left( \nabla f(x+v)-\nabla f(x)\right) \,d{\mathscr {H}}^{n-1}(v) \end{aligned}$$

and

$$\begin{aligned}&\lim _{\alpha \rightarrow 1^-}\frac{\mu _{n,\alpha }}{(1-\alpha )(n+\alpha -1)}\int _{\partial B_1}\int _0^1\varrho ^{1-\alpha }\,\partial _\varrho (\nabla f(x+\varrho v))\,d\varrho \,d{\mathscr {H}}^{n-1}(v)\\&\quad =\frac{1}{n\omega _n}\int _{\partial B_1}\int _0^1\partial _\varrho (\nabla f(x+\varrho v))\,d\varrho \,d{\mathscr {H}}^{n-1}(v)\\&\quad =\frac{1}{n\omega _n}\int _{\partial B_1}\left( \nabla f(x+v)-\nabla f(x)\right) \,d{\mathscr {H}}^{n-1}(v) \end{aligned}$$

for all $x\in {\mathbb {R}}^n$. Therefore, we get

$$\begin{aligned} \lim _{\alpha \rightarrow 1^-}\frac{\mu _{n,\alpha }}{n+\alpha -1}\int _{B_1}\frac{\nabla f(x+y)-\nabla f(x)}{|y|^{n+\alpha -1}}\,dy=0 \end{aligned}$$

for all $x\in {\mathbb {R}}^n$. Recalling (4.1), we also observe that

$$\begin{aligned} \frac{\mu _{n,\alpha }}{n+\alpha -1}\frac{|\nabla f(x+y)-\nabla f(x)|}{|y|^{n+\alpha -1}}\le C_n\frac{\Bigg |\nabla f(x+y)-\nabla f(x)\Bigg |}{|y|^n} \end{aligned}$$

for all $\alpha \in (0,1)$, $x\in {\mathbb {R}}^n$ and $y\in B_1$. Moreover, letting $R>0$ be such that ${{\,\mathrm{supp}\,}}f\subset B_R$, we can estimate

$$\begin{aligned} \int _{B_1}\frac{\Bigg |\nabla f(x+y)-\nabla f(x)\Bigg |}{|y|^n}\,dy \le n\omega _n \,\Vert \nabla ^2 f\Vert _{L^\infty \left( {\mathbb {R}}^n;\,{\mathbb {R}}^{n^2}\right) }\,\chi _{B_{R+1}}(x) \end{aligned}$$

for all $x\in {\mathbb {R}}^n$, so that

$$\begin{aligned} x\mapsto \left( \int _{B_1}\frac{\Bigg |\nabla f(x+y)-\nabla f(x)\Bigg |}{|y|^n}\,dy\right) ^p\in L^1({\mathbb {R}}^n). \end{aligned}$$

In conclusion, applying Lebesgue’s Dominated Convergence Theorem, we find

$$\begin{aligned} \lim _{\alpha \rightarrow 1^-}\frac{\mu _{n,\alpha }}{n+\alpha -1}\int _{{\mathbb {R}}^n}\Bigg |\int _{B_1}\frac{\nabla f(x+y)-\nabla f(x)}{|y|^{n+\alpha -1}}\,dy\,\Bigg |^p dx=0. \end{aligned}$$

For the second double integral, note that

$$\begin{aligned} \int _{{\mathbb {R}}^n\setminus B_1}\frac{\nabla f(x+y)}{|y|^{n+\alpha -1}}\,dy =\int _{{\mathbb {R}}^n\setminus B_1}\frac{\nabla (f(x+y)-f(x))}{|y|^{n+\alpha -1}}\,dy \end{aligned}$$

for all $x\in {\mathbb {R}}^n$. Now let $R>0$. Integrating by parts, we have that

$$\begin{aligned} \int _{B_R\setminus B_1}\frac{\nabla (f(x+y)-f(x))}{|y|^{n+\alpha -1}}\,dy&=(n+\alpha -1)\int _{B_R\setminus B_1}\frac{y\,(f(x+y)-f(x))}{|y|^{n+\alpha +1}}\,dy\\&\quad +\frac{1}{R^{n+\alpha -1}}\int _{\partial B_R}(f(x+y)-f(x))\,d{\mathscr {H}}^{n-1}(y)\\&\quad -\int _{\partial B_1}(f(x+y)-f(x))\,d{\mathscr {H}}^{n-1}(y) \end{aligned}$$

for all $x\in {\mathbb {R}}^n$. Since

$$\begin{aligned} \int _{{\mathbb {R}}^n\setminus B_R}\frac{|f(x+y)-f(x)|}{|y|^{n+\alpha }}\,dy \le \frac{2n\omega _n}{\alpha R^\alpha }\Vert f\Vert _{L^\infty ({\mathbb {R}}^n)} \end{aligned}$$

and

$$\begin{aligned} \frac{1}{R^{n+\alpha -1}}\int _{\partial B_R}|f(x+y)-f(x)|\,d{\mathscr {H}}^{n-1}(y) \le \frac{2n\omega _n}{R^\alpha }\Vert f\Vert _{L^\infty ({\mathbb {R}}^n)} \end{aligned}$$

for all $R>0$, we conclude that

4.8 $$\begin{aligned} \begin{aligned} \int _{{\mathbb {R}}^n\setminus B_1}\frac{\nabla f(x+y)}{|y|^{n+\alpha -1}}\,dy&=\lim _{R\rightarrow +\infty }\int _{B_R\setminus B_1}\frac{\nabla f(x+y)}{|y|^{n+\alpha -1}}\,dy\\&=(n+\alpha -1)\int _{{\mathbb {R}}^n\setminus B_1}\frac{y\,(f(x+y)-f(x))}{|y|^{n+\alpha +1}}\,dy\\&\quad -\int _{\partial B_1}(f(x+y)-f(x))\,d{\mathscr {H}}^{n-1}(y) \end{aligned} \end{aligned}$$

for all $x\in {\mathbb {R}}^n$. Hence, by Minkowski’s Integral Inequality (see [76,  Section A.1], for example), we can estimate

$$\begin{aligned} \bigg \Vert \int _{{\mathbb {R}}^n\setminus B_1}\frac{\nabla f(\cdot +y)}{|y|^{n+\alpha -1}}\,dy\,\bigg \Vert _{L^p({\mathbb {R}}^n;\,{\mathbb {R}}^n)}&\le (n+\alpha -1)\bigg \Vert \int _{{\mathbb {R}}^n\setminus B_1}\frac{|f(\cdot +y)-f(\cdot )|}{|y|^{n+\alpha }}\,dy\,\bigg \Vert _{L^p({\mathbb {R}}^n)}\\&\quad +\bigg \Vert \int _{\partial B_1}|f(\cdot +y)-f(\cdot )|\,d{\mathscr {H}}^{n-1}(y)\,\bigg \Vert _{L^p({\mathbb {R}}^n)}\\&\le \frac{n+2\alpha -1}{\alpha }\,2n\omega _n\Vert f\Vert _{L^p({\mathbb {R}}^n)}. \end{aligned}$$

Thus, by (4.2), we get that

$$\begin{aligned} \lim _{\alpha \rightarrow 1^-}\frac{\mu _{n,\alpha }}{n+\alpha -1}\int _{{\mathbb {R}}^n}\Bigg |\int _{{\mathbb {R}}^n\setminus B_1}\frac{\nabla f(x+y)}{|y|^{n+\alpha -1}}\,dy\,\Bigg |^p\,dx=0. \end{aligned}$$

*Case 2:* $p=+\infty$. We have

$$\begin{aligned}&\sup _{x\in {\mathbb {R}}^n}\bigg |\nabla ^\alpha f(x)-\frac{n\omega _n\mu _{n,\alpha }}{(1-\alpha )(n+\alpha -1)}\,\nabla f(x)\bigg |\\&\quad \le \frac{\mu _{n,\alpha }}{n+\alpha -1}\left( \sup _{x\in {\mathbb {R}}^n}\Bigg |\int _{B_1}\frac{\nabla f(x+y)-\nabla f(x)}{|y|^{n+\alpha -1}}\,dy\,\Bigg |\right. \\ {}&\qquad \left. +\sup _{x\in {\mathbb {R}}^n}\Bigg |\int _{{\mathbb {R}}^n\setminus B_1}\frac{\nabla f(x+y)}{|y|^{n+\alpha -1}}\,dy\,\Bigg |\right) . \end{aligned}$$

Again we estimate the two integrals appearing in the right-hand side separately. We note that

$$\begin{aligned}&\int _{\partial B_1}\left( \nabla f(x+v)-\nabla f(x)\right) \,d{\mathscr {H}}^{n-1}(v)\\&\quad -\int _{\partial B_1}\int _0^1\varrho ^{1-\alpha }\,\partial _\varrho (\nabla f(x+\varrho v))\,d\varrho \,d{\mathscr {H}}^{n-1}(v)\\&\quad =\int _{\partial B_1}\int _0^1(1-\varrho ^{1-\alpha })\,\partial _\varrho (\nabla f(x+\varrho v))\,d\varrho \,d{\mathscr {H}}^{n-1}(v), \end{aligned}$$

so that we can rewrite (4.7) as

$$\begin{aligned}&\int _{B_1}\frac{\nabla f(x+y)-\nabla f(x)}{|y|^{n+\alpha -1}}\,dy\\&\quad =\frac{1}{1-\alpha }\int _{\partial B_1}\int _0^1(1-\varrho ^{1-\alpha })\,\partial _\varrho (\nabla f(x+\varrho v))\,d\varrho \,d{\mathscr {H}}^{n-1}(v). \end{aligned}$$

Hence, we can estimate

$$\begin{aligned}&\sup _{x\in {\mathbb {R}}^n}\bigg |\int _{B_1}\frac{\nabla f(x+y)-\nabla f(x)}{|y|^{n+\alpha -1}}\,dy\,\bigg |\\&\quad \le \frac{1}{1-\alpha }\int _{\partial B_1}\int _0^1(1-\varrho ^{1-\alpha })\sup _{x\in {\mathbb {R}}^n}|\partial _\varrho (\nabla f(x+\varrho v))|\,d\varrho \,d{\mathscr {H}}^{n-1}(v)\\&\quad \le \frac{1}{2-\alpha }\,n\omega _n\,\Vert \nabla ^2 f\Vert _{L^\infty \left( {\mathbb {R}}^n;\,{\mathbb {R}}^{n^2}\right) }, \end{aligned}$$

so that

$$\begin{aligned} \lim _{\alpha \rightarrow 1^-}\frac{\mu _{n,\alpha }}{n+\alpha -1}\sup _{x\in {\mathbb {R}}^n}\Bigg |\int _{B_1}\frac{\nabla f(x+y)-\nabla f(x)}{|y|^{n+\alpha -1}}\,dy\,\Bigg |=0. \end{aligned}$$

For the second integral, by (4.8) we can estimate

$$\begin{aligned}&\sup _{x\in {\mathbb {R}}^n}\bigg |\int _{{\mathbb {R}}^n\setminus B_1}\frac{\nabla f(x+y)}{|y|^{n+\alpha -1}}\,dy\,\bigg |\,dx \\&\quad \le (n+\alpha -1)\sup _{x\in {\mathbb {R}}^n}\bigg |\int _{{\mathbb {R}}^n\setminus B_1}\frac{|f(x+y)-f(x)|}{|y|^{n+\alpha }}\,dy\,\bigg |\\&\qquad +\sup _{x\in {\mathbb {R}}^n}\bigg |\int _{\partial B_1}|f(x+y)-f(x)|\,d{\mathscr {H}}^{n-1}(y)\,\bigg |\\&\quad \le \frac{n+2\alpha -1}{\alpha }\,2n\omega _n\Vert f\Vert _{L^\infty ({\mathbb {R}}^n)}. \end{aligned}$$

Thus, by (4.2), we get that

$$\begin{aligned} \lim _{\alpha \rightarrow 1^-}\frac{\mu _{n,\alpha }}{n+\alpha -1}\sup _{x\in {\mathbb {R}}^n}\Bigg |\int _{{\mathbb {R}}^n\setminus B_1}\frac{\nabla f(x+y)}{|y|^{n+\alpha -1}}\,dy\,\Bigg |=0. \end{aligned}$$

We can now conclude the proof. Again recalling (4.2), we thus find that

$$\begin{aligned}&\lim _{\alpha \rightarrow 1^-}\Vert \nabla ^{\alpha } f-\nabla f\Vert _{L^p({\mathbb {R}}^n;{\mathbb {R}}^n)}\\&\quad \le \lim _{\alpha \rightarrow 1^-}\left\| \nabla ^{\alpha } f-\frac{n\omega _n\mu _{n,\alpha }}{(1-\alpha )(n+\alpha -1)}\,\nabla f\,\right\| _{L^p({\mathbb {R}}^n;{\mathbb {R}}^n)}\\&\qquad +\Vert \nabla f\Vert _{L^p({\mathbb {R}}^n;{\mathbb {R}}^n)}\lim _{\alpha \rightarrow 1^-}\left| \frac{n\omega _n\mu _{n,\alpha }}{(1-\alpha )(n+\alpha -1)}-1\right| =0 \end{aligned}$$

for all $p\in [1,+\infty ]$ and the conclusion follows. The $L^p$-convergence of $\mathrm {div}^\alpha \varphi$ to $\mathrm {div}\varphi$ as $\alpha \rightarrow 1^-$ for all $p\in [1,+\infty ]$ follows by a similar argument and is left to the reader. $\square$

#### Remark 4.5

Note that the conclusion of Proposition 4.4 still holds if instead one assumes that $f\in {\mathscr {S}}({\mathbb {R}}^n)$ and $\varphi \in {\mathscr {S}}({\mathbb {R}}^n;{\mathbb {R}}^n)$, where ${\mathscr {S}}({\mathbb {R}}^n;{\mathbb {R}}^m)$ is the space of *m*-vector-valued Schwartz functions. We leave the proof of this assertion to the reader.

### Weak convergence of $\alpha$-variation as $\alpha \rightarrow 1^-$

In Theorem 4.7 below, we prove that the fractional $\alpha$-variation weakly converges to the standard variation as $\alpha \rightarrow 1^-$ for functions either in $BV({\mathbb {R}}^{n})$ or in $BV_{{{\,\mathrm{loc}\,}}}({\mathbb {R}}^{n})\cap L^{\infty }({\mathbb {R}}^{n})$. In the proof of Theorem 4.7, we are going to use the following technical result.

#### Lemma 4.6

There exists a dimensional constant $c_n>0$ with the following property. If $f \in L^{\infty }({\mathbb {R}}^{n})\cap BV_{{{\,\mathrm{loc}\,}}}({\mathbb {R}}^{n})$, then

4.9 $$\begin{aligned} \Vert \nabla ^{\alpha } f \Vert _{L^{1}(B_{R};\, {\mathbb {R}}^{n})} \le c_n\left( R^{1-\alpha }|Df|(B_{3R}) + R^{n-\alpha }\,\Vert f\Vert _{L^{\infty }({\mathbb {R}}^{n})}\right) \end{aligned}$$

for all $R>0$ and $\alpha \in (\frac{1}{2},1)$.

#### Proof

Since $\Gamma (x)\sim x^{-1}$ as $x\rightarrow 0^+$ (see [9]), inequality (4.9) follows immediately combining (3.7) with Lemma 4.1. $\square$

#### Theorem 4.7

If either $f \in BV({\mathbb {R}}^{n})$ or $f \in BV_{{{\,\mathrm{loc}\,}}}({\mathbb {R}}^{n})\cap L^{\infty }({\mathbb {R}}^{n})$, then

$$\begin{aligned} D^\alpha f\rightharpoonup Df \quad \text {as } \alpha \rightarrow 1^-. \end{aligned}$$

#### Proof

We divide the proof in two steps.

*Step 1*. Assume $f \in BV({\mathbb {R}}^{n})$. By [27,  Theorem 3.18], we have

$$\begin{aligned} \int _{{\mathbb {R}}^n}\varphi \cdot \nabla ^\alpha f\,dx =-\int _{{\mathbb {R}}^n} f\,\mathrm {div}^\alpha \varphi \,dx \end{aligned}$$

for all $\varphi \in {{\,\mathrm{Lip}\,}}_c({\mathbb {R}}^n;{\mathbb {R}}^n)$. Thus, given $\varphi \in C^2_c({\mathbb {R}}^n;{\mathbb {R}}^n)$, recalling Proposition 4.3 and the estimates (2.12) and (4.3), by Lebesgue’s Dominated Convergence Theorem we get that

$$\begin{aligned}&\lim _{\alpha \rightarrow 1^-}\int _{{\mathbb {R}}^n}\varphi \cdot \nabla ^\alpha f\,dx\\&\quad =-\lim _{\alpha \rightarrow 1^-}\int _{{\mathbb {R}}^n} f\,\mathrm {div}^\alpha \varphi \,dx =-\int _{{\mathbb {R}}^n} f\,\mathrm {div}\varphi \,dx =\int _{{\mathbb {R}}^n} \varphi \cdot dDf. \end{aligned}$$

To achieve the same limit for any $\varphi \in C_c^0({\mathbb {R}}^n;{\mathbb {R}}^n)$, one just need to exploit (3.3) and the uniform estimate (4.3) in Lemma 4.2, and argue as in Step 2 of the proof of (3.4). We leave the details to the reader.

*Step 2*. Assume $f \in BV_{{{\,\mathrm{loc}\,}}}({\mathbb {R}}^{n})\cap L^{\infty }({\mathbb {R}}^{n})$. By Proposition 3.2(iii), we know that $D^\alpha f=\nabla ^\alpha f{\mathscr {L}}^{n}$ with $\nabla ^\alpha f\in L^1_{{{\,\mathrm{loc}\,}}}({\mathbb {R}}^n;{\mathbb {R}}^n)$. By Proposition 4.4, we get that

$$\begin{aligned}&\lim _{\alpha \rightarrow 1^-}\Bigg |\int _{{\mathbb {R}}^n}\varphi \cdot \nabla ^\alpha f\,dx-\int _{{\mathbb {R}}^n}\varphi \cdot dDf\,\Bigg |\\&\quad \le \Vert f\Vert _{L^\infty ({\mathbb {R}}^n)}\lim _{\alpha \rightarrow 1^-}\Vert \mathrm {div}^\alpha \varphi -\mathrm {div}\varphi \Vert _{L^1({\mathbb {R}}^n;\,{\mathbb {R}}^n)}=0 \end{aligned}$$

for all $\varphi \in C^2_c({\mathbb {R}}^n;{\mathbb {R}}^n)$. To achieve the same limit for any $\varphi \in C_c^0({\mathbb {R}}^n;{\mathbb {R}}^n)$, one just need to exploit (4.9) and argue as in Step 1. We leave the details to the reader. $\square$

We are now going to improve the weak convergence of the fractional $\alpha$-variation obtained in Theorem 4.7 by establishing the weak convergence also of the total fractional $\alpha$-variation as $\alpha \rightarrow 1^-$, see Theorem 4.9 below. To do so, we need the following preliminary result.

#### Lemma 4.8

Let $\mu \in {\mathscr {M}}({\mathbb {R}}^n; {\mathbb {R}}^m)$. We have $(I_{\alpha }\mu ){\mathscr {L}}^{n}\rightharpoonup \mu$ as $\alpha \rightarrow 0^+$.

#### Proof

Since Riesz potential is a linear operator and thanks to Hahn–Banach Decomposition Theorem, without loss of generality we can assume that $\mu$ is a nonnegative finite Radon measure.

Let now $\varphi \in C_c^1({\mathbb {R}}^n)$ and let $U\subset {\mathbb {R}}^n$ be a bounded open set such that ${{\,\mathrm{supp}\,}}\varphi \subset U$. We have that $\Vert I_\alpha |\varphi |\Vert _{L^\infty ({\mathbb {R}}^n)}\le \kappa _{n,U}\Vert \varphi \Vert _{L^\infty ({\mathbb {R}}^n)}$ for all $\alpha \in (0,\frac{1}{2})$ by [27,  Lemma 2.4] and Lemma 4.2. Thus, by (4.4), Fubini’s Theorem and Lebesgue’s Dominated Convergence Theorem, we get that

$$\begin{aligned} \lim _{\alpha \rightarrow 0^+}\int _{{\mathbb {R}}^n} \varphi \,I_\alpha \mu \,dx =\lim _{\alpha \rightarrow 0^+}\int _{{\mathbb {R}}^n} I_\alpha \varphi \,d\mu =\int _{{\mathbb {R}}^n}\varphi \,d\mu . \end{aligned}$$

To achieve the same limit for any $\varphi \in C_c^0({\mathbb {R}}^n)$, one just need to exploit [27,  Lemma 2.4] and (4.3) and argue as in Step 2 of the proof of (3.4). We leave the details to the reader. $\square$

#### Theorem 4.9

If either $f\in BV({\mathbb {R}}^n)$ or $f\in bv({\mathbb {R}}^n)\cap L^\infty ({\mathbb {R}}^n)$, then

4.10 $$\begin{aligned} |D^\alpha f|\rightharpoonup |Df| \quad \text {as } \alpha \rightarrow 1^-. \end{aligned}$$

Moreover, if $f\in BV({\mathbb {R}}^n)$, then also

4.11 $$\begin{aligned} \lim _{\alpha \rightarrow 1^-}|D^\alpha f|({\mathbb {R}}^n) =|Df|({\mathbb {R}}^n). \end{aligned}$$

#### Proof

We prove (4.10) and (4.11) separately.

*Proof of* (4.10). By Theorem 4.7, we know that $D^\alpha f\rightharpoonup Df$ as $\alpha \rightarrow 1^-$. By [50,  Proposition 4.29], we thus have that

4.12 $$\begin{aligned} |Df|(A)\le \liminf _{\alpha \rightarrow 1^-}|D^\alpha f|(A) \end{aligned}$$

for any open set $A\subset {\mathbb {R}}^n$. Now let $K\subset {\mathbb {R}}^n$ be a compact set. By the representation formula (3.18) in Corollary 3.6, we can estimate

$$\begin{aligned} |D^\alpha f|(K) =\Vert \nabla ^\alpha f\Vert _{L^1(K;\,{\mathbb {R}}^n)} \le \Vert I_{1-\alpha }|Df|\Vert _{L^1(K)} =(I_{1-\alpha }|Df|\,{\mathscr {L}}^{n})(K). \end{aligned}$$

Since $|Df|({\mathbb {R}}^n)<+\infty$, by Lemma 4.8 and [50,  Proposition 4.26] we can conclude that

$$\begin{aligned} \limsup _{\alpha \rightarrow 1^{-}} |D^{\alpha } f|(K) \le \limsup _{\alpha \rightarrow 1^{-}} (I_{1-\alpha }|Df|\,{\mathscr {L}}^{n})(K) \le |Df|(K), \end{aligned}$$

and so (4.10) follows, thanks again to [50,  Proposition 4.26].

*Proof of* (4.11). Now assume $f\in BV({\mathbb {R}}^n)$. By (3.4) applied with $A={\mathbb {R}}^n$ and $r=1$, we have

$$\begin{aligned} |D^\alpha f|({\mathbb {R}}^n) \le \frac{n\omega _n\,\mu _{n,\alpha }}{n+\alpha -1}\left( \frac{|Df|({\mathbb {R}}^n)}{1-\alpha }+\frac{n+2\alpha -1}{\alpha }\,\Vert f\Vert _{L^1({\mathbb {R}}^n)}\right) . \end{aligned}$$

By (4.2), we thus get that

4.13 $$\begin{aligned} \limsup _{\alpha \rightarrow 1^-}|D^\alpha f|({\mathbb {R}}^n) \le |D f|({\mathbb {R}}^n). \end{aligned}$$

Thus (4.11) follows by combining (4.12) for $A={\mathbb {R}}^n$ with (4.13). $\square$

#### Remark 4.10

We notice that Theorems 4.7 and 4.9, in the case $f=\chi _E\in BV({\mathbb {R}}^n)$ with $E\subset {\mathbb {R}}^n$ bounded, and Theorem 4.11, were already announced in [71,  Theorems 16 and 17].

Note that Theorems 4.7 and 4.9 in particular apply to any $f\in W^{1,1}({\mathbb {R}}^n)$. In the following result, by exploiting Proposition 3.3, we prove that a stronger property holds for any $f\in W^{1,p}({\mathbb {R}}^n)$ with $p\in [1,+\infty )$.

#### Theorem 4.11

Let $p\in [1,+\infty )$. If $f\in W^{1,p}({\mathbb {R}}^n)$, then

4.14 $$\begin{aligned} \lim _{\alpha \rightarrow 1^-}\Vert \nabla ^\alpha _w f-\nabla _w f\Vert _{L^p({\mathbb {R}}^n;\,{\mathbb {R}}^n)}=0. \end{aligned}$$

#### Proof

By Proposition 3.3 we know that $f\in S^{\alpha ,p}({\mathbb {R}}^n)$ for any $\alpha \in (0, 1)$. We now assume $p\in (1,+\infty )$ and divide the proof in two steps.

*Step 1*. We claim that

4.15 $$\begin{aligned} \lim _{\alpha \rightarrow 1^-}\Vert \nabla ^\alpha _w f\Vert _{L^p({\mathbb {R}}^n;\,{\mathbb {R}}^n)}=\Vert \nabla _w f\Vert _{L^p({\mathbb {R}}^n;\,{\mathbb {R}}^n)}. \end{aligned}$$

Indeed, on the one hand, by Proposition 4.4, we have

4.16 $$\begin{aligned} \int _{{\mathbb {R}}^n}\varphi \cdot \nabla _w f\,dx= & {} -\int _{{\mathbb {R}}^n}f\,\mathrm {div}\varphi \,dx =-\lim _{\alpha \rightarrow 1^-}\int _{{\mathbb {R}}^n}f\,\mathrm {div}^\alpha \varphi \,dx\nonumber \\= & {} \lim _{\alpha \rightarrow 1^-}\int _{{\mathbb {R}}^n}\varphi \cdot \nabla _w^\alpha f\,dx \end{aligned}$$

for all $\varphi \in C^\infty _c({\mathbb {R}}^n;{\mathbb {R}}^n)$, so that

$$\begin{aligned} \int _{{\mathbb {R}}^n}\varphi \cdot \nabla _w f\,dx \le \Vert \varphi \Vert _{L^{\frac{p}{p-1}}({\mathbb {R}}^n;\,{\mathbb {R}}^n)}\liminf _{\alpha \rightarrow 1^-}\Vert \nabla _w^\alpha f\Vert _{L^p({\mathbb {R}}^n;\,{\mathbb {R}}^n)} \end{aligned}$$

for all $\varphi \in C^\infty _c({\mathbb {R}}^n;{\mathbb {R}}^n)$. We thus get that

4.17 $$\begin{aligned} \Vert \nabla _w f\Vert _{L^p({\mathbb {R}}^n;{\mathbb {R}}^n)} \le \liminf _{\alpha \rightarrow 1^-}\Vert \nabla ^\alpha _w f\Vert _{L^p({\mathbb {R}}^n;{\mathbb {R}}^n)}. \end{aligned}$$

On the other hand, applying (3.10) with $A={\mathbb {R}}^n$ and $r=1$, we have

$$\begin{aligned} \Vert \nabla ^\alpha _w f\Vert _{L^p({\mathbb {R}}^n;\,{\mathbb {R}}^n)} \le \frac{n\omega _n\,\mu _{n,\alpha }}{n+\alpha -1}\left( \frac{\Vert \nabla _w f\Vert _{L^p({\mathbb {R}}^n;\,{\mathbb {R}}^n)}}{1-\alpha }+\frac{n+2\alpha -1}{\alpha }\,\Vert f\Vert _{L^p({\mathbb {R}}^n)}\right) . \end{aligned}$$

By (4.2), we conclude that

4.18 $$\begin{aligned} \limsup _{\alpha \rightarrow 1^-}\Vert \nabla ^\alpha _w f\Vert _{L^p({\mathbb {R}}^n;\,{\mathbb {R}}^n)} \le \Vert \nabla _w f\Vert _{L^p({\mathbb {R}}^n;\,{\mathbb {R}}^n)}. \end{aligned}$$

Thus, (4.15) follows by combining (4.17) and (4.18).

*Step 2*. We now claim that

4.19 $$\begin{aligned} \nabla ^\alpha _w f\rightharpoonup \nabla _w f \quad \text {in }L^p({\mathbb {R}}^n;{\mathbb {R}}^n) \text { as } \alpha \rightarrow 1^-. \end{aligned}$$

Indeed, let $\varphi \in L^{\frac{p}{p-1}}({\mathbb {R}}^n;{\mathbb {R}}^n)$. For each $\varepsilon >0$, let $\psi _\varepsilon \in C^\infty _c({\mathbb {R}}^n;{\mathbb {R}}^n)$ be such that $\Vert \psi _\varepsilon -\varphi \Vert _{L^{\frac{p}{p-1}}({\mathbb {R}}^n;\,{\mathbb {R}}^n)}<\varepsilon$. By (4.16) and (4.15), we can estimate

$$\begin{aligned}&\limsup _{\alpha \rightarrow 1^-}\bigg |\int _{{\mathbb {R}}^n}\varphi \cdot \nabla ^\alpha _w f\,dx-\int _{{\mathbb {R}}^n}\varphi \cdot \nabla _w f\,dx\,\bigg |\\&\quad \le \limsup _{\alpha \rightarrow 1^{-}} \bigg |\int _{{\mathbb {R}}^n}\psi _{\varepsilon }\cdot \nabla ^\alpha _w f\,dx-\int _{{\mathbb {R}}^n}\psi _{\varepsilon }\cdot \nabla _w f\,dx\,\bigg | \\&\qquad + \int _{{\mathbb {R}}^n} |\varphi - \psi _{\varepsilon }| \, |\nabla ^\alpha _w f| \,dx + \int _{{\mathbb {R}}^n}|\varphi - \psi _{\varepsilon }| \, |\nabla _w f|\,dx \\&\quad \le \varepsilon \,\bigg (\lim _{\alpha \rightarrow 1^-}\Vert \nabla ^\alpha _w f\Vert _{L^p({\mathbb {R}}^n;\,{\mathbb {R}}^n)}+\Vert \nabla _w f\Vert _{L^p({\mathbb {R}}^n;\,{\mathbb {R}}^n)}\bigg )\\&\quad =2\varepsilon \,\Vert \nabla _w f\Vert _{L^p({\mathbb {R}}^n;\,{\mathbb {R}}^n)} \end{aligned}$$

so that (4.19) follows passing to the limit as $\varepsilon \rightarrow 0^+$.

Since $L^p({\mathbb {R}}^n;{\mathbb {R}}^n)$ is uniformly convex (see [19,  Section 4.3] for example), the limit in (4.14) follows from (4.15) and (4.19) by [19,  Proposition 3.32], and the proof in the case $p\in (1,+\infty )$ is complete.

For the case $p=1$, we argue as follows (we thank Mattia Calzi for this simple argument). Without loss of generality, it is enough to prove the limit in (4.15) with $p=1$ for any given sequence $(\alpha _k)_{k\in {\mathbb {N}}}$ such that $\alpha _k\rightarrow 1^-$ as $k\rightarrow +\infty$. By (4.11), the sequence $(\Vert \nabla ^{\alpha _k} f\Vert _{L^1({\mathbb {R}}^n;\,{\mathbb {R}}^n)})_{k\in {\mathbb {N}}}$ is bounded for any $f\in W^{1,1}({\mathbb {R}}^n)$ and thus, by Banach–Steinhaus Theorem, the linear operators $\nabla ^{\alpha _k}:W^{1,1}({\mathbb {R}}^n)\rightarrow L^1({\mathbb {R}}^n;{\mathbb {R}}^n)$, $k\in {\mathbb {N}}$, are uniformly bounded (in the operator norm). The conclusion hence follows by exploiting the density of $C^\infty _c({\mathbb {R}}^n)$ in $W^{1,1}({\mathbb {R}}^n)$ and Proposition 4.4. $\square$

For the case $p=+\infty$, we have the following result. The proof is very similar to the one of Theorem 4.11 and is thus left to the reader.

#### Theorem 4.12

If $$f\in W^{1,\infty }({\mathbb {R}}^n)$$, then

4.20 $$\begin{aligned} \nabla ^\alpha _w f\rightharpoonup \nabla _w f \quad \text {in } L^\infty ({\mathbb {R}}^n;{\mathbb {R}}^n) \text { as } \alpha \rightarrow 1^- \end{aligned}$$

and

4.21 $$\begin{aligned} \Vert \nabla _w f\Vert _{L^\infty ({\mathbb {R}}^n;\,{\mathbb {R}}^n)}\le \liminf _{\alpha \rightarrow 1^-}\Vert \nabla ^\alpha _w f\Vert _{L^\infty ({\mathbb {R}}^n;\,{\mathbb {R}}^n)}. \end{aligned}$$

### $\Gamma$-convergence of $\alpha$-variation as $\alpha \rightarrow 1^-$

In this section, we study the $\Gamma$-convergence of the fractional $\alpha$-variation to the standard variation as $\alpha \rightarrow 1^-$.

We begin with the $\Gamma \text { -}\liminf$ inequality.

#### Theorem 4.13

($\Gamma \text { -}\liminf$ inequalities as $\alpha \rightarrow 1^-$) Let $\Omega \subset {\mathbb {R}}^n$ be an open set.

(i) If $(f_\alpha )_{\alpha \in (0,1)}\subset L^1_{{{\,\mathrm{loc}\,}}}({\mathbb {R}}^n)$ satisfies $\sup _{\alpha \in (0,1)}\Vert f_\alpha \Vert _{L^\infty ({\mathbb {R}}^n)}<+\infty$ and $f_\alpha \rightarrow f$ in $L^1_{{{\,\mathrm{loc}\,}}}({\mathbb {R}}^n)$ as $\alpha \rightarrow 1^-$, then
  4.22 $$\begin{aligned} |Df|(\Omega )\le \liminf \limits _{\alpha \rightarrow 1^-}|D^\alpha f_\alpha |(\Omega ). \end{aligned}$$
(ii) If $(f_\alpha )_{\alpha \in (0,1)}\subset L^1({\mathbb {R}}^n)$ satisfies $f_\alpha \rightarrow f$ in $L^1({\mathbb {R}}^n)$ as $\alpha \rightarrow 1^-$, then (4.22) holds.

#### Proof

We prove the two statements separately.

*Proof of (i)*. Let $\varphi \in C^\infty _c(\Omega ;{\mathbb {R}}^n)$ be such that $\Vert \varphi \Vert _{L^\infty (\Omega ;{\mathbb {R}}^n)}\le 1$. Since we can estimate

$$\begin{aligned}&\bigg |\int _{{\mathbb {R}}^n}f_\alpha \,\mathrm {div}^\alpha \varphi \,dx-\int _{{\mathbb {R}}^n}f\,\mathrm {div}\varphi \,dx\bigg |\\&\quad \le \int _{{\mathbb {R}}^n}\Bigg |f_\alpha -f\Bigg |\,\Bigg |\mathrm {div}\varphi \Bigg |\,dx +\int _{{\mathbb {R}}^n}|f_\alpha |\,\Bigg |\mathrm {div}^\alpha \varphi -\mathrm {div}\varphi \Bigg |\,dx\\&\quad \le \Vert \mathrm {div}\varphi \Vert _{L^\infty ({\mathbb {R}}^n;\,{\mathbb {R}}^n)}\int _{{{\,\mathrm{supp}\,}}\varphi }\Bigg |f_\alpha -f\Bigg |\,dx\\&\qquad +\big (\sup _{\alpha \in (0,1)}\Vert f_\alpha \Vert _{L^\infty ({\mathbb {R}}^n)}\big )\,\Vert \mathrm {div}^\alpha \varphi -\mathrm {div}\varphi \Vert _{L^1({\mathbb {R}}^n)}, \end{aligned}$$

by Proposition 4.4 we get that

$$\begin{aligned} \int _{{\mathbb {R}}^n}f\,\mathrm {div}\varphi \,dx =\lim _{\alpha \rightarrow 1^-}\int _{{\mathbb {R}}^n}f_\alpha \,\mathrm {div}^\alpha \varphi \,dx \le \liminf _{\alpha \rightarrow 1^-}|D^\alpha f|(\Omega ) \end{aligned}$$

and the conclusion follows.

*Proof of (ii)*. Let $\varphi \in C^\infty _c(\Omega ;{\mathbb {R}}^n)$ be such that $\Vert \varphi \Vert _{L^\infty (\Omega ;{\mathbb {R}}^n)}\le 1$. Since we can estimate

$$\begin{aligned}&\bigg |\int _{{\mathbb {R}}^n} f_\alpha \,\mathrm {div}^\alpha \varphi \,dx-\int _{{\mathbb {R}}^n}f\,\mathrm {div}\varphi \,dx\bigg |\\&\quad \le \int _{{\mathbb {R}}^n}\Bigg |f_\alpha -f\Bigg |\,\Bigg |\mathrm {div}\varphi \Bigg |\,dx +\int _{{\mathbb {R}}^n}|f_\alpha |\,\Bigg |\mathrm {div}^\alpha \varphi -\mathrm {div}\varphi \Bigg |\,dx\\&\quad \le \Vert \mathrm {div}\varphi \Vert _{L^\infty ({\mathbb {R}}^n)}\Vert f_\alpha -f\Vert _{L^1({\mathbb {R}}^n)}+\Vert \mathrm {div}^\alpha \varphi -\mathrm {div}\varphi \Vert _{L^\infty ({\mathbb {R}}^n)}\Vert f_\alpha \Vert _{L^1({\mathbb {R}}^n)}, \end{aligned}$$

by Proposition 4.4 we get that

$$\begin{aligned} \int _{{\mathbb {R}}^n}f\,\mathrm {div}\varphi \,dx =\lim _{\alpha \rightarrow 1^-}\int _{{\mathbb {R}}^n}f_\alpha \,\mathrm {div}^\alpha \varphi \,dx \le \liminf _{\alpha \rightarrow 1^-}|D^\alpha f_\alpha |(\Omega ) \end{aligned}$$

and the conclusion follows. $\square$

We now pass to the $\Gamma \text { -}\limsup$ inequality.

#### Theorem 4.14

($\Gamma \text { -}\limsup$ inequalities as $\alpha \rightarrow 1^-$) Let $\Omega \subset {\mathbb {R}}^n$ be an open set.

(i) If $f\in BV({\mathbb {R}}^n)$ and either $\Omega$ is bounded or $\Omega ={\mathbb {R}}^n$, then
  4.23 $$\begin{aligned} \limsup _{\alpha \rightarrow 1^-}|D^\alpha f|(\Omega )\le |Df|({\overline{\Omega }}). \end{aligned}$$
(ii) If $f\in BV_{{{\,\mathrm{loc}\,}}}({\mathbb {R}}^n)$ and $\Omega$ is bounded, then
  $$\begin{aligned} \Gamma (L^1_{{{\,\mathrm{loc}\,}}})\text { -}\limsup _{\alpha \rightarrow 1^-}|D^\alpha f|(\Omega )\le |Df|({\overline{\Omega }}). \end{aligned}$$

In addition, if $f=\chi _E$, then the recovering sequences $(f_\alpha )_{\alpha \in (0,1)}$ in (i) and (ii) can be taken such that $f_\alpha =\chi _{E_\alpha }$ for some measurable sets $(E_\alpha )_{\alpha \in (0,1)}$.

#### Proof

Assume $f\in BV({\mathbb {R}}^n)$. By Theorem 4.9, we know that $|D^\alpha f|\rightharpoonup |Df|$ as $\alpha \rightarrow 1^-$. Thus, by [50,  Proposition 4.26], we get that

4.24 $$\begin{aligned} \limsup _{\alpha \rightarrow 1^-}|D^\alpha f|(\Omega ) \le \limsup _{\alpha \rightarrow 1^-}|D^\alpha f|( {}\overline{\Omega }) \le |Df|( {}\overline{\Omega }) \end{aligned}$$

for any bounded open set $\Omega \subset {\mathbb {R}}^n$. If $\Omega ={\mathbb {R}}^n$, then (4.23) follows immediately from (4.11). This concludes the proof of (i).

Now assume that $f\in BV_{{{\,\mathrm{loc}\,}}}({\mathbb {R}}^n)$ and $\Omega$ is bounded. Let $(R_k)_{k\in {\mathbb {N}}}\subset (0,+\infty )$ be a sequence such that $R_k\rightarrow +\infty$ as $k\rightarrow +\infty$ and set $f_k:=f\chi _{B_{R_k}}$ for all $k\in {\mathbb {N}}$. By Theorem A.1, we can choose the sequence $(R_k)_{k\in {\mathbb {N}}}$ such that, in addition, $f_k\in BV({\mathbb {R}}^n)$ with $Df_k=\chi _{B_{R_k}}^\star Df+f^\star D\chi _{B_{R_k}}$ for all $k\in {\mathbb {N}}$. Consequently, $f_k\rightarrow f$ in $L^1_{{{\,\mathrm{loc}\,}}}({\mathbb {R}}^n)$ as $k\rightarrow +\infty$ and, moreover, since $\Omega$ is bounded, $|Df_k|(\Omega )=|Df|(\Omega )$ and $|Df_k|(\partial \Omega )=|Df|(\partial \Omega )$ for all $k\in {\mathbb {N}}$ sufficiently large. By (4.24), we have that

4.25 $$\begin{aligned} \limsup _{\alpha \rightarrow 1^-}|D^\alpha f_k|(\Omega ) \le |Df_k|({\overline{\Omega }}) \end{aligned}$$

for all $k\in {\mathbb {N}}$ sufficiently large. Hence, by [17,  Proposition 1.28], by [29,  Proposition 8.1(c)] and by (4.25), we get that

$$\begin{aligned} \Gamma (L^1_{{{\,\mathrm{loc}\,}}})\text { -}\limsup _{\alpha \rightarrow 1^-}|D^\alpha f|(\Omega )&\le \liminf _{k\rightarrow +\infty }\big (\Gamma (L^1_{{{\,\mathrm{loc}\,}}})\text { -}\limsup _{\alpha \rightarrow 1^-}|D^\alpha f_k|(\Omega )\big )\\&\le \lim _{k\rightarrow +\infty }|Df_k|({\overline{\Omega }}) =|Df|({\overline{\Omega }}). \end{aligned}$$

This concludes the proof of (ii).

Finally, if $f=\chi _E$, then we can repeat the above argument *verbatim* in the metric spaces $\{\chi _F\in L^1({\mathbb {R}}^n): F\subset {\mathbb {R}}^n\}$ for (i) and $\{\chi _F\in L^1_{{{\,\mathrm{loc}\,}}}({\mathbb {R}}^n): F\subset {\mathbb {R}}^n\}$ for (ii) endowed with their natural distances. $\square$

#### Remark 4.15

Thanks to (4.23), a *recovery sequence* in Theorem 4.14(i) is the constant sequence (also in the special case $f = \chi _{E}$).

Combining Theorems 4.13(i) and 4.14(ii), we can prove that the fractional Caccioppoli $\alpha$-perimeter $\Gamma$-converges to De Giorgi’s perimeter as $\alpha \rightarrow 1^-$ in $L^1_{{{\,\mathrm{loc}\,}}}({\mathbb {R}}^n)$. We refer to [3] for the same result on the classical fractional perimeter.

#### Theorem 4.16

($\Gamma (L^1_{{{\,\mathrm{loc}\,}}})\text { -}\lim$ of perimeters as $\alpha \rightarrow 1^-$) Let $\Omega \subset {\mathbb {R}}^n$ be a bounded open set with Lipschitz boundary. For every measurable set $E\subset {\mathbb {R}}^n$, we have

$$\begin{aligned} \Gamma (L^1_{{{\,\mathrm{loc}\,}}})\text { -}\lim _{\alpha \rightarrow 1^-}|D^\alpha \chi _E|(\Omega ) =P(E;\Omega ). \end{aligned}$$

#### Proof

By Theorem 4.13(i), we already know that

$$\begin{aligned} \Gamma (L^1_{{{\,\mathrm{loc}\,}}})\text { -}\liminf _{\alpha \rightarrow 1^-}|D^\alpha \chi _E|(\Omega ) \ge P(E;\Omega ), \end{aligned}$$

so we just need to prove the $\Gamma (L^1_{{{\,\mathrm{loc}\,}}})\text { -}\limsup$ inequality. Without loss of generality, we can assume $P(E;\Omega )<+\infty$. Now let $(E_k)_{k\in {\mathbb {N}}}$ be given by Theorem A.4. Since $\chi _{E_k}\in BV_{{{\,\mathrm{loc}\,}}}({\mathbb {R}}^n)$ and $P(E_k;\partial \Omega )=0$ for all $k\in {\mathbb {N}}$, by Theorem 4.14(ii) we know that

$$\begin{aligned} \Gamma (L^1_{{{\,\mathrm{loc}\,}}})\text { -}\limsup _{\alpha \rightarrow 1^-}|D^\alpha \chi _{E_k}|(\Omega )\le P(E_k;\Omega ) \end{aligned}$$

for all $k\in {\mathbb {N}}$. Since $\chi _{E_k}\rightarrow \chi _E$ in $L^1_{{{\,\mathrm{loc}\,}}}({\mathbb {R}}^n)$ and $P(E_k;\Omega )\rightarrow P(E;\Omega )$ as $k\rightarrow +\infty$, by [17,  Proposition 1.28] we get that

$$\begin{aligned} \Gamma (L^1_{{{\,\mathrm{loc}\,}}})\text { -}\limsup _{\alpha \rightarrow 1^-}|D^\alpha \chi _E|(\Omega )&\le \liminf _{k\rightarrow +\infty }\big (\Gamma (L^1_{{{\,\mathrm{loc}\,}}})\text { -}\limsup _{\alpha \rightarrow 1^-}|D^\alpha \chi _{E_k}|(\Omega )\big )\\&\le \lim _{k\rightarrow +\infty } P(E_k;\Omega ) =P(E;\Omega ) \end{aligned}$$

and the proof is complete. $\square$

Finally, by combining Theorems 4.13(ii) and 4.14, we can prove that the fractional $\alpha$-variation $\Gamma$-converges to De Giorgi’s variation as $\alpha \rightarrow 1^-$ in $L^1({\mathbb {R}}^n)$.

#### Theorem 4.17

($\Gamma (L^1)\text { -}\lim$ of variations as $\alpha \rightarrow 1^-$) Let $\Omega \subset {\mathbb {R}}^n$ be an open set such that either $\Omega$ is bounded with Lipschitz boundary or $\Omega ={\mathbb {R}}^n$. For every $f\in BV({\mathbb {R}}^n)$, we have

$$\begin{aligned} \Gamma (L^1)\text { -}\lim _{\alpha \rightarrow 1^-}|D^\alpha f|(\Omega ) =|Df|(\Omega ). \end{aligned}$$

#### Proof

The case $\Omega ={\mathbb {R}}^n$ follows immediately by [29,  Proposition 8.1(c)] combining Theorem 4.13(ii) with Theorem 4.14(i). We can thus assume that $\Omega$ is a bounded open set with Lipschitz boundary and argue as in the proof of Theorem 4.16. By Theorem 4.13(ii), we already know that

$$\begin{aligned} \Gamma (L^1)\text { -}\liminf _{\alpha \rightarrow 1^-}|D^\alpha f|(\Omega ) \ge |Df|(\Omega ), \end{aligned}$$

so we just need to prove the $\Gamma (L^1)\text { -}\limsup$ inequality. Without loss of generality, we can assume $|Df|(\Omega )<+\infty$. Now let $(f_k)_{k\in {\mathbb {N}}}\subset BV({\mathbb {R}}^n)$ be given by Theorem A.6. Since $|Df_k|(\partial \Omega )=0$ for all $k\in {\mathbb {N}}$, by Theorem 4.14 we know that

$$\begin{aligned} \Gamma (L^1)\text { -}\limsup _{\alpha \rightarrow 1^-}|D^\alpha f_k|(\Omega ) \le |Df_k|({\overline{\Omega }}) =|Df_k|(\Omega ) \end{aligned}$$

for all $k\in {\mathbb {N}}$. Since $f_k\rightarrow f$ in $L^1({\mathbb {R}}^n)$ and $|D^\alpha f_k|(\Omega )\rightarrow |D^\alpha f|(\Omega )$ as $k\rightarrow +\infty$, by [17,  Proposition 1.28] we get that

$$\begin{aligned} \Gamma (L^1)\text { -}\limsup _{\alpha \rightarrow 1^-}|D^\alpha f|(\Omega )&\le \liminf _{k\rightarrow +\infty }\big (\Gamma (L^1)\text { -}\limsup _{\alpha \rightarrow 1^-}|D^\alpha f_k|(\Omega )\big )\\&\le \lim _{k\rightarrow +\infty } |Df_k|(\Omega ) =|Df|(\Omega ) \end{aligned}$$

and the proof is complete. $\square$

#### Remark 4.18

Thanks to Theorem 4.17, we can slightly improve Theorem 4.16. Indeed, if $\chi _{E} \in BV({\mathbb {R}}^{n})$, then we also have

$$\begin{aligned} \Gamma (L^1)\text { -}\lim _{\alpha \rightarrow 1^-}|D^\alpha \chi _E|(\Omega ) =|D\chi _E|(\Omega ) \end{aligned}$$

for any open set $\Omega \subset {\mathbb {R}}^n$ such that either $\Omega$ is bounded with Lipschitz boundary or $\Omega ={\mathbb {R}}^n$.

## Asymptotic behavior of fractional $\beta$-variation as $\beta \rightarrow \alpha ^-$

### Convergence of $\nabla ^\beta$ and $\mathrm {div}^\beta$ as $\beta \rightarrow \alpha$

We begin with the following simple result about the $L^1$-convergence of the operators $\nabla ^\beta$ and $\mathrm {div}^\beta$ as $\beta \rightarrow \alpha$ with $\alpha \in (0,1)$.

#### Lemma 5.1

Let $\alpha \in (0,1)$. If $f\in W^{\alpha ,1}({\mathbb {R}}^n)$ and $\varphi \in W^{\alpha ,1}({\mathbb {R}}^n;{\mathbb {R}}^n)$, then

5.1 $$\begin{aligned} \lim _{\beta \rightarrow \alpha ^-}\Vert \nabla ^\beta f-\nabla ^\alpha f\Vert _{L^1({\mathbb {R}}^n;\,{\mathbb {R}}^n)}=0, \qquad \lim _{\beta \rightarrow \alpha ^-}\Vert \mathrm {div}^\beta \varphi -\mathrm {div}^\alpha \varphi \Vert _{L^1({\mathbb {R}}^n)}=0.\nonumber \\ \end{aligned}$$

#### Proof

Given $\beta \in (0,\alpha )$, we can estimate

$$\begin{aligned}&\int _{{\mathbb {R}}^n}|\nabla ^\beta f(x)-\nabla ^\alpha f(x)|\,dx \le |\mu _{n,\beta }-\mu _{n,\alpha }|\,[f]_{W^{\alpha ,1}({\mathbb {R}}^n)}\\&\qquad +\mu _{n,\beta }\int _{{\mathbb {R}}^n}\int _{{\mathbb {R}}^n}\frac{|f(y)-f(x)|}{|y-x|^n}\,\Bigg |\frac{1}{|y-x|^\beta }-\frac{1}{|y-x|^\alpha }\Bigg |\,dy\,dx. \end{aligned}$$

Since the $\Gamma$ function is continuous (see [9]), we clearly have

$$\begin{aligned} \lim _{\beta \rightarrow \alpha ^-}|\mu _{n,\beta }-\mu _{n,\alpha }|\,[f]_{W^{\alpha ,1}({\mathbb {R}}^n)}=0. \end{aligned}$$

Now write

$$\begin{aligned}&\int _{{\mathbb {R}}^n}\int _{{\mathbb {R}}^n}\frac{|f(y)-f(x)|}{|y-x|^n}\,\Bigg |\frac{1}{|y-x|^\beta }-\frac{1}{|y-x|^\alpha }\Bigg |\,dy\,dx\\&\quad =\int _{{\mathbb {R}}^n}\int _{{\mathbb {R}}^n}\frac{|f(y)-f(x)|}{|y-x|^n}\,\Bigg |\frac{1}{|y-x|^\beta }-\frac{1}{|y-x|^\alpha }\Bigg |\,\chi _{(0,1)}(|y-x|)\,dy\,dx\\&\qquad +\int _{{\mathbb {R}}^n}\int _{{\mathbb {R}}^n}\frac{|f(y)-f(x)|}{|y-x|^n}\,\Bigg |\frac{1}{|y-x|^\beta }-\frac{1}{|y-x|^\alpha }\Bigg |\,\chi _{[1,+\infty )}(|y-x|)\,dy\,dx. \end{aligned}$$

On the one hand, since $f\in W^{\alpha ,1}({\mathbb {R}}^n)$, we have

$$\begin{aligned}&\frac{|f(y)-f(x)|}{|y-x|^n}\,\Bigg |\frac{1}{|y-x|^\beta }-\frac{1}{|y-x|^\alpha }\Bigg |\,\chi _{(0,1)}(|y-x|)\\&\quad =\frac{|f(y)-f(x)|}{|y-x|^n}\,\left( \frac{1}{|y-x|^\alpha }-\frac{1}{|y-x|^\beta }\right) \chi _{(0,1)}(|y-x|)\\&\quad \le \frac{|f(y)-f(x)|}{|y-x|^{n+\alpha }}\,\chi _{(0,1)}(|y-x|) \in L^1_{x,y}({\mathbb {R}}^{2n}) \end{aligned}$$

and thus, by Lebesgue’s Dominated Convergence Theorem, we get that

$$\begin{aligned} \lim _{\beta \rightarrow \alpha ^-}\int _{{\mathbb {R}}^n}\int _{{\mathbb {R}}^n}\frac{|f(y)-f(x)|}{|y-x|^n}\,\Bigg |\frac{1}{|y-x|^\beta }-\frac{1}{|y-x|^\alpha }\Bigg |\,\chi _{(0,1)}(|y-x|)\,dy\,dx=0. \end{aligned}$$

On the other hand, since one has

$$\begin{aligned}{}[f]_{W^{\beta ,1}({\mathbb {R}}^n)}&=\int _{{\mathbb {R}}^n}\int _{\{|h|<1\}}\frac{|f(x+h)-f(x)|}{|h|^{n+\beta }}\,dh\,dx\\&\quad +\int _{{\mathbb {R}}^n}\int _{\{|h|\ge 1\}}\frac{|f(x+h)-f(x)|}{|h|^{n+\beta }}\,dh\,dx\\&\le [f]_{W^{\alpha ,1}({\mathbb {R}}^n)} +\int _{\{|h|\ge 1\}}\frac{1}{|h|^{n+\beta }}\int _{{\mathbb {R}}^n}|f(x+h)|+|f(x)|\,dx\,dh\\&=[f]_{W^{\alpha ,1}({\mathbb {R}}^n)}+\frac{2n\omega _n}{\beta }\,\Vert f\Vert _{L^1({\mathbb {R}}^n)} \end{aligned}$$

for all $\beta \in (0,\alpha )$, we can estimate

$$\begin{aligned}&\frac{|f(y)-f(x)|}{|y-x|^n}\,\Bigg |\frac{1}{|y-x|^\beta }-\frac{1}{|y-x|^\alpha }\Bigg |\,\chi _{[1,+\infty )}(|y-x|)\\&\quad =\frac{|f(y)-f(x)|}{|y-x|^n}\,\left( \frac{1}{|y-x|^\beta }-\frac{1}{|y-x|^\alpha }\right) \chi _{[1,+\infty )}(|y-x|)\\&\quad \le \frac{|f(y)-f(x)|}{|y-x|^{n+\beta }}\,\chi _{[1,+\infty )}(|y-x|)\\&\quad \le \frac{|f(y)-f(x)|}{|y-x|^{n+\frac{\alpha }{2}}}\,\chi _{[1,+\infty )}(|y-x|) \in L^1_{x,y}({\mathbb {R}}^{2n}) \end{aligned}$$

for all $\beta \in \left( \frac{\alpha }{2},\alpha \right)$ and thus, by Lebesgue’s Dominated Convergence Theorem, we get that

$$\begin{aligned} \lim _{\beta \rightarrow \alpha ^-}\int _{{\mathbb {R}}^n}\int _{{\mathbb {R}}^n}\frac{|f(y)-f(x)|}{|y-x|^n}\,\Bigg |\frac{1}{|y-x|^\beta }-\frac{1}{|y-x|^\alpha }\Bigg |\,\chi _{[1,+\infty )}(|y-x|)\,dy\,dx=0 \end{aligned}$$

and the first limit in (5.1) follows. The second limit in (5.1) follows similarly and we leave the proof to the reader. $\square$

#### Remark 5.2

Let $\alpha \in (0,1)$. If $f\in W^{\alpha +\varepsilon ,1}({\mathbb {R}}^n)$ and $\varphi \in W^{\alpha +\varepsilon ,1}({\mathbb {R}}^n)$ for some $\varepsilon \in (0,1-\alpha )$, then, arguing as in the proof of Lemma 5.1, one can also prove that

$$\begin{aligned} \lim _{\beta \rightarrow \alpha ^+}\Vert \nabla ^\beta f-\nabla ^\alpha f\Vert _{L^1({\mathbb {R}}^n;\,{\mathbb {R}}^n)}=0, \qquad \lim _{\beta \rightarrow \alpha ^+}\Vert \mathrm {div}^\beta \varphi -\mathrm {div}^\alpha \varphi \Vert _{L^1({\mathbb {R}}^n)}=0. \end{aligned}$$

We leave the details of proof of this result to the interested reader.

If one deals with more regular functions, then Lemma 5.1 can be improved as follows.

#### Lemma 5.3

Let $\alpha \in (0,1)$ and $p \in [1, + \infty ]$. If $f\in {{\,\mathrm{Lip}\,}}_{c}({\mathbb {R}}^n)$ and $\varphi \in {{\,\mathrm{Lip}\,}}_{c}({\mathbb {R}}^n;{\mathbb {R}}^n)$, then

5.2 $$\begin{aligned} \lim _{\beta \rightarrow \alpha ^-}\Vert \nabla ^\beta f-\nabla ^\alpha f\Vert _{L^p({\mathbb {R}}^n;\,{\mathbb {R}}^n)}=0, \qquad \lim _{\beta \rightarrow \alpha ^-}\Vert \mathrm {div}^\beta \varphi -\mathrm {div}^\alpha \varphi \Vert _{L^p({\mathbb {R}}^n)}=0.\qquad \end{aligned}$$

#### Proof

Since clearly $f \in W^{\alpha , 1}({\mathbb {R}}^{n})$ for any $\alpha \in (0, 1)$, the first limit in (5.2) for the case $p=1$ follows from Lemma 5.1. Hence, we just need to prove the validity of the same limit for the case $p = + \infty$, since then the conclusion simply follows by an interpolation argument.

Let $\beta \in (0, \alpha )$ and $x \in {\mathbb {R}}^{n}$. We have

$$\begin{aligned} |\nabla ^{\alpha } f(x) - \nabla ^{\beta } f(x)|&\le |\mu _{n, \beta } - \mu _{n, \alpha }| \int _{{\mathbb {R}}^{n}} \frac{|f(x) - f(y)|}{|x - y|^{n + \alpha }} \, dy\\&\quad + \mu _{n, \beta } \int _{{\mathbb {R}}^{n}} \frac{|f(x) - f(y)|}{|x - y|^{n}} \, \bigg | \frac{1}{|x - y|^{\beta }} - \frac{1}{|x - y|^{\alpha }} \bigg | \, dy \\&= |\mu _{n, \beta } - \mu _{n, \alpha }| \int _{{\mathbb {R}}^{n}} \frac{|f(x + z) - f(x)|}{|z|^{n + \alpha }} \, dz \\&\quad + \mu _{n, \beta } \int _{{\mathbb {R}}^{n}} \frac{|f(x + z) - f(x)|}{|z|^{n}} \, \bigg | \frac{1}{|z|^{\beta }} - \frac{1}{|z|^{\alpha }} \bigg | \, dz. \end{aligned}$$

Since

$$\begin{aligned} \int _{{\mathbb {R}}^{n}} \frac{|f(x + z) - f(x)|}{|z|^{n + \alpha }} \, dz&\le \int _{\{ |z| \le 1 \}} \frac{{{\,\mathrm{Lip}\,}}(f)}{|z|^{n + \alpha - 1}} \, dz + \int _{\{ |z| > 1 \}} \frac{2 \Vert f\Vert _{L^{\infty }({\mathbb {R}}^{n})}}{|z|^{n + \alpha }} \, dz \\&\le n \omega _{n} \left( \frac{{{\,\mathrm{Lip}\,}}(f)}{1 - \alpha } + \frac{2 \Vert f\Vert _{L^{\infty }({\mathbb {R}}^{n})}}{\alpha } \right) \end{aligned}$$

and

$$\begin{aligned} \int _{{\mathbb {R}}^{n}} \frac{|f(x+ z) - f(z)|}{|z|^{n}} \, \bigg | \frac{1}{|z|^{\beta }} - \frac{1}{|z|^{\alpha }} \bigg | \, dz&\le \int _{\{ |z| \le 1 \}} \frac{{{\,\mathrm{Lip}\,}}(f)}{|z|^{n - 1}} \left( \frac{1}{|z|^{\alpha }} - \frac{1}{|z|^{\beta }} \right) dz \\&\quad + \int _{\{ |z| > 1 \}} \frac{2 \Vert f\Vert _{L^{\infty }({\mathbb {R}}^{n})}}{|z|^{n}} \bigg ( \frac{1}{|z|^{\beta }} - \frac{1}{|z|^{\alpha }} \bigg ) \, dz \\&\le (\alpha - \beta ) n \omega _{n} \, \bigg ( \frac{{{\,\mathrm{Lip}\,}}(f)}{(1 - \alpha )(1 - \beta )} + \frac{2 \Vert f\Vert _{L^{\infty }({\mathbb {R}}^{n})}}{\alpha \beta } \bigg ), \end{aligned}$$

for all $\beta \in \big (\frac{\alpha }{2}, \alpha \big )$ we obtain

$$\begin{aligned} \Vert \nabla ^{\alpha } f - \nabla ^{\beta } f\Vert _{L^{\infty }({\mathbb {R}}^{n};\, {\mathbb {R}}^{n})}\le c_{n, \alpha }\max \{{{\,\mathrm{Lip}\,}}(f),\Vert f\Vert _{L^\infty ({\mathbb {R}}^n)}\} \, \big ( |\mu _{n, \beta } - \mu _{n, \alpha }| + (\alpha - \beta )\big ), \end{aligned}$$

for some constant $c_{n, \alpha } > 0$ depending only on *n* and $\alpha$. Thus the conclusion follows since $\mu _{n,\beta }\rightarrow \mu _{n,\alpha }$ as $\beta \rightarrow \alpha ^-$. The second limit in (5.2) follows similarly and we leave the proof to the reader. $\square$

### Weak convergence of $\beta$-variation as $\beta \rightarrow \alpha ^-$

In Theorem 5.4 below, we prove the weak convergence of the $\beta$-variation as $\beta \rightarrow \alpha ^-$. The proof is very similar to those of Theorem 4.7 and Theorem 4.9 and is thus left to the reader.

#### Theorem 5.4

Let $\alpha \in (0,1)$. If $f \in BV^\alpha ({\mathbb {R}}^{n})$, then

$$\begin{aligned} D^\beta f\rightharpoonup D^\alpha f \quad \text {and} \quad |D^\beta f|\rightharpoonup |D^\alpha f| \quad \text {as } \beta \rightarrow \alpha ^-. \end{aligned}$$

Moreover, we have

5.3 $$\begin{aligned} \lim _{\beta \rightarrow \alpha ^-}|D^\beta f|({\mathbb {R}}^n)=|D^\alpha f|({\mathbb {R}}^n). \end{aligned}$$

### $\Gamma$-convergence of $\beta$-variation as $\beta \rightarrow \alpha ^-$

In this section, we study the $\Gamma$-convergence of the fractional $\beta$-variation as $\beta \rightarrow \alpha ^-$, partially extending the results obtained in Sect. 4.3.

We begin with the $\Gamma \text { -}\liminf$ inequality.

#### Theorem 5.5

($\Gamma \text { -}\liminf$ inequality for $\beta \rightarrow \alpha ^-$) Let $\alpha \in (0,1)$ and let $\Omega \subset {\mathbb {R}}^n$ be an open set. If $(f_\beta )_{\beta \in (0,\alpha )}\subset L^1({\mathbb {R}}^n)$ satisfies $f_\beta \rightarrow f$ in $L^1({\mathbb {R}}^n)$ as $\beta \rightarrow \alpha ^-$, then

5.4 $$\begin{aligned} |D^\alpha f|(\Omega )\le \liminf \limits _{\beta \rightarrow \alpha ^-}|D^\beta f_\beta |(\Omega ). \end{aligned}$$

#### Proof

We argue as in the proof of Theorem 4.13(ii). Let $\varphi \in C^\infty _c(\Omega ;{\mathbb {R}}^n)$ be such that $\Vert \varphi \Vert _{L^\infty (\Omega ;{\mathbb {R}}^n)}\le 1$. Let $U\subset {\mathbb {R}}^n$ be a bounded open set such that ${{\,\mathrm{supp}\,}}\varphi \subset U$. By (2.12), we can estimate

$$\begin{aligned}&\bigg |\int _{{\mathbb {R}}^n}f_\beta \,\mathrm {div}^\beta \varphi \,dx-\int _{{\mathbb {R}}^n}f\,\mathrm {div}^\alpha \varphi \,dx\bigg |\\&\quad \le \int _{{\mathbb {R}}^n}\Bigg |f_\beta -f\Bigg |\,|\mathrm {div}^\beta \varphi |\,dx +\int _{{\mathbb {R}}^n}|f|\,|\mathrm {div}^\beta \varphi -\mathrm {div}^\alpha \varphi |\,dx\\&\quad \le C_{n,\beta ,U}\Vert \mathrm {div}\varphi \Vert _{L^\infty ({\mathbb {R}}^n;\,{\mathbb {R}}^n)}\Vert f_\beta -f\Vert _{L^1({\mathbb {R}}^n)}+\int _{{\mathbb {R}}^n}|f|\,|\mathrm {div}^\beta \varphi -\mathrm {div}^\alpha \varphi |\,dx \end{aligned}$$

for all $\beta \in (0,\alpha )$. Since $\mathrm {div}^\beta \varphi \rightarrow \mathrm {div}^\alpha \varphi$ in $L^{\infty }({\mathbb {R}}^{n})$ as $\beta \rightarrow \alpha ^-$ by (5.2), we easily obtain

$$\begin{aligned} \lim _{\beta \rightarrow \alpha ^-}\int _{{\mathbb {R}}^n}|f|\,|\mathrm {div}^\beta \varphi -\mathrm {div}^\alpha \varphi |\,dx = 0. \end{aligned}$$

Hence, we get

$$\begin{aligned} \int _{{\mathbb {R}}^n}f\,\mathrm {div}^\alpha \varphi \,dx =\lim _{\beta \rightarrow \alpha ^-}\int _{{\mathbb {R}}^n}f_\beta \,\mathrm {div}^\beta \varphi \,dx \le \liminf _{\beta \rightarrow \alpha ^-}|D^\beta f_\beta |(\Omega ) \end{aligned}$$

and the conclusion follows. $\square$

We now pass to the $\Gamma \text { -}\limsup$ inequality.

#### Theorem 5.6

($\Gamma \text { -}\limsup$ inequality for $\beta \rightarrow \alpha ^-$) Let $\alpha \in (0,1)$ and let $\Omega \subset {\mathbb {R}}^n$ be an open set. If $f\in BV^\alpha ({\mathbb {R}}^n)$ and either $\Omega$ is bounded or $\Omega ={\mathbb {R}}^n$, then

5.5 $$\begin{aligned} \limsup _{\beta \rightarrow \alpha ^-}|D^\beta f|(\Omega )\le |D^\alpha f|({\overline{\Omega }}). \end{aligned}$$

#### Proof

We argue as in the proof of Theorem 4.14. By Theorem 5.4, we know that $|D^\beta f|\rightharpoonup |D^\alpha f|$ as $\beta \rightarrow \alpha ^-$. Thus, by [50,  Proposition 4.26] and (5.3), we get that

5.6 $$\begin{aligned} \limsup _{\beta \rightarrow \alpha ^-}|D^\beta f|(\Omega ) \le \limsup _{\beta \rightarrow \alpha ^-}|D^\beta f|( {}\overline{\Omega }) \le |D^\alpha f|( {}\overline{\Omega }) \end{aligned}$$

for any open set $\Omega \subset {\mathbb {R}}^n$ such that either $\Omega$ is bounded or $\Omega ={\mathbb {R}}^n$. $\square$

#### Corollary 5.7

($\Gamma (L^1)\text { -}\lim$ of variations in ${\mathbb {R}}^n$ as $\beta \rightarrow \alpha ^-$) Let $\alpha \in (0, 1)$. For every $f\in BV^{\alpha }({\mathbb {R}}^n)$, we have

$$\begin{aligned} \Gamma (L^1)\text { -}\lim _{\beta \rightarrow \alpha ^-}|D^\beta f|({\mathbb {R}}^{n}) =|D^{\alpha }f|({\mathbb {R}}^{n}). \end{aligned}$$

In particular, the constant sequence is a recovery sequence.

#### Proof

The result follows easily by combining (5.4) and (5.5) in the case $\Omega = {\mathbb {R}}^{n}$. $\square$

#### Remark 5.8

We recall that, by [27,  Theorem 3.25], $f \in BV^{\alpha }({\mathbb {R}}^{n})$ satisfies $|D^{\alpha } f| \ll {\mathscr {L}}^{n}$ if and only if $f \in S^{\alpha , 1}({\mathbb {R}}^{n})$. Therefore, if $f \in S^{\alpha , 1}({\mathbb {R}}^{n})$, then $|D^{\alpha }f|(\partial \Omega ) = 0$ for any bounded open set $\Omega \subset {\mathbb {R}}^n$ such that ${\mathscr {L}}^{n}(\partial \Omega ) = 0$ (for instance, $\Omega$ with Lipschitz boundary). Thus, we can actually obtain the $\Gamma$-convergence of the fractional $\beta$-variation as $\beta \rightarrow \alpha ^-$ on bounded open sets with Lipschitz boundary for any $f \in S^{\alpha , 1}({\mathbb {R}}^{n})$ too. Indeed, it is enough to combine (5.4) and (5.5) and then exploit the fact that $|D^{\alpha } f|(\partial \Omega )=0$ to get

$$\begin{aligned} \Gamma (L^1)\text { -}\lim _{\beta \rightarrow \alpha ^-}|D^\beta f|(\Omega ) =|D^{\alpha }f|(\Omega ) \end{aligned}$$

for any $$f \in S^{\alpha , 1}({\mathbb {R}}^{n})$$.

We were not able to find a reference for the analogue of Corollary 5.7 for the usual fractional Sobolev seminorms. For the sake of completeness, we state and prove it below for all $p\in [1,+\infty )$ on a general open set.

#### Theorem 5.9

($\Gamma (L^p)\text { -}\lim$ of $W^{\beta , p}$-seminorm as $\beta \rightarrow \alpha ^-$) Let $\Omega \subset {\mathbb {R}}^n$ be a non-empty open set, $\alpha \in (0, 1)$ and $p\in [1,+\infty )$. For every $f\in W^{\alpha , p}(\Omega )$, we have

$$\begin{aligned} \Gamma (L^p)\text { -}\lim _{\beta \rightarrow \alpha ^-} [f]_{W^{\beta ,p}(\Omega )} =[f]_{W^{\alpha ,p}(\Omega )}. \end{aligned}$$

In particular, the constant sequence is a recovery sequence.

#### Proof

Let $(f_\beta )_{\beta \in (0,\alpha )}\subset L^p(\Omega )$ be such that $f_\beta \rightarrow f$ in $L^p(\Omega )$ as $\beta \rightarrow \alpha ^-$. Let $(\beta _k) \subset (0, \alpha )$ be such that $\beta _k\rightarrow \alpha$ as $k\rightarrow +\infty$ and

$$\begin{aligned} \liminf _{\beta \rightarrow \alpha ^-} [f_{\beta }]_{W^{\beta ,p}(\Omega ;\,{\mathbb {R}}^m)} = \lim _{k \rightarrow + \infty } [f_{\beta _k}]_{W^{\beta _k,p}(\Omega ;\,{\mathbb {R}}^m)}. \end{aligned}$$

Up to extract a further subsequence, we can assume that $f_{\beta _k}(x) \rightarrow f(x)$ as $k \rightarrow + \infty$ for a.e. $x \in \Omega$. Then we can estimate

$$\begin{aligned} \lim _{k \rightarrow + \infty } \int _\Omega \int _\Omega \frac{|f_{\beta _k}(x)-f_{\beta _k}(y)|^p}{|x-y|^{n+p\beta _k}}\,dx\,dy&\ge \int _\Omega \int _\Omega \liminf _{k \rightarrow + \infty } \frac{|f_{\beta _k}(x)-f_{\beta _k}(y)|^p}{|x-y|^{n+p\beta _k}}\,dx\,dy \\&\ge \int _\Omega \int _\Omega \frac{|f(x)-f(y)|^p}{|x-y|^{n+p\alpha }}\,dx\,dy \end{aligned}$$

by Fatou’s Lemma. We thus get that

$$\begin{aligned} \Gamma (L^p)\text { -}\liminf _{\beta \rightarrow \alpha ^-} \, [f]_{W^{\beta ,p}(\Omega )} \ge [f]_{W^{\alpha ,p}(\Omega )}. \end{aligned}$$

Since

$$\begin{aligned} \lim _{\beta \rightarrow \alpha ^-} \int _\Omega \int _\Omega \frac{|f(x)-f(y)|^p}{|x-y|^{n+p\beta }}\,dx\,dy&= \lim _{\beta \rightarrow \alpha ^-} \int _\Omega \int _\Omega \frac{|f(x)-f(y)|^p}{|x-y|^{n+p\beta }}\,\chi _{\{|x-y|<1\}}\,dx\,dy \\&\quad + \lim _{\beta \rightarrow \alpha ^-} \int _\Omega \int _\Omega \frac{|f(x)-f(y)|^p}{|x-y|^{n+p\beta }}\,\chi _{\{|x-y|>1\}}\,dx\,dy \\&= \int _\Omega \int _\Omega \frac{|f(x)-f(y)|^p}{|x-y|^{n+p\alpha }}\,dx\,dy \end{aligned}$$

by the Monotone Convergence Theorem, we also have that

$$\begin{aligned} \Gamma (L^p)\text { -}\limsup _{\beta \rightarrow \alpha ^-} \,[f]_{W^{\beta ,p}(\Omega )} \le [f]_{W^{\alpha ,p}(\Omega )} \end{aligned}$$

and the conclusion immediately follows. $\square$

## Appendix A. Truncation and approximation of *BV* functions

In this appendix, we deal with two results on *BV* functions and sets with locally finite perimeter. These results are well known to experts, but we decided to state and prove them here because either we were not able to find them formulated in the exact form we needed or the results available in the literature were not proved in full correctness (see Remark A.5 below).

### A.1 Truncation of *BV* functions

Following [4,  Section 3.6] and [34,  Section 5.9], given $f\in L^1_{{{\,\mathrm{loc}\,}}}({\mathbb {R}}^n)$, we define its precise representative $f^\star :{\mathbb {R}}^n\rightarrow [-\infty ,+\infty ]$ as

A.1 $$\begin{aligned} f^\star (x):=\lim _{r\rightarrow 0^+}\frac{1}{\omega _n r^n}\int _{B_r(x)} f(y)\,dy, \quad x\in {\mathbb {R}}^n, \end{aligned}$$

if the limit exists, otherwise we let $f^\star (x)=0$ by convention.

#### Theorem A.1

(Truncation of *BV* functions) If $f\in BV_{{{\,\mathrm{loc}\,}}}({\mathbb {R}}^n)$, then

A.2 $$\begin{aligned} f\chi _{B_r}\in BV({\mathbb {R}}^n), \ \text {with}\ D(f\chi _{B_r})=\chi _{B_r}^\star Df+f^\star D\chi _{B_r}, \end{aligned}$$

for ${\mathscr {L}}^{1}$-a.e. $r>0$. If, in addition, $f\in L^\infty ({\mathbb {R}}^n)$, then (A.2) holds for all $r>0$.

#### Proof

Fix $\varphi \in C^\infty _c({\mathbb {R}}^n;{\mathbb {R}}^n)$ and let $U\subset {\mathbb {R}}^n$ be a bounded open set such that ${{\,\mathrm{supp}\,}}(\varphi )\subset U$. Let $(\varrho _\varepsilon )_{\varepsilon >0}\subset C^\infty _c({\mathbb {R}}^n)$ be a family of standard mollifiers as in [27,  Section 3.3] and set $f_\varepsilon :=f*\varrho _\varepsilon$ for all $\varepsilon >0$. Note that ${{\,\mathrm{supp}\,}}\big (\varrho _\varepsilon *(\chi _{B_r}\varphi )\big )\subset U$ and ${{\,\mathrm{supp}\,}}\big (\varrho _\varepsilon *(\chi _{B_r}\mathrm {div}\varphi )\big )\subset U$ for all $\varepsilon >0$ sufficiently small and for all $r>0$. Given $r>0$, by Leibniz’s rule and Fubini’s Theorem, we have

A.3 $$\begin{aligned} \begin{aligned} \int _{{\mathbb {R}}^n}f_\varepsilon \chi _{B_r}\,\mathrm {div}\varphi \,dx&=\int _{{\mathbb {R}}^n}\chi _{B_r}\mathrm {div}(f_\varepsilon \varphi )\,dx -\int _{{\mathbb {R}}^n}\chi _{B_r}\varphi \cdot \nabla f_\varepsilon \,dx\\&=-\int _{{\mathbb {R}}^n}f_\varepsilon \varphi \cdot \,dD\chi _{B_r} -\int _{{\mathbb {R}}^n}\varrho _\varepsilon *(\chi _{B_r}\varphi )\cdot dDf. \end{aligned} \end{aligned}$$

Since $f_\varepsilon \rightarrow f$ a.e. in ${\mathbb {R}}^n$ as $\varepsilon \rightarrow 0^+$ and

$$\begin{aligned} |f|\,\varrho _\varepsilon *(\chi _{B_r}|\mathrm {div}\varphi |)\le |f|\chi _U\Vert \mathrm {div}\varphi \Vert _{L^\infty ({\mathbb {R}}^n)}\in L^1({\mathbb {R}}^n) \end{aligned}$$

for all $\varepsilon >0$, by Lebesgue’s Dominated Convergence Theorem we have

$$\begin{aligned} \lim _{\varepsilon \rightarrow 0^+}\int _{{\mathbb {R}}^n}f_\varepsilon \chi _{B_r}\,\mathrm {div}\varphi \,dx =\int _{{\mathbb {R}}^n}f\chi _{B_r}\,\mathrm {div}\varphi \,dx \end{aligned}$$

for all $r>0$. Thus, since $\varrho _\varepsilon *(\chi _{B_r}\varphi )\rightarrow \chi _{B_r}^\star \varphi$ pointwise in ${\mathbb {R}}^n$ as $\varepsilon \rightarrow 0^+$ and

$$\begin{aligned} |\varrho _\varepsilon *(\chi _{B_r}\varphi )|\le \Vert \varphi \Vert _{L^\infty ({\mathbb {R}}^n;\,{\mathbb {R}}^n)}\chi _U\in L^1({\mathbb {R}}^n,|Df|) \end{aligned}$$

for all $\varepsilon >0$ sufficiently small, again by Lebesgue’s Dominated Convergence Theorem we have

$$\begin{aligned} \lim _{\varepsilon \rightarrow 0^+}\int _{{\mathbb {R}}^n}\varrho _\varepsilon *(\chi _{B_r}\varphi )\cdot dDf =\int _{{\mathbb {R}}^n}\chi _{B_r}^\star \varphi \cdot dDf \end{aligned}$$

for all $r>0$. Now, by [4,  Theorem 3.78 and Corollary 3.80], we know that $f_\varepsilon \rightarrow f^\star$ ${\mathscr {H}}^{n-1}$-a.e. in ${\mathbb {R}}^n$ as $\varepsilon \rightarrow 0^+$. As a consequence, given any $r>0$, we get that $f_\varepsilon \rightarrow f^\star$ $|D\chi _{B_r}|$-a.e. in ${\mathbb {R}}^n$ as $\varepsilon \rightarrow 0^+$. Thus, if $f\in L^\infty ({\mathbb {R}}^n)$, then

$$\begin{aligned} |f_\varepsilon \varphi |\le \Vert f\Vert _{L^\infty ({\mathbb {R}}^n)}|\varphi |\in L^1({\mathbb {R}}^n,|D\chi _{B_r}|) \end{aligned}$$

for all $\varepsilon >0$ and so, again by Lebesgue’s Dominated Convergence Theorem, we have

$$\begin{aligned} \lim _{\varepsilon \rightarrow 0^+}\int _{{\mathbb {R}}^n}f_\varepsilon \varphi \cdot \,dD\chi _{B_r} =\int _{{\mathbb {R}}^n}f^\star \varphi \cdot \,dD\chi _{B_r} \end{aligned}$$

for all $r>0$. Therefore, if $f\in L^\infty ({\mathbb {R}}^n)$, then we can pass to the limit as $\varepsilon \rightarrow 0^+$ in (A.3) and get

$$\begin{aligned} \int _{{\mathbb {R}}^n}f\chi _{B_r}\,\mathrm {div}\varphi \,dx =-\int _{{\mathbb {R}}^n}f^\star \varphi \cdot \,dD\chi _{B_r} -\int _{{\mathbb {R}}^n}\chi _{B_r}^\star \varphi \cdot dDf \end{aligned}$$

for all $\varphi \in C^\infty _c({\mathbb {R}}^n;{\mathbb {R}}^n)$ and for all $r>0$. Since $\Vert f^\star \Vert _{L^\infty ({\mathbb {R}}^n)}\le \Vert f\Vert _{L^\infty ({\mathbb {R}}^n)}$, this proves (A.2) for all $r>0$.

If *f* is not necessarily bounded, then we argue as follows. We start by observing that, since

$$\begin{aligned} \int _0^R\int _{\partial B_r}|f^\star |\,d{\mathscr {H}}^{n-1}\,dr =\int _{B_R}|f^\star |\,dx = \int _{B_R}|f|\,dx <+\infty , \end{aligned}$$

the set

$$\begin{aligned} W:=\Bigg \{r>0 : \int _{\partial B_r}|f^\star |\,d{\mathscr {H}}^{n-1}\,dr<+\infty \Bigg \} \end{aligned}$$

satisfies ${\mathscr {L}}^{1}((0,+\infty )\setminus W)=0$. Without loss of generality, assume that $\Vert \varphi \Vert _{L^\infty ({\mathbb {R}}^n;\,{\mathbb {R}}^n)}\le 1$. Hence, for all $r \in W$, we can estimate

A.4 $$\begin{aligned} \Bigg |\int _{{\mathbb {R}}^n}f_\varepsilon \varphi \cdot \,dD\chi _{B_r} -\int _{{\mathbb {R}}^n}f^\star \varphi \cdot \,dD\chi _{B_r}\Bigg | \le \int _{\partial B_r}|f_\varepsilon -f^\star |\,d{\mathscr {H}}^{n-1}. \end{aligned}$$

Given any $R > 0$, by Fatou’s Lemma we thus get that

$$\begin{aligned}&\int _0^R\liminf _{\varepsilon \rightarrow 0^+}\,\Bigg |\int _{{\mathbb {R}}^n}f_\varepsilon \varphi \cdot \,dD\chi _{B_r} -\int _{{\mathbb {R}}^n}f^\star \varphi \cdot \,dD\chi _{B_r}\Bigg |\,dr\\&\quad \le \int _0^R\liminf _{\varepsilon \rightarrow 0^+}\int _{\partial B_r}|f_\varepsilon -f^\star |\,d{\mathscr {H}}^{n-1}\,dr\\&\quad \le \liminf _{\varepsilon \rightarrow 0^+}\int _0^R\int _{\partial B_r}|f_\varepsilon -f^\star |\,d{\mathscr {H}}^{n-1}\,dr\\&\quad =\lim _{\varepsilon \rightarrow 0^+}\int _{B_R}|f_\varepsilon -f^\star |\,dx =0. \end{aligned}$$

Hence, the set

A.5 $$\begin{aligned} Z:=\Bigg \{r>0 : \liminf _{\varepsilon \rightarrow 0^+}\int _{\partial B_r}|f_\varepsilon -f^\star |\,d{\mathscr {H}}^{n-1}=0\Bigg \} \end{aligned}$$

satisfies ${\mathscr {L}}^{1}((0,+\infty )\setminus Z)=0$ and clearly does not depend on the choice of $\varphi$. Now fix $r\in Z \cap W$ and let $(\varepsilon _k)_{k\in {\mathbb {N}}}$ be any sequence realising the $\liminf$ in (A.5). By (A.4), we thus get

$$\begin{aligned} \lim _{k\rightarrow +\infty }\int _{{\mathbb {R}}^n}f_{\varepsilon _k}\varphi \cdot \,dD\chi _{B_r} =\int _{{\mathbb {R}}^n}f^\star \varphi \cdot \,dD\chi _{B_r} \end{aligned}$$

uniformly for all $\varphi$ satisfying $\Vert \varphi \Vert _{L^\infty ({\mathbb {R}}^n;\,{\mathbb {R}}^n)}\le 1$. Passing to the limit along the sequence $(\varepsilon _k)_{k\in {\mathbb {N}}}$ as $k\rightarrow +\infty$ in (A.3), we get that

$$\begin{aligned} \int _{{\mathbb {R}}^n}f\chi _{B_r}\,\mathrm {div}\varphi \,dx =-\int _{{\mathbb {R}}^n}f^\star \varphi \cdot \,dD\chi _{B_r} -\int _{{\mathbb {R}}^n}\chi _{B_r}^\star \varphi \cdot dDf \end{aligned}$$

for all $\varphi \in C^\infty _c({\mathbb {R}}^n;{\mathbb {R}}^n)$ with $\Vert \varphi \Vert _{L^\infty ({\mathbb {R}}^n;\,{\mathbb {R}}^n)}\le 1$. Thus (A.2) follows for all $r\in W\cap Z$ and the proof is concluded. $\square$

### A.2 Approximation by sets with polyhedral boundary

In this section we state and prove standard approximation results for sets with finite perimeter or, more generally, $BV_{{{\,\mathrm{loc}\,}}}({\mathbb {R}}^n)$ functions, in a sufficiently regular bounded open set.

We need the following two preliminary lemmas.

#### Lemma A.2

Let $V,W\subset {\mathbb {S}}^{n-1}$, with *V* finite and *W* at most countable. For any $\varepsilon >0$, there exists ${\mathcal {R}}\in \mathrm {SO}(n)$ with $|{\mathcal {R}}-{\mathcal {I}}|<\varepsilon$, where ${\mathcal {I}}$ is the identity matrix, such that ${\mathcal {R}}(V)\cap W=\varnothing$.

#### Proof

Let $N\in {\mathbb {N}}$ be such that $V=\Bigg \{v_i\in {\mathbb {S}}^{n-1} : i=1,\dots ,N\Bigg \}$. We divide the proof in two steps.

*Step 1*. Assume that *W* is finite and set $A_i:=\Bigg \{{\mathcal {R}}\in \mathrm {SO}(n) : {\mathcal {R}}(v_i)\notin W\Bigg \}$ for all $i=1,\dots ,N$. We now claim that $A_i$ of $\mathrm {SO}(n)$ for all $i=1,\dots ,N$. Indeed, given any $i=1,\dots ,N$, since *W* is finite, the set $A_i^c=\mathrm {SO}(n)\setminus A_i$ is closed in $\mathrm {SO}(n)$. Moreover, we claim that $\mathrm {int}(A_i^c) = \varnothing$. Indeed, by contradiction, let us assume that $\mathrm {int}(A_i^c)\ne \varnothing$. Then there exist $\varepsilon >0$ and ${\mathcal {R}}\in A_i^c$ such that any ${\mathcal {S}}\in \mathrm {SO}(n)$ with $|{\mathcal {S}}-{\mathcal {R}}|<\varepsilon$ satisfies ${\mathcal {S}}\in A_i^c$. Now let

and define ${\mathcal {S}}_\vartheta := {\mathcal {Q}}_\vartheta \,\mathcal R\in \mathrm {SO}(n)$ for all $\vartheta \in [0,2\pi )$. Since

$$\begin{aligned} |{\mathcal {S}}_\vartheta - {\mathcal {R}}| = |({\mathcal {Q}}_\vartheta - \mathcal I)\,{\mathcal {R}}| \le |{\mathcal {Q}}_\vartheta - {\mathcal {I}}|\,|{\mathcal {R}}| < \varepsilon \end{aligned}$$

for all $\vartheta \in [0,\delta ]$ for some $\delta >0$ depending only on $\varepsilon$ and ${\mathcal {R}}$, we get that ${\mathcal {S}}_\vartheta \in A_i^c$ for all $\vartheta \in [0,\delta ]$. Therefore ${\mathcal {S}}_\vartheta (v_i)\in W$ for all $\vartheta \in [0,\delta ]$, in contrast with the fact that *W* is finite. Thus, $A_i$ is an open and dense subset of $\mathrm {SO}(n)$ for all $i=1,\dots ,N$, and so also the set

$$\begin{aligned} A^W:=\bigcap _{i=1}^N A_i=\{{\mathcal {R}}\in \mathrm {SO}(n) : {\mathcal {R}}(v_i)\notin W\ \forall i=1,\dots ,N\} \end{aligned}$$

is an open and dense subset of $\mathrm {SO}(n)$. The result is thus proved for any finite set *W*.

*Step 2*. Now assume that *W* is countable, $W=\{w_k\in {\mathbb {S}}^{n-1} : k\in {\mathbb {N}}\}$. For all $M\in {\mathbb {N}}$, set $W_M:=\{w_k\in W : k\le M\}$. By Step 1, we know that $A^{W_M}$ is an open and dense subset of $\mathrm {SO}(n)$ for all $M\in {\mathbb {N}}$. Since $\mathrm {SO}(n)\subset {\mathbb {R}}^{n^2}$ is compact, by Baire’s Theorem $A:=\bigcap _{M\in {\mathbb {N}}} A^{W_M}$ is a dense subset of $\mathrm {SO}(n)$. This concludes the proof. $\square$

Since $\det :\mathrm {GL}(n)\rightarrow {\mathbb {R}}$ is a continuous map, there exists a dimensional constant $\delta _n\in (0,1)$ such that $\det {\mathcal {R}}\ge \frac{1}{2}$ for all ${\mathcal {R}}\in \mathrm {GL}(n)$ with $|{\mathcal {R}}-{\mathcal {I}}|<\delta _n$.

#### Lemma A.3

Let $\varepsilon \in (0,\delta _n)$ and let $E\subset {\mathbb {R}}^n$ be a bounded set with $P(E)<+\infty$. If ${\mathcal {R}}\in \mathrm {SO}(n)$ satisfies $|{\mathcal {R}}-{\mathcal {I}}|<\varepsilon$, then

$$\begin{aligned} |{\mathcal {R}}(E)\bigtriangleup E|\le 2\varepsilon r_E \, P(E), \end{aligned}$$

where $$r_E:=\sup \{r>0 : |E\setminus B_r|>0\}$$.

#### Proof

We divide the proof in two steps.

*Step 1*. Let $r>0$ and let $f\in C^\infty _c({\mathbb {R}}^n)$. Setting ${\mathcal {R}}_t:=(1-t){\mathcal {I}}+t{\mathcal {R}}$ for all $t\in [0,1]$, we can estimate

$$\begin{aligned} \int _{B_r} \int _{B_r}|f({\mathcal {R}}(x))-f(x)|\,dx&=\int _{B_r}\Bigg |\int _0^1{\nabla f(\mathcal {R}_t(x)) \cdot (\mathcal {R}(x)-x)}\,dt\Bigg |\,dx\\&\le |{\mathcal {R}}-{\mathcal {I}}|\,r\int _0^1\int _{B_r}|\nabla f({\mathcal {R}}_t(x))|\,dx\,dt. \end{aligned}$$

Since $|{\mathcal {R}}_t-{\mathcal {I}}|=t|{\mathcal {R}}-{\mathcal {I}}|<t\varepsilon <\delta _n$ for all $t\in [0,1]$, ${\mathcal {R}}_t$ is invertible with $\det ({\mathcal {R}}_t^{-1})\le 2$ for all $t\in [0,1]$. Hence we can estimate

$$\begin{aligned} \int _{B_r}|\nabla f({\mathcal {R}}_t(x))|\,dx =\int _{{\mathcal {R}}_t(B_r)}|\nabla f(y)|\,|\det ({\mathcal {R}}^{-1}_t)|\,dy \le 2\int _{{\mathbb {R}}^n}|\nabla f(y)|\,dy, \end{aligned}$$

so that

A.6 $$\begin{aligned} \int _{B_r}|f({\mathcal {R}}(x))-f(x)|\,dx \le 2\varepsilon r\Vert \nabla f\Vert _{L^{1}({\mathbb {R}}^n; {\mathbb {R}}^{n})}. \end{aligned}$$

*Step 2*. Since $\chi _E\in BV({\mathbb {R}}^n)$, by combining [34,  Theorem 5.3] with a standard cut-off approximation argument, we find $(f_k)_{k\in {\mathbb {N}}}\subset C^\infty _c({\mathbb {R}}^n)$ such that $f_k\rightarrow \chi _E$ pointwise a.e. in ${\mathbb {R}}^n$ and $|\nabla f_k|({\mathbb {R}}^n)\rightarrow P(E)$ as $k\rightarrow +\infty$. Given any $r>0$, by (A.6) in Step 1 we have

$$\begin{aligned} \int _{B_r}|f_k({\mathcal {R}}(x))-f_k(x)|\,dx \le 2\varepsilon r\Vert \nabla f_k\Vert _{L^{1}({\mathbb {R}}^n; {\mathbb {R}}^{n})} \end{aligned}$$

for all $k\in {\mathbb {N}}$. Passing to the limit as $k\rightarrow +\infty$, by Fatou’s Lemma we get that

$$\begin{aligned} |({\mathcal {R}}(E)\bigtriangleup E)\cap B_r|\le 2\varepsilon r\, P(E). \end{aligned}$$

Since $E\subset B_{r_E}$ up to ${\mathscr {L}}^{n}$-negligible sets, also ${\mathcal {R}}(E)\subset B_{r_E}$ up to ${\mathscr {L}}^{n}$-negligible sets. Thus we can choose $r=r_E$ and the proof is complete. $\square$

We are now ready to prove the main approximation result, see also [3,  Proposition 15].

#### Theorem A.4

Let $\Omega \subset {\mathbb {R}}^n$ be a bounded open set with Lipschitz boundary and let $E\subset {\mathbb {R}}^n$ be a measurable set such that $P(E;\Omega )<+\infty$. There exists a sequence $(E_k)_{k\in {\mathbb {N}}}$ of bounded open sets with polyhedral boundary such that

A.7 $$\begin{aligned} P(E_k;\partial \Omega )=0 \end{aligned}$$

for all $k\in {\mathbb {N}}$ and

A.8 $$\begin{aligned} \chi _{E_k}\rightarrow \chi _E\ \text {in } L^1_{{{\,\mathrm{loc}\,}}}({\mathbb {R}}^n) \quad \text {and}\quad P(E_k;\Omega )\rightarrow P(E;\Omega ) \end{aligned}$$

as $$k\rightarrow +\infty $$.

#### Proof

We divide the proof in four steps.

*Step 1: cut-off*. Since $\Omega$ is bounded, we find $R_0>0$ such that ${\overline{\Omega }}\subset B_{R_0}$. Let us define $R_k=R_0+k$ and

$$\begin{aligned} C_k:=\Bigg \{x\in \Omega ^c : {{\,\mathrm{dist}\,}}(x,\partial \Omega )\le \frac{1}{k}\Bigg \} \end{aligned}$$

for all $k\in {\mathbb {N}}$. We set $E^1_k:=E\cap B_{R_k}\cap C_k^c$ for all $k\in {\mathbb {N}}$. Note that $E_k^1$ is a bounded measurable set such that

$$\begin{aligned} \chi _{E_k^1}\rightarrow \chi _E\ \text {in } L^1_{{{\,\mathrm{loc}\,}}}({\mathbb {R}}^n) \text { as } k\rightarrow +\infty \end{aligned}$$

and

$$\begin{aligned} P(E_k^1;\Omega )=P(E;\Omega )\ \text {for all } k\in {\mathbb {N}}. \end{aligned}$$

*Step 2: extension*. Let us define

$$\begin{aligned} A_k:=\Bigg \{x\in {\mathbb {R}}^n : {{\,\mathrm{dist}\,}}(x,\Omega )<\frac{1}{4k}\Bigg \} \end{aligned}$$

for all $k\in {\mathbb {N}}$. Since $\chi _{E_k^1\cap \Omega }\in BV(\Omega )$ for all $k\in {\mathbb {N}}$, by [4,  Definition 3.20 and Proposition 3.21] there exists a sequence $(v_k)_{k\in {\mathbb {N}}}\subset BV({\mathbb {R}}^n)$ such that

$$\begin{aligned} v_k=0 \text { a.e. in } A^c_k, \quad v_k=\chi _{E_k^1} \text { in } \Omega , \quad |Dv_k|(\partial \Omega )=0 \end{aligned}$$

for all $k\in {\mathbb {N}}$. Let us define $F^t_k:=\{v_k>t\}$ for all $t\in (0,1)$. Given $k\in {\mathbb {N}}$, by the coarea formula [4,  Theorem 3.40], for a.e. $t\in (0,1)$ the set $F_k^t$ has finite perimeter in ${\mathbb {R}}^n$ and satisfies

$$\begin{aligned} F_k^t\subset A_k, \quad F_k^t\cap \Omega =E_k^1\cap \Omega , \quad P(F_k^t;\partial \Omega )=0 \end{aligned}$$

for all $k\in {\mathbb {N}}$. We choose any such $t_k\in (0,1)$ for each $k\in {\mathbb {N}}$ and define $E_k^2:=E_k^1\cup F_k^{t_k}$ for all $k\in {\mathbb {N}}$. Note that $E_k^2$ is a bounded set with finite perimeter in ${\mathbb {R}}^n$ such that

$$\begin{aligned} \chi _{E_k^2}\rightarrow \chi _E\ \text {in } L^1_{{{\,\mathrm{loc}\,}}}({\mathbb {R}}^n) \text { as } k\rightarrow +\infty \end{aligned}$$

and

$$\begin{aligned} P(E_k^2;\Omega )=P(E;\Omega )\ \quad \text {and}\quad P(E_k^2;\partial \Omega )=0 \quad \text {for all } k\in {\mathbb {N}}. \end{aligned}$$

*Step 3: approximation*. Let us define

$$\begin{aligned} D_k:=\Bigg \{x\in \Omega ^c : {{\,\mathrm{dist}\,}}(x,\partial \Omega )\in \left[ \frac{1}{4k},\frac{3}{4k}\right] \Bigg \} \end{aligned}$$

for all $k\in {\mathbb {N}}$. First arguing as in the first part of the proof of [50,  Theorem 13.8] taking [50,  Remark 13.13] into account, and then performing a standard diagonal argument, we find a sequence of bounded open sets $(E_k^3)_{k\in {\mathbb {N}}}$ with polyhedral boundary such that

$$\begin{aligned} E^3_k\subset D_k^c\ \text {for all } k\in {\mathbb {N}}\end{aligned}$$

and

$$\begin{aligned} \chi _{E_k^3}\rightarrow \chi _E\ \text {in } L^1_{{{\,\mathrm{loc}\,}}}({\mathbb {R}}^n), \quad P(E_k^3;\Omega )\rightarrow P(E;\Omega ) \quad \text {and}\quad P(E_k^3;\partial \Omega )\rightarrow 0 \end{aligned}$$

as $k\rightarrow +\infty$. If there exists a subsequence $(E_{k_j}^3)_{j\in {\mathbb {N}}}$ such that $P(E_{k_j}^3;\partial \Omega )=0$ for all $j\in {\mathbb {N}}$, then we can set $E_j:=E_{k_j}$ for all $j\in {\mathbb {N}}$ and the proof is concluded. If this is not the case, then we need to proceed with the next last step.

*Step 4: rotation*. We now argue as in the last part of the proof of [3,  Proposition 15]. Fix $k\in {\mathbb {N}}$ and assume $P(E^3_k;\partial \Omega )>0$. Since $E_k^3$ has polyhedral boundary, we have ${\mathscr {H}}^{n-1}(\partial E^3_k\cap \partial \Omega )>0$ if and only if there exist $\nu \in {\mathbb {S}}^{n-1}$ and $U\subset {\mathscr {F}}\Omega$ such that ${\mathscr {H}}^{n-1}(U)>0$, $\nu _\Omega (x)=\nu$ for all $x\in U$ and $U\subset \partial H$ for some (affine) half-space *H* satisfying $\nu _H=\nu$. Since $P(\Omega )={\mathscr {H}}^{n-1}(\partial \Omega )<+\infty$, the set

$$\begin{aligned} W&:=\{\nu \in {\mathbb {S}}^{n-1} : {\mathscr {H}}^{n-1}\left( \{x\in \partial \Omega : \nu _\Omega (x)=\nu \}\right)>0\}\\&=\bigcup _{h\in {\mathbb {N}}}\Bigg \{\nu \in {\mathbb {S}}^{n-1} : \tfrac{P(\Omega )}{h}\ge {\mathscr {H}}^{n-1}\left( \{x\in \partial \Omega : \nu _\Omega (x)=\nu \}\right) >\tfrac{P(\Omega )}{h+1})\Bigg \} \end{aligned}$$

is at most countable. Since $E_k^3$ has polyhedral boundary, the set

$$\begin{aligned} V_k:=\{\nu \in {\mathbb {S}}^{n-1} : {\mathscr {H}}^{n-1}\left( \{x\in \partial E^3_k : \nu _{E^3_k}(x)=\nu \}\right) >0\} \end{aligned}$$

is finite. By Lemma A.2, given $\varepsilon _k>0$, there exists ${\mathcal {R}}_k\in \mathrm {SO}(n)$ with $|{\mathcal {R}}_k-{\mathcal {I}}|<\varepsilon _k$ such that ${\mathcal {R}}_k(V_k)\cap W=\varnothing$. Hence the set $E^4_k:={\mathcal {R}}_k(E^3_k)$ must satisfy $P(E^4_k;\partial \Omega )=0$. By Lemma A.3, we can choose $\varepsilon _k>0$ sufficiently small in order to ensure that $|E^4_k\bigtriangleup E^3_k|<\frac{1}{k}$. Now choose $\eta _k\in \left( 0,\frac{1}{2k}\right)$ such that $P(E^3_k;Q_k)\le 2P(E^3_k;\partial \Omega )$, where

$$\begin{aligned} Q_k:=\{x\in {\mathbb {R}}^n : {{\,\mathrm{dist}\,}}(x,\partial \Omega )<\eta _k\}. \end{aligned}$$

Since $\Omega$ is bounded, possibly choosing $\varepsilon _k>0$ even smaller, we can also ensure that $\Omega \bigtriangleup {\mathcal {R}}^{-1}(\Omega )\subset Q_k$. Hence we can estimate

$$\begin{aligned} |P(E^4_k;\Omega )-P(E^3_k;\Omega )|&=|{\mathscr {H}}^{n-1}(\partial E^3_k\cap {\mathcal {R}}^{-1}(\Omega ))-{\mathscr {H}}^{n-1}(\partial E^3_k\cap \Omega )|\\&\le {\mathscr {H}}^{n-1}\big (\partial E^3_k\cap (\Omega \bigtriangleup {\mathcal {R}}^{-1}(\Omega ))\big )\\&\le {\mathscr {H}}^{n-1}(\partial E^3_k\cap Q_k). \end{aligned}$$

We can thus set $E_k:=E^4_k$ for all $k\in {\mathbb {N}}$ and the proof is complete. $\square$

#### Remark A.5

*(A minor gap in the proof of* [3,  Proposition 15]*)* We warn the reader that the cut-off and the extension steps presented above were not mentioned in the proof of [3,  Proposition 15], although they are unavoidable for the correct implementation of the rotation argument in the last step. Indeed, in general, one cannot expect the existence of a rotation ${\mathcal {R}}\in \mathrm {SO}(n)$ arbitrarily close to the identity map such that $P({\mathcal {R}}(E);\partial \Omega )=0$ and, at the same time, the difference between $P({\mathcal {R}}(E);\Omega )$ and $P(E;\Omega )$ is small. For example, one can consider $n=2$,

$$\begin{aligned} \Omega =\{(x_1,x_2)\in A : x_1^2+x_2^2<25\} \end{aligned}$$

and

$$\begin{aligned} E=\{(x_1,x_2)\in A : 1<x_1^2+x_2^2<4\} \cup \{(x_1,x_2)\in A^c : 9<x_1^2+x_2^2<16\}, \end{aligned}$$

where $A=\{(x_1,x_2)\in {\mathbb {R}}^2 : x_1>0,\ x_2>0\}$. In this case, for any rotation ${\mathcal {R}}\in \mathrm {SO}(2)$ arbitrarily close to the identity map, we have $P({\mathcal {R}}(E);\Omega )>2+P(E;\Omega )$.

We conclude this section with the following result, establishing an approximation of $BV_{{{\,\mathrm{loc}\,}}}$ functions similar to that given in Theorem A.4.

#### Theorem A.6

Let $\Omega \subset {\mathbb {R}}^n$ be a bounded open set with Lipschitz boundary and let $f\in BV_{{{\,\mathrm{loc}\,}}}({\mathbb {R}}^n)$. There exists $(f_k)_{k\in {\mathbb {N}}}\subset BV({\mathbb {R}}^n)$ such that

$$\begin{aligned} |Df_k|(\partial \Omega )=0 \end{aligned}$$

for all $k\in {\mathbb {N}}$ and

$$\begin{aligned} f_k\rightarrow f\ \text {in } L^1_{{{\,\mathrm{loc}\,}}}({\mathbb {R}}^n) \quad \text {and}\quad |Df_k|(\Omega )\rightarrow |Df|(\Omega ) \end{aligned}$$

as $k\rightarrow +\infty$. If, in addition, $f\in L^1({\mathbb {R}}^n)$, then $f_k\rightarrow f$ in $L^1({\mathbb {R}}^n)$ as $k\rightarrow +\infty$.

#### Proof

We argue as in the proof of Theorem A.4, in two steps.

*Step 1: cut-off at infinity*. Since $\Omega$ is bounded, we find $R_0>0$ such that ${\overline{\Omega }}\subset B_{R_0}$. Given $(R_k)_k\subset (R_0,+\infty )$, we set $g_k:=f\chi _{B_{R_k}}$ for all $k\in {\mathbb {N}}$. By Theorem A.1, we have $g_k\in BV({\mathbb {R}}^n)$ for a suitable choice of the sequence $(R_k)_{k\in {\mathbb {N}}}$, with $|Dg_k|(\Omega )=|Df|(\Omega )$ for all $k\in {\mathbb {N}}$ and $g_k\rightarrow f$ in $L^1_{{{\,\mathrm{loc}\,}}}({\mathbb {R}}^n)$ as $k\rightarrow +\infty$. If, in addition, $f\in L^1({\mathbb {R}}^n)$, then $g_k\rightarrow f$ in $L^1({\mathbb {R}}^n)$ as $k\rightarrow +\infty$.

*Step 2: extension and cut-off near* $\Omega$. Let us define

$$\begin{aligned} A_k:=\Bigg \{x\in {\mathbb {R}}^n : {{\,\mathrm{dist}\,}}(x,\Omega )<\frac{1}{k}\Bigg \} \end{aligned}$$

for all $k\in {\mathbb {N}}$. Since $g_k\chi _\Omega \in BV(\Omega )$ with $|Dg_k|(\Omega )=|Df|(\Omega )$ for all $k\in {\mathbb {N}}$, by [4,  Definition 3.20 and Proposition 3.21] there exists a sequence $(h_k)_{k\in {\mathbb {N}}}\subset BV({\mathbb {R}}^n)$ such that

$$\begin{aligned} {{\,\mathrm{supp}\,}}h_k\subset A_{2k}, \quad h_k=g_k \text { in } \Omega , \quad |Dh_k|(\partial \Omega )=0 \end{aligned}$$

for all $k\in {\mathbb {N}}$ and

$$\begin{aligned} \lim _{k\rightarrow +\infty }\int _{A_{2k}\setminus \Omega }|h_k|\,dx=0 \end{aligned}$$

(the latter property easily follows from the construction performed in the proof of [4,  Proposition 3.21] Now let $(v_k)_{k\in {\mathbb {N}}}\subset C^\infty _c({\mathbb {R}}^n)$ be such that ${{\,\mathrm{supp}\,}}v_k\subset A^c_k$ and $0\le v_k\le 1$ for all $k\in {\mathbb {N}}$ and $v_k\rightarrow \chi _{ {}\overline{\Omega }^c}$ pointwise in ${\mathbb {R}}^n$ as $k\rightarrow +\infty$. We can thus set $f_k:=h_k+v_kg_k$ for all $k\in {\mathbb {N}}$. By [4,  Propositon 3.2(b)], we have $v_kg_k\in BV({\mathbb {R}}^n)$ for all $k\in {\mathbb {N}}$, so that $f_k\in BV({\mathbb {R}}^n)$ for all $k\in {\mathbb {N}}$. Since we can estimate

$$\begin{aligned} |f_k-f|&\le |h_k-f\chi _\Omega |+|v_k-\chi _{\Omega ^c}|\,|g_k|+|g_k-f|\,\chi _{\Omega ^c}\\&=|h_k|\,\chi _{A_{2k}\setminus \Omega }+|v_k-\chi _{\Omega ^c}|\,|g_k|+|g_k-f|\,\chi _{\Omega ^c} \end{aligned}$$

for all $k\in {\mathbb {N}}$, we have $f_k\rightarrow f$ in $L^1_{{{\,\mathrm{loc}\,}}}({\mathbb {R}}^n)$ as $k\rightarrow +\infty$ (because $\partial \Omega$ is Lipschitz, so ${\mathscr {L}}^{n}(\partial \Omega ) = 0$), with $f_k\rightarrow f$ in $L^1({\mathbb {R}}^n)$ as $k\rightarrow +\infty$ if $f\in L^1({\mathbb {R}}^n)$. By construction, we also have

$$\begin{aligned} |Df_k|(\Omega )=|Dh_k|(\Omega ) \quad \text {and}\quad |Df_k|(\partial \Omega )=|Dh_k|(\partial \Omega )=0 \end{aligned}$$

for all $k\in {\mathbb {N}}$. The proof is complete.$\square$
